# Brazilian Society of Angiology and Vascular Surgery guidelines on peripheral artery disease

**DOI:** 10.1590/1677-5449.202300592

**Published:** 2024-10-28

**Authors:** Fabiano Luiz Erzinger, Afonso César Polimanti, Daniel Mendes Pinto, Gustavo Murta, Marcus Vinicius Cury, Ricardo Bernardo da Silva, Rodrigo Bruno Biagioni, Sergio Quilici Belckzac, Edwaldo Edner Joviliano, Walter Junior Boin de Araujo, Julio Cesar Peclat de Oliveira

**Affiliations:** 1 Hospital Erasto Gaertner, Serviço de Cirurgia Vascular, Curitiba, PR, Brasil.; 2 Sociedade Brasileira de Angiologia e de Cirurgia Vascular – SBACV-PR, Curitiba, PR, Brasil.; 3 Instituto da Circulação, Curitiba, PR, Brasil.; 4 Sociedade Brasileira de Angiologia e de Cirurgia Vascular – SBACV-SP, São Paulo, SP, Brasil.; 5 Sociedade Brasileira de Angiologia e de Cirurgia Vascular – SBACV-MG, Belo Horizonte, MG, Brasil.; 6 Hospital Felicio Rocho Ringgold, Cirurgia Vascular, Belo Horizonte, MG, Brasil.; 7 Rede Mater Dei de Saúde, Cirurgia Vascular, Belo Horizonte, MG, Brasil.; 8 Instituto de Assistência ao Servidor Público Estadual de São Paulo – IAMPSE, Serviço de Cirurgia Vascular e Endovascular, São Paulo, SP, Brasil.; 9 Pontifícia Universidade Católica do Paraná – PUCPR, Cirurgia Vascular, Curitiba, PR, Brasil.; 10 Santa Casa de Londrina, Cirurgia Vascular, Londrina, PR, Brasil.; 11 Sociedade Brasileira de Radiologia Intervencionista e Cirurgia Endovascular – SOBRICE, São Paulo, SP, Brasil.; 12 Instituto de Aprimoramento e Pesquisa em Angiorradiologia e Cirurgia Endovascular – IAPACE, São Paulo, SP, Brasil.; 13 Universidade de São Paulo – USP, Faculdade de Medicina de Ribeirão Preto – FMRP, Ribeirão Preto, SP, Brasil.; 14 Universidade Federal do Paraná – UFPR, Hospital das Clínicas – HC, Curitiba, PR, Brasil.; 15 Universidade Federal do Estado do Rio de Janeiro – UNIRIO, Departamento de Cirurgia Vascular, Rio de Janeiro, RJ, Brasil.

**Keywords:** peripheral artery disease, diagnosis, treatment, metanalysis

## Abstract

Patients with peripheral artery disease and generalized atherosclerosis are at high risk of cardiovascular and limb complications, affecting both quality of life and longevity. Lower limb atherosclerotic disease is associated with high cardiovascular morbidity and mortality and adequate management is founded on treatments involving patient-dependent factors, such as lifestyle changes, and physician-dependent factors, such as clinical treatment, endovascular treatment, or conventional surgery. Medical management of peripheral artery disease is multifaceted, and its most important elements are reduction of cholesterol level, antithrombotic therapy, control of arterial blood pressure, control of diabetes, and smoking cessation. Adhesion to this regime can reduce complications related to the limbs, such as chronic limb-threatening ischemia, that can result in amputation, and the systemic complications of atherosclerosis, such as stroke and myocardial infarction.

## STANDARDIZATION OF NOMENCLATURE

The objective is to establish a standard nomenclature for use in these guidelines and to consolidate the concepts employed.

***1. Technical success*:** defined as successful use of a device, whether balloon, stent, or atherotome, to restore vessel patency with less than 30% residual stenosis. While the subjective angiographic criterion is a valid measure of technical success; in studies of new devices in particular, it is important to have an independent analysis conducted by a core lab. Objective measures of technical success include intravascular ultrasonography and pressure gradient across the lesion less than 10 mmHg, the second of which can sometimes be used for aortoiliac interventions.^1,2^

***2. Hemodynamic success*:** defined as a pressure gradient across the lesion of less than 10 mmHg. Not often used for infrainguinal interventions. From a clinical point of view, calculating the preoperative and postoperative ankle-brachial index (ABI) provides an objective measure.^3^ An increase of at least 0.15 in the index obtained using the foot or ankle arteries (dorsalis pedis, posterior tibial/plantar, or external malleolar) indicates hemodynamic success. Similarly, a reduction of more than 0.15 in this index is an indirect indicator of > 50% stenosis in the treated segment.^3^ When available and applicable, a 0.10 increase in toe-brachial index is also indicative of hemodynamic success.^4^ Using an ultrasonographic criterion, hemodynamic success is defined as confirmation of an increase of at least 50% in peak systolic velocity in the treated segment.^3^

***3. Limb salvage*:** defined as preservation of the limb, irrespective of death. As such, among populations with high postoperative mortality and high technical success, it is sometimes possible to find Kaplan-Meier curves showing limb salvage exceeding overall survival. This type of result is the reason why use of this term has been progressively substituted by the outcome amputation-free survival.

***4. Minor and major amputation*:** minor amputation should not be considered an adverse outcome after revascularization of a limb with tissue loss. It is performed in order to enable good wound healing, with the objective of limb salvage. From this perspective, a minor amputation is understood as one in which the surgery allows enough of the foot to be saved to enable walking without the need for a prosthesis. In general, minor amputations are performed at the transphalangeal or transmetatarsal levels. As such, higher level foot amputations, including Syme and Chopart amputations, are classified as major amputations. Revascularization procedures performed to enable below-the-knee amputation rather than above-the-knee amputation are still classified as major amputations and, consequently, are not defined as “limb salvage”.

***5. Amputation-free survival*:** defined as a composite outcome because for a patient to achieve this outcome, it is necessary that they are alive and that their limbs have been saved. In contrast with limb salvage, if the patient dies during postoperative follow-up, an adverse outcome is recorded on the date of death, irrespective of whether the limb that underwent intervention was saved. Similarly, if a major amputation is needed during follow-up and the patient survives, an adverse outcome is recorded on the date of the major amputation.

***6. Patency*:** for interventions, a segment is considered patent if one of the following criteria is met:

- Patency of the treated segment, assessed by imaging exam, whether angiotomography, angiomagnetic resonance imaging, digital angiography, or Doppler ultrasonography;
- Presence of a palpable pulse downstream of the treatment site, compared with pulse absent preoperatively;
- Postoperative increase of 0.15 in ABI or at least 50% increase in peak systolic velocity downstream of the treatment site.

For scientific publications, criteria B and C are accepted, but are considered weak and fairly subjective. Undoubtedly, imaging exam assessment is the best criterion for demonstrating treated segment patency. While objective, ABI may increase by more than 0.15 without necessarily demonstrating segment patency. This type of situation is particularly likely in endovascular interventions involving multiple segments, in which it is possible that the index will increase despite occlusion of one of the treated segments.

***7.* *Primary, primary assisted, and secondary patency*:** primary patency describes uninterrupted maintenance of flow through a segment that has undergone intervention, or the interval of time elapsed between the initial intervention and an adjuvant procedure needed to maintain patency. As such, patients who have stenosis exceeding 50% in the treated segment, but who are not subjected to reintervention, are included in the primary patency concept. In some publications, the researchers stipulate that primary patency is uninterrupted patency and also presence of < 50% stenosis in the treated segment. This is also an acceptable concept, but in general it involves mentioning this criterion in the study methodology.

When an intervention is performed with the objective of correcting post-intervention restenosis, ensuring maintenance of primary patency, i.e., without prior occurrence of occlusion, the applicable concept is assisted primary patency. In turn, secondary patency is patency obtained with a secondary procedure performed after occlusion of the segment originally treated. As such, secondary patency procedures include pharmacological, mechanical, and pharmaco-mechanical thrombectomy of post-intervention occlusions.

Depending on the outcome definitions chosen by the researcher, it should be pointed out that performing recanalization of sites that have undergone angioplasty and become occluded, without adjuvant thrombectomies, is better considered as a de novo intervention or redo, rather than a secondary patency procedure.

***8. Target lesion*:** any type of lesion that has been treated or undergone a treatment attempt. In general, treated segments are analyzed individually, i.e., if a patient undergoes superficial femoral artery (SFA) stenting and also undergoes concurrent anterior tibial artery angioplasty, technical success and patency data should ideally be reported for each of the treated vessels, rather than as a composite outcome. By definition, the target lesion includes the segments 10 mm proximal and 10 mm distal of the treatment site.

***9*. *Target lesion revascularization (TLR)*:** this term was originally conceptualized for coronary endovascular interventions, specifically as a method of reporting restenosis. Conceptually, it refers to any type of reintervention conducted on the originally treated segment, including the 10 mm proximal and distal of the initial intervention. If the site originally treated becomes occluded, and, for example, an arterial bypass is constructed with an anastomosis beyond this point, this is defined as target lesion revascularization. In general, outcomes related to this concept are reported as survival free from target lesion revascularization, using Kaplan-Meier curves.

Considering the above, some guidelines, including those of the Society of Vascular Surgery (SVS), do not recommend using TLR as a primary marker of success of interventions for revascularization of the lower limbs (LLs).^2^ Very often, assessment according to TLR is linked to reinterventions that are not guided by patient clinical status, but by presence of > 50% restenosis. Patients with restenosis whose peripheral lesions have already healed often do not undergo reintervention. In view of this, the concept of clinically driven TLR was developed to describe cases when peripheral lesions have not healed and reintervention is performed at a site with > 50% restenosis.^1^

***10.* *Major adverse outcomes*:** many studies employ the technique of summing composite outcomes with the objective of demonstrating the superiority of one technique over another or with the objective of ensuring the efficacy/safety of a given procedure. It is common for researchers to report composite outcomes, which include major adverse event-free survival, major adverse limb event-free survival, etc. There is no rule to defining these major adverse events, which are often defined by the researchers themselves in the study methodology. As such, major adverse events could mean need for reintervention and/or major amputation and/or death, for example.

## OBJECTIVE

The objective of these guidelines is to present comprehensive, optimized, evidence-based care recommendations for patients with lower limb peripheral artery disease (LLPAD), offering trustworthy and transparent clinical practice recommendations published by those who took part in their development, and on which the industry has had no direct influence regarding the clinical content or the recommendations – which is essential for a trustworthy and independent document. The guidelines apply to adults with asymptomatic or symptomatic disorders of the peripheral arterial circulation caused by atherosclerosis, and are not therefore applicable to children. Treatment strategies for non-atheromatous causes of peripheral arterial occlusion processes (vasculitis, dissection, giant-cell arteritis, fibromuscular dysplasia, radiogenic stenosis, and entrapment syndromes) should be differentiated from atherosclerotic stenoses/occlusions and are not the focus of these guidelines. Neither are emergency situations, such as acute arterial occlusions or traumatic injuries.

These guidelines are intended to support the medical team and patients in taking decisions on the best diagnostic and therapeutic methods for patients with PAD and help them along the action and decision pathways. They can also be used as an up-to-date source of information for public health institutions and government policies. Guidelines published by scientific medical societies are not legally binding on physicians and, therefore, cannot be employed to determine responsibility or relieve physicians of responsibility. What legally constitutes a medical standard for treatment of a particular patient can only be determined by individual assessment of that same patient. Therefore, these guidelines do not exempt physicians from their obligation to care for their patients individually, evaluating each patient’s overall situation.

## INTRODUCTION

Peripheral artery disease comprises a diverse group of disorders that lead to progressive stenosis, occlusion, or aneurysmal dilatation of the aorta and its non- coronary branches, including the carotid branches of the upper extremities, the visceral branches, and the arteries of the lower limbs.^5,6^ The prevalence of PAD is approximately 12% of the adult population, affecting slightly more men than women, and affecting more than 200 million people worldwide, depending on age.^7-9^ It is frequently asymptomatic, under-diagnosed, and undertreated, and is one of the greatest causes of cardiovascular (CV) and cerebrovascular morbidity and mortality. Activation of coagulation and endothelial stimulation are significantly increased in these patients, with elevated platelet activation, abnormal fibrinogen levels, and generation of thrombin and fibrin,^10^ and these characteristics explain the relationship between the severity of arterial disease and the difficulties involved in its treatment and follow-up.^11^ In particular, patients with chronic limb-threatening ischemia (CLTI) have deregulation of procoagulatory, anticoagulant, and fibrinolytic pathways, with reduced levels of natural anticoagulants (proteins C and S) and coagulation factors FIX, FXI, and FXII,^12^ which explains the frequency of arterial thrombosis.^13^ In the Framingham study, 75% of patients with PAD died from cardiovascular events, and it was observed that mortality was two to three times greater among patients with intermittent claudication (INC).^14^ The most common presentation of PAD is LLPAD, with symptoms of cramps, tiredness, dormancy, or weakness of the legs, hips, thighs, or calf muscles during certain activities. Approximately 50% of these patients exhibit symptoms such as INC or others, which can progress to acute limb ischemia.^15^ However, the disease is very often under-diagnosed because of the absence of limb-related ischemic symptoms, or even because of atypical symptoms, or due to the characteristic slow progression of the disease, which patients very often confuse with lack of physical fitness. The risk of LLPAD increases when there are associated cardiovascular risk factors, such as hypertension, dyslipidemia, smoking, and diabetes,^16-18^ which explains the higher frequency of LLPAD among patients with cardiac diseases including coronary artery disease (CAD), heart failure, and atrial fibrillation (AF). This demands a multidisciplinary approach and has important therapeutic and prognostic implications for appropriate treatment. Patients with LLPAD are therefore at high risk of major adverse cardiovascular events (MACE), such as non-fatal stroke (cerebral vascular accident), non-fatal myocardial infarction, and cardiovascular mortality over the long term and are at high risk of suffering major adverse limb events (MALE), such as severe limb ischemia, gangrene, functional impairment, and amputation, with 1-year mortality of approximately 50%.^19^ Treatment and control of LLPAD includes reduction of cholesterol, control of arterial blood pressure (BP) and glucose levels, therapy with physical exercises, and smoking cessation. Best treatment may initially be pharmacological, using antiplatelet drugs and oral anticoagulants, for later indication of surgical treatment.^20^

## METHODOLOGY

The subject and scope of these guidelines were defined by the Board of Directors of the Brazilian Society of Angiology and Vascular Surgery (SBACV, Sociedade Brasileira de Angiologia e Cirurgia Vascular) and total autonomy was granted to the coordinators responsible for organizing the guidelines. Coordinators were designated on the basis of their research and clinical experience and charged with reviewing and updating the major published PAD guidelines on the basis of a literature search primarily focused on systematic reviews and meta-analyses from 2015 to 2022. The search was run on PubMed using the keywords “peripheral artery disease”, “diagnosis”, “treatment”, and “meta-analysis”. The consensus-building process was conducted via confidential electronic communications between individuals or pairs of study group members, to avoid bias introduced by personal experience. After each section was complete, the editors reviewed the recommendations, and their individual comments were submitted for approval by the other team members. When more than 2/3 of the members agreed, they were confirmed as correct and adequate and when agreement was not reached on a recommendation it was reviewed again. Diseases of the aorta and iliac arteries, non-atherosclerotic diseases of the lower limbs, and emergencies (traumas and acute arterial occlusion) were excluded and the guidelines are restricted to the adult population. The Grading of Recommendations Assessment, Development and Evaluation (GRADE) system was adopted. GRADE is a tool developed for use by a collaborative group of researchers, aiming to create a universal, transparent, and sensible system for practical determination of the quality of evidence and strength of recommendations. The system is currently used by the World Health Organisation World Health Organisation, the National Institute for Health and Clinical Excellence, and the Centers for Disease Control and Prevention.^21^ A strong recommendation (Grade 1) means that the guideline developers are confident in the analysis of the balance between benefit and harms and that the recommendation should be followed for the majority of patients. A conditional recommendation (Grade 2) implies less certainty between the advantages and disadvantages of an approach. The evidence level supporting each recommendation is rated as high quality (A), moderate quality (B) or low quality (C), and very low quality was also grouped with low quality (C), following the practice adopted by UpToDate. After final review of the recommendations and approval by the guidelines organizing team, they were compiled into a single document and sent to the Scientific Board of Directors of the national chapter of the SBACV for final review and approval of the document, before submission for publication.

## DIAGNOSIS OF LLPAD

LLPAD is prevalent in the population over the age of 50 years, presenting with symptoms suggestive of claudication in the lower limbs. However, this symptom is not always clearly present and it is important to perform differential diagnosis to rule out other causes of leg pain (Table 1), starting with a full clinical history and a physical examination focused on the most relevant signs and symptoms. The Edinburgh Claudication Questionnaire (Table 2) is a questionnaire (validated for LLPAD) that is used in epidemiological studies and can help with investigation of LL claudication. It was tested in 300 individuals over the age of 55 years, demonstrating sensitivity of 91.3% (95% confidence interval [95%CI], 88.1-94.5%) and specificity of 99.3% (95%CI 98.9-100%).^23^ It was also used in a study in Brazil with similar results for sensitivity and specificity.^22^ Only 5 to 10% of the patients with LLPAD exhibited classic symptoms of INC;^24^ other patients exhibited nonspecific discomfort in the back, buttocks, or legs; and some patients could be asymptomatic. Typically, patients with claudication of vascular origin exhibited cramps and muscle pain (calf or buttocks) when walking a specific distance, having to stop to be able to continue walking. This differs from chronic limb-threatening ischemia (CLTI), formerly known as critical ischemia, which provokes pain even at rest, or is associated with gangrene or ulceration of the lower extremity, identifying patients with more severe forms of the same disease.

**Table 1:** Different causes of leg pain.

Vascular Origin	Peripheral artery disease, chronic limb-threatening ischemia, chronic venous insufficiency, deep venous thrombosis, non-atherosclerotic arterial disease (for example: popliteal artery entrapment syndrome).
Neurogenic Origin	Spinal canal stenosis, peripheral neuropathy, radiculopathy, spondylolisthesis.
Musculoskeletal Origin	Arthritis of the hips or knee, symptomatic Baker’s cyst, exertion-related chronic compartment syndrome, stress fracture, muscle spasms, or cramps.
Others	Restless leg syndrome, vasculitis, oncological diseases and their treatments.

**Table 2:** Edinburgh claudication questionnaire (Portuguese version).^22^

1. Do you feel pain or discomfort in your leg(s) when you walk?
Yes
No
2. Does this pain start when you are static or sitting?
Yes
No
3. Do you have this pain when you climb a hill or walk fast or run?
Yes
No
4. Do you have this pain when you walk at your normal speed, on the flat?
Yes
No
5. What happens when you stop?
It generally continues for more than 10 minutes?
It generally disappears in 10 minutes or less?
6. Where do you feel this pain or discomfort?
(There is a figure representing the lower limbs for the patient to indicate the site)

Classic characteristics of claudication include:

1. Muscle pain, typically involving the calf muscles or a muscle group distal to an arterial stenosis or occlusion and frequently described as cramp;
2. Pain that only appears when the muscle is exercised, during walking or other physical activities;
3. Pain that generally disappears within 10 minutes after ceasing exercise or resting.

After taking a complete clinical history, physicians should conduct a focused peripheral vascular physical examination, during which it is necessary to conduct specific additional tests to confirm the diagnosis of LLPAD, considering other different causes of leg pain (Table 1).^25^

The most widely used of the many different additional tests is the ankle-brachial index (ABI), which is a cheap and noninvasive test involving measurement of systolic blood pressure (SBP) at the arm (over the brachial artery) and ankle (over the dorsalis pedis artery or posterior tibial artery) while the patient is in the supine position. Using a continuous wave Doppler machine, ABI is calculated by taking the highest systolic pressure value for either ankle and dividing it by the highest pressure measured in the right arm, and then for the left, respectively. If the ABI is less than 0.9, it is suggestive of LLPAD.^26,27^ The incidence of LLPAD varies according to the prevalence of risk factors for the disease, such as smoking, hypertension, hypercholesterolemia, and diabetes mellitus.^22^ Many of these patients also have medial arterial calcification, which is an important condition that is more prevalent in patients with diabetes, chronic kidney disease (CKD), and advanced age. These conditions make the arteries less compressible,^28^ which can falsely normalize or elevate the ABI to a value exceeding 1.4, making the test less reliable. Since the distal arteries are less often affected by atherosclerotic disease, the toe-brachial index (TBI) can be calculated by measuring the BP at the arm and the great toe, and dividing them to obtain a TBI measurement for each leg. The ABI and TBI are the most widely studied tests for diagnosis of LLPAD, but these studies lack precision with regard to the characteristics of the populations studied, with great variability of symptoms and risk factors compromising their accuracy (for example, sensitivity from 45 to 100% and specificity from 16 to 100% for TBI).^27^ There is also a lack of consistency in relation to cutoff values and to the method of performing the TBI for establishing a diagnosis of LLPAD. A value of less than 0.60 is the most widely accepted cutoff in such studies.

### Treadmill test

Tests based on exercise on a treadmill are recommended because they provide objective evidence of the magnitude of functional limitation due to claudication and in order to measure the response to treatment.^29^ It is recommended that a standardized exercise protocol be used, with fixed or progressive load. One meta-analysis study reported that the most reliable protocol uses a graded increase and the absolute distance of claudication. A fixed load protocol may be recommended if an adjustable treadmill is not available, using a positive inclination of 12° and 3.2 km/h.^30^ When the treadmill test must be stopped because the patient cannot walk any further, the result is defined as the maximum walking distance, which is useful for determining whether leg pain is of ischemic origin or not. A > 30 mmHg reduction in SBP at the ankle after exercise or a >20% reduction in ABI after exercise are diagnostic of LL arterial disease (Chart 1).^26^

**Chart 1:** Recommendation on the use of TBI in the assessment of PAD.

**SBACV Recommendations:**	
1 It is suggested that ABI should be used for screening asymptomatic adults over the age of 50 years who have risk factors for PAD (such as smoking or diabetes).	2C
2 It is recommended that ABI and/or TBI should be used to confirm a diagnosis of PAD in patients with symptoms of PAD.	1B
3 It is suggested that the toe-brachial index should be used as an adjuvant test for patients with symptoms of PAD and calcified arteries to confirm a diagnosis of PAD.	1C

## MANAGEMENT OF LLPAD

### Smoking

Smoking cessation is considered of great value, not only because of its effect on vascular disease, but also because of the profound effect on prevention of many cancers and of chronic obstructive pulmonary disease. Smoking is considered one of the most important cardiovascular risk factors. It is associated with development and progression of PAD and its major lower limb adverse events, in addition to complications such as stroke, myocardial infarction, and CV death. Smoking cessation is therefore essential to support prevention and reduction of these harms (Chart 2).^31,32^

**Chart 2:** Recommendation on the importance of smoking cessation in LLPAD.

**SBACV Recommendations:**	
4 Smoking cessation is recommended to prevent LLPAD and prevent MACE and MALE in patients with LLPAD.	1B

In those seeking to stop smoking, in addition to behavioral counseling, pharmacological treatment should be considered, ranging from nicotine replacement therapy (NRT), with gum and patches, to bupropion and varenicline. Electronic cigarette containing nicotine (ECCN) could also be considered, taking note that there is no robust evidence proving results for reduction of cardiovascular risk or pulmonary safety.^33,34^ Behavioral therapy supported by pharmacotherapy increases quitting rates by an average of 20% in the first 6 months. When compared with patients who do not receive therapy, the quit rate is 17%,^35^ with highly variable efficacy data in the literature, as shown in a Cochrane review. There are few drug-based therapies and limited published data, particularly with regard to bupropion and varenicline. A combination of bupropion with NRT or with varenicline apparently improves quitting rates, but not significantly. In a meta-analysis of 267 studies with 101,804 participants, varenicline and combination NRT (i.e., combining two types of NRT, such as patches, pills, sprays, pastils, and inhalers) versus placebo proved the most effective of all drug-based interventions, accepting nausea as a frequent side effect.^36^ ECCN have also been recommended for reduction of a smoking habit. A recent Cochrane review comparing ECCN with NRT showed a positive effect on quitting, with a relative risk (RR) of 1.53 and 95%CI of 1.21-1.93, resulting in three additional quitters per 100 after 6 months.^35^

Interventions for smoking cessation employing behavioral counseling, with or without NRT, or in a community intervention program to promote smoking reduction, suggest that they should have a minimum duration of 6 months^37^ to increase the likelihood of smoking cessation;^38^ but additional studies are needed in this area to confirm the data. Notwithstanding, intensive counseling has benefits for smoking cessation, suggesting it is an important and effective smoking cessation strategy for patients with PAD (Chart 3).^39^

**Chart 3:** Recommendation for interventions to stop smoking.

**SBACV Recommendations:**	
5 Smoking cessation interventions ranging from intensive counseling to NRT, bupropion, and varenicline are recommended.	1A

### Diabetes

Patients with concomitant diabetes and LLPAD have three to four times greater mortality and a five times greater amputation rate than patients without diabetes.^40^ The choice of anti-hyperglycemic agents in patients with LLPAD should be individualized and one of the treatment objectives is reduction of CV risk and reduction of major adverse limb events.^41^ Unfortunately, very few hypoglycemic medications have been studied in patients with LLPAD, although some have proven more promising than others. Metformin is the first line oral antidiabetic in diabetics with LLPAD and while data on this application are scarce, studies do demonstrate a positive effect on CV survival, but not on prevention of amputation.^42,43^ In the cardiovascular assessment study of canagliflozin (Canvas), this drug was associated with an increased risk of amputation among patients with diabetes, which was not observed with other SGLT-2 inhibitors such as empagliflozin and dapagliflozin. Two large meta-analyses did not report significantly increased overall risk of amputation with SGLT-2 inhibitors as a class, but rather a specific problem with use of canagliflozin, which is not recommended for diabetic patients with associated LLPAD.^44,45^ Along the same lines, studies using SGLT-2 inhibitors with LLPAD endpoints observed that the medications most associated with positive outcomes were empagliflozin and liraglutide, which were shown to reduce amputations. and therefore these substances should be considered in addition to metformin in patients with diabetes and known LLPAD.^46,47^ Another example is the selective incretin based dipeptidyl peptidase 4 (DPP-4) inhibitors used in diabetes, which did not demonstrate reduction of MACE or MALE in patients with LLPAD. However, when combined with metformin, there was a 16% reduction in development of PAD in type 2 diabetics, with a 35% reduction in amputations.^48,49^ In the majority of adults with DM, the objective is to maintain hemoglobin A1c glucose-levels at < 7%.^50-53^ However, less rigorous targets (for example, hemoglobin A1c < 8%) may be appropriate for individuals with vascular complications or limited life expectancy.^54^ Patients with type 2 DM and abnormal renal function treated with metformin may be at higher risk of contrast-induced nephropathy and lactic acidosis. Considering that this is still the subject of continuous debate, it is reasonable to suspend metformin for 24 to 48 hours before and after administration of an iodinated contrast agent (Chart 4).^55,56^

**Chart 4:** Recommendation for care for diabetic patients related to PAD.

**SBACV Recommendations:**	
6 It is recommended that patients with diabetes should be screened for PAD.	1B
7 Adequate control of diabetes is recommended for patients with PAD.	1B
8 It is recommended that all patients with PAD should be effectively treated if there is a proven diagnosis of diabetes. In type 2 diabetes, use of empagliflozin and/or liraglutide should be considered in addition to metformin.	1B
9 It is suggested that patients with PAD and diabetes may benefit from use of a DPP-4 inhibitor.	2C
10 Control of type 2 DM in patients with chronic ischemia and threatened limbs should target hemoglobin A1c < 7%.	2B
11 It is recommended that patients with PAD and type 2 diabetes should be given an SGLT-2 inhibitor versus usual diabetes control, since MACE is reduced, without any risk of increased amputation rate.	1C

### Dyslipidemia

Aggressive reduction of lipids using statins is necessary to reduce the main cardiovascular and cerebrovascular adverse events and, consequently, reduce overall and cardiovascular mortality. There is robust evidence to support this intervention with the objective of preventing MALE, but the evidence is not so clear with regard to proof of improved pain-free walking time.^57-60^ Elevated total cholesterol concentrations, low density lipoprotein (LDL) cholesterol, triglycerides, and lipoprotein and also reduced high density lipoprotein (HDL) are independent risk factors for development of PAD.^61^ The benefits of use of statins to control cholesterol are so important for prevention of vascular events and overall mortality that Cochrane analyses endorsed the benefits, cost-benefit, and improved quality of life associated with statins even in low-risk patients, despite the undesirable side effects.^62^ The benefits of lipid reduction using statins and other lipid reducing agents in PAD are unquestionable. There is less robust evidence to support the idea that reducing lipids can also improve pain-free walking distance (claudication),^63^ improve the odds of amputation, and increase patency of bypasses.^64,65^ Patients on the REACH registry with known PAD who were given statin therapy exhibited reductions in the need for peripheral revascularization, from 21.7 to 18.2%, and in the rate of amputations, from 5.6 to 3.8%, over a 4-year period.^66^ Studies in which different doses of atorvastatin or simvastatin were given to patients with claudication observed significant improvements in pain-free walking distance or maximal walking distance compared to placebo at 3, 6, or 12 months. Although some studies only examined small cohorts of patients, they showed a homogenous and reproducible effect for both substances, irrespective of observation period.^57,67^ In patients with critical extremity ischemia after venous bypass surgery, statins significantly improved 1-year survival in the Project of Ex-Vivo vein graft Engineering via Transfection III (PREVENT III) study.^68^ Patients with PAD should have serum low density lipoprotein cholesterol (LDL-C) reduced to < 70 mg/dL, or reduced by >50% if their baseline LDL-C level was from 70 to 135 mg/dL, to achieve reductions in mortality and CV events.^58,69,70^ Additional considerations about coadjuvant treatments such as ezetimibe and evolocumab^71^ may be necessary in patients with difficulty reducing lipid levels and risk of de novo CV events. Patients with PAD are therefore considered high-risk patients and should be given “intensive” treatment with statins, with the objective of reducing cardiovascular risk effectively and tolerably. Medical care should be focused on monitoring these patients, since non-compliance has proven a considerable problem in clinical practice (Chart 5).

**Chart 5:** Recommendation on the use of statins for patients with PAD.

**SBACV Recommendations:**	
12 Statins are recommended for secondary prevention in all patients with PAD who tolerate these drugs.	1A
13 In addition to general prevention, statins are also indicated for improving walking distance.	1B
14 For patients with PAD, it is recommended that LDL should be reduced to 70 mg/dL or reduced by 50% if pre-treatment levels are 70-135 mg/dL.	1A

### Systemic arterial hypertension

In general, systemic arterial hypertension (SAH) is associated with an increased prevalence of PAD^72^ and the importance of the contribution made by elevated BP to LLPAD incidence increases as age increases. A large-scale population study with 4.2 million adults demonstrated that men aged 40 to 79 years with SAH had a 63% increased risk of LLPAD when their SBP increased by 20 mmHg.^73^ One of the specific objectives of treatment of hypertensive patients is to achieve control of pressure and achieve a preestablished target BP, which should be defined individually, always considering age and presence of cardiovascular disease (CVD) or its risk factors. The Brazilian arterial hypertension guidelines^74^ state that the therapeutic target for hypertensive patients with CAD should be BP <130/80 mmHg, while diastolic BP should be kept at values exceeding 70 mmHg. For hypertensive patients with heart failure or a prior stroke episode and CKD, and also for diabetic patients, antihypertensive treatment should be titrated until a target of BP <130/80 mmHg is achieved. Since concomitant CAD and advanced age are common in such situations, and also among diabetic patients, reducing BP below 120/70 mmHg should be avoided. Hypertensive patients with CKD should always be monitored for adverse events, especially when reductions in renal function and electrolyte disorders occur.^75^ Antihypertensive treatment unequivocally reduces CV events and mortality. Systolic pressure below 120 mmHg is not desirable, since it can increase the risk of acute coronary events (Chart 6).^76,77^

**Chart 6:** Recommendation in the care of arterial hypertension for patients with LLPAD.

**SBACV Recommendations:**
15 In patients with LLPAD and arterial hypertension, arterial blood pressure should be treated to reduce cardiovascular events.	1A
16 For hypertensive patients with low or moderate CV risk, the treatment target is to achieve values below 140/90 mmHg, and in those with high CV risk, target BP is < 130/80 mmHg.	1B

### Antithrombotic drugs

#### Patients with asymptomatic LLPAD

Patients with low ABI but no clinical limb symptoms or previous vascular interventions are considered to have asymptomatic LLPAD and it is difficult to find evidence of benefit to support use of aspirin in asymptomatic patients or those without PAD in other areas of the body. Patients who have other clinical atherosclerotic diseases (for example, CAD) have an increased risk of cardiovascular events, requiring a more intense antithrombotic approach.^78^ However, patients with asymptomatic LLPAD, with an ankle-brachial index below 0.9, are at increased risk of MACE and MALE^79,80^ and to date studies have been unable to demonstrate benefit from use of aspirin over the long term in this patient profile.^81^ For example, a randomized, double-blind, population study with a total of 28,980 Scottish residents did not find clinically evident cardiovascular disorders among 3,500 individuals with ABI below 0.95. After an 8.2-year period of treatment and follow-up, administration of 100 mg/day oral aspirin did not result in any difference in the rate of cardiovascular events compared with placebo.^82^ Patients with diabetes and asymptomatic PAD given 100 mg of aspirin daily did not have reduced cardiovascular event rates (lethal and non-lethal myocardial infarction (MI), stroke, cardiovascular mortality) or reduced rates of major amputations compared with patients treated with placebo.^83,84^ However, care should be taken when interpreting who is an asymptomatic patient, since atypical symptoms are common in LLPAD. In order to correctly classify these patients, it is essential to take their histories, conduct focused physical examinations, and assess them with noninvasive imaging exams, when appropriate. Patients with asymptomatic PAD in the lower limbs are frequently affected by coronary atherosclerotic disease or cerebrovascular disease and may need antithrombotic therapy for these indications (Chart 7).^85^

**Chart 7:** Recommendation for the routine use of antithrombotic therapy.

**SBACV Recommendations:**	
17 Routine antithrombotic therapy (antiplatelet or anticoagulant agents) is not recommended for patients with asymptomatic lower limb PAD only.	1A

#### Stable symptomatic LLPAD patients

Lower limb PAD is often considered to be just one manifestation of systemic atherosclerosis. The efficacy of antithrombotics for LLPAD is therefore assessed according to MACE and MALE results and the benefit of using antithrombotics is overall vascular protection; but it must always be weighed against the risk of major and/or fatal bleeding. Patients with INC who have not undergone peripheral arterial endovascular or surgical revascularization in the last 6 months (recent) and have no acute symptoms of pain at rest or tissue loss, are considered to have stable LLPAD. While single antiplatelet treatment has been the basic antithrombotic therapy for patients with symptomatic LLPAD,^86,87^ recent randomized studies testing low dose direct oral anticoagulants (DOAC) combined with aspirin, have presented new and important evidence for these patients.^88,89^ The benefit of lifelong antiplatelet treatment in patients with PAD appears convincing for prevention of CAD or cerebrovascular lesions.^86,87,90^ A meta-analysis of trials of use of antiplatelet drugs from which results were available by 1997^91^ included 135,000 patients with cerebrovascular disease, coronary disease, or LLPAD treated using antiplatelet drugs, and 77,000 controls. The antiplatelet treatment group had a 22% reduction in MACEs and taking 75 to 150 mg of aspirin per day had the same efficacy as higher doses, but with lower risk of bleeding. Another meta-analysis^86^ studied the specific benefit of aspirin in 16 studies of secondary prevention with 17,000 patients, confirming the benefit of antiplatelet drugs, with an 18.2% reduction in MACE in men and women. The Critical Leg Ischaemia Prevention Study group compared the benefits of 100 mg of aspirin per day in 185 patients with symptoms of LLPAD and ABI < 0.85 or TBI < 0.6 compared to placebo, reporting a 64% reduction in the risk of vascular events, compared with a 24% reduction in the placebo group.^90,92^ Historically, ticlopidine was investigated in several different studies of PAD patients and was shown to reduce the risk of AMI, stroke, and death from cardiovascular causes.^92^ However, the benefits are limited by frequent gastrointestinal side effects, in addition to neutropenia and thrombocytopenia. Clopidogrel is another thienopyridine derivative and was substituted for ticlopidine in later studies. It was compared with aspirin in A Randomised, Blinded, Trial of Clopidogrel Versus Aspirin in Patients at Risk of Ischaemic Events (CAPRIE), showing overall benefit in the LLPAD subset, proving its efficacy for reduction of rates of AMI, stroke, and cardiovascular mortality, with a 24% reduction in relative risk.^86,87^ The current European Society of Cardiology guidelines recommend clopidogrel rather than aspirin in patients with LLPAD.^5^ In high-risk patients with multiple risk factors and atherothrombotic manifestations (including LLPAD), a combination of aspirin and clopidogrel resulted in a higher risk of hemorrhage and no benefit.^93^ In general, the combined treatment did not result in statistically significant reductions in the risk of AMI, stroke, or cardiovascular death, so combined treatment cannot be recommended for all patients with PAD. However, data from the PEGASUS-TIMI study^94,95^ showed that, in patients with PAD and a prior myocardial infarction, which is a selected subset with high ischemic risk, dual antiplatelet therapy (DAT) with aspirin and low dose ticagrelor (60 mg twice a day) was associated with reductions in MACE and MALE and with acceptable rates of bleeding. As such, its use can be considered in patients with PAD and a prior myocardial infarction up to 3 years after the cardiac event; although data are lacking to support its indication for prolonged treatment (Chart 8).^5^

**Chart 8:** Recommendation on the use of platelet antiaggregation for patients with PAD.

**SBACV Recommendations:**	
18 Platelet aggregation inhibitors are recommended for secondary prevention of cardiovascular events in patients with symptomatic PAD.	1A
19 Clopidogrel may be preferrable to aspirin.	2B

The role of direct oral anticoagulants is currently the subject of intense investigation. The Cardiovascular Outcomes for People Using Anticoagulation Strategies (COMPASS) study is a multicenter randomized study with 7,470 individuals with stable LLPAD that found that low dose rivaroxaban (an oral factor Xa inhibitor) in combination with aspirin resulted in reduction of MACE and MALE compared with aspirin alone.^89^ Patients assigned to take rivaroxaban (2.5 mg twice a day) combined with aspirin (100 mg once a day), had 24% better overall survival and cardiovascular outcome (RR 0.76; 95%CI, 0.66–0.86), but with more major hemorrhagic events than those assigned to aspirin only (RR 1.70; 95%CI, 1.40– 2.05). Rivaroxaban alone (5 mg twice a day) did not result in better cardiovascular results than aspirin alone, but did result in more major hemorrhagic events. Clearly, decision-making must weigh the risks of CV events against the risk of bleeding. The net benefit was a 22% overall reduction of risk in the coronary disease population with stable LLPAD.^96^ An additional analysis of the LLPAD subset of the study population (patients with stable LLPAD, CAD with asymptomatic LLPAD, and stable carotid stenosis)^89,97^ found a significant additional 46% reduction in MALE and rate of amputation of the involved limb (RR 0.54; 95%CI 0.35–0.82) for aspirin 100 mg/day combined with rivaroxaban 2.5 mg twice a day, compared with aspirin 100 mg/day and placebo. The net benefit was a 28% reduction in risk for the COMPASS LLPAD subset compared with a 24% reduction in MACE in the CAPRIE subset.^86^ Therefore, among patients for whom there is greater concern with the ischemic risk, such as myocardial infarction, stroke, acute limb ischemia, or major amputation, the option could be rivaroxaban 2.5 mg twice a day in combination with aspirin. Among patients for whom the greater concern is with prevention of bleeding and minimization of the number of pills, the option could be single antiplatelet treatment.

It is also important to remember that use of rivaroxaban 2.5 mg twice a day in combination with aspirin should be avoided in patients with a strong influence from cytochrome P450 (CY3A4, CYP2J2) drug interactions or with glycoprotein-p interactions among those with liver failure, bleeding diathesis, or coagulopathy, in addition to patients with a recent stroke (< 1 month), any prior hemorrhagic stroke, and estimated glomerular filtration rate < 15 mL/min (Chart 9).^78,85^

**Chart 9:** Recommendation on the use of oral anticoagulants for patients with PAD.

**SBACV Recommendations:**	
20 Treatment with rivaroxaban 2.5 mg twice a day in combination with aspirin (80-100 mg a day) can be recommended for treatment of patients with symptomatic PAD of the lower limbs who have a high risk of ischemic events and a low risk of bleeding.	2A
21 Additional use of full-dose anticoagulants with antiplatelet treatment, with the objective of reducing MACE and MALE events in patients with stable PAD of the lower limbs, is not recommended.	1A
22 Dual antiplatelet therapy (DAT) with aspirin and clopidogrel or aspirin and ticagrelor can be considered for patients with symptomatic LLPAD and high risk of vascular events, with low risk of bleeding and contraindication to rivaroxaban.	2B

Comment on Recommendation 20:

This recommendation places greater value on a single, well-designed, randomized and controlled study rather than on several other smaller and lower quality studies. However, although this was a randomized study with robust methodology, inclusion of the specific outcome of acute limb ischemia in peripheral vascular surgery was assessed in a small study arm (389 vs. 516) for secondary events. Therefore, while use of low dose rivaroxaban appears to be promising for prevention of acute limb ischemia, we believe it would be prudent to wait for further studies to reproduce these results.

#### Postoperative use

##### Platelet aggregation inhibitors, alone or in combination

There are many endovascular and open surgery techniques available for patients with LLPAD who need revascularization, including thromboendarterectomy, thrombectomy, femoral bifurcation angioplasty, profundaplasty, and bypass procedures employing venous and synthetic grafts. When disorders are present at multiple levels, complex hybrid procedures can be employed, involving combinations of balloon dilatation and, if necessary, stenting for proximal lesions and simultaneous bypasses for distal lesions.^98^ The permeability rates of all procedures involving bypasses in the lower limbs require adjuvant treatment with platelet inhibitors, irrespective of the technique used.^99^ Around 1/3 of venous grafts performed in the lower limbs will develop problems threatening their patency in the vein itself and/or lesions involving the anastomosis region, and higher risk is observed when smaller caliber grafts or non-saphenous veins are used and when the anastomosis is infrapopliteal.^100,101^ Early occlusions after venous and synthetic bypasses are primarily caused by technical problems associated with blood flow disorders. Medium-term and late occlusions may be caused by neointimal hyperplasia of the anastomosis or the graft itself or by progression of arteriosclerosis in the native vascular bed.^102^ The high thrombogenicity of the internal surfaces of synthetic grafts is the major differentiator of occurrence of thrombotic occlusions, since venous grafts are lined with endothelium, which is less thrombogenic, whereas synthetic bypasses rarely have fully developed endothelium layers.^99^

Antiplatelet treatment is recommended to improve patency rates after surgery with below-the-knee bypasses, although it is more effective in synthetic grafts than autologous conduits.^98,103-105^ A meta-analysis of eleven randomized clinical trials conducted before 1990 showed that administration of antiplatelet drugs significantly reduces the risk of bypass occlusion, by 32%.^106^ This result was confirmed by another meta-analysis, of five studies, that analyzed aspirin (alone or in combination with other antiplatelet drugs) versus placebo in patients with infrainguinal bypasses. Bypass occlusions were observed in 28.4% of the 423 individuals who received antiplatelet treatment and in 36.6% of 393 individuals given placebo, with a relative risk (RR) of 0.78 for reduction of infrainguinal graft occlusion among patients taking aspirin.^107^ Among patients who are already on double antiplatelet treatment, the intraoperative and perioperative risks of arterial thrombosis should be balanced against individual bleeding risk. Clinical experience demonstrates that clopidogrel should ideally be discontinued 8 to 10 days before vascular surgery because of increased bleeding risk and that treatment with aspirin should be continued. However, cardiovascular risk factors such as recent drug-eluting coronary stenting must be considered.^108^ The Bypass Surgery for Peripheral Artery Disease (CASPAR) study^105^ randomized 851 individuals to receive aspirin only (75 to 100 mg) or clopidogrel combined (75 mg) with aspirin and observed no significant difference in patency rates. However, a subset analysis found a significant benefit of combined treatment for bypasses using synthetic grafts compared to those using veins, and so double platelet inhibition with aspirin and clopidogrel can be considered during the postoperative period of below-the-knee bypass surgery using prosthetic grafts. Two important studies of open revascularization include the Dutch GOOD study^109^ and the VOYAGER PAD study.^110^ In the Dutch GOOD study, treatment using monotherapy with vitamin K antagonist (VKA) (INR= 3.0-4.5), compared with monotherapy using aspirin did not significantly reduce graft occlusion or MACE but did significantly increase major bleeding rates (RR, 1.96; 95%CI, 1.42-2.71). However, subset analysis showed better permeability with VKA in venous graft bypasses (RR, 0.69; 95%CI, 0.54-0.88) and with acetylsalicylic acid for synthetic grafts (RR, 1.26; 95%CI, 1.03-1.55). The VOYAGER-PAD study assessed the safety and efficacy of DAT (rivaroxaban 2.5 mg/2vezes/day + aspirin) versus monotherapy with aspirin, started 10 days after revascularization, in 6,564 patients who underwent surgical or endovascular lower limb revascularization. During an average follow-up of 28 months with primary efficacy, acute limb ischemia, major amputation, MI, ischemic stroke, or cardiovascular death were significantly reduced using double antiaggregant therapy (DAT) versus aspirin (15.5%, vs. 17.8%, p = 0.009). With regard to safety, Thrombolysis in Myocardial Infarction (TIMI) criteria major bleeding occurred in 2.65% of the DAT group and 1.87% of the aspirin group (p = 0.07). Approximately 50% of the patients were also given clopidogrel in both study arms, for a maximum of 6 months, primarily after endovascular treatment. The beneficial effect of DAT was independent of clopidogrel, but the risk of International Society on Thrombosis and Hemostasis (ISTH) criteria major bleeding was higher when clopidogrel was administered for more than 1 month and increased over time.^111^

##### Use of heparin

Unfractionated heparin is traditionally administered intravenously before arterial clamping, with the objective of preventing thrombosis caused by stasis in the proximal and distal vascular segments and in the anastomosis segment. No randomized studies have analyzed this indication, which has probably lost its legitimacy due to long term experience. General recommendations cannot be made for intraoperative monitoring of anticoagulation, for example, based on activated coagulation time, because of a lack of data.^109^

##### Oral anticoagulation – VKA

A randomized study with 2,690 participants who underwent venous or synthetic bypass surgery compared VKA with INR 3 to 4.5 or aspirin 80 mg/d,^110^ did not demonstrate superiority of either group for antithrombotic use or between patency of femoropopliteal versus femoral-femoral bypasses (RR 0.95; 95%CI 0.82-1.11). However, a post-hoc analysis showed a significantly lower risk of occlusion among patients with venous bypass who were given VKA, whereas risk of occlusion of synthetic grafts was significantly lower for those given aspirin. These results are often used as an argument in favor of VKA after venous bypass. Patients treated with VKA exhibited major bleeding episodes significantly more often than patients treated with aspirin (RR 1.96; 95%CI 1.42-2.71). Even when INR values were adequate, study participants over the age of 72 years, with diabetes and/or arterial hypertension, exhibited increased bleeding risk. According to the evidence criteria, post-hoc analyses do not have sufficient validity to support general recommendations. Moreover, the target range used was not that usually employed, but only for situations of high risk of thromboembolism, interfering with extrapolation of these results to clinical practice. The VKAs do not improve prosthetic graft patency, but they are mildly beneficial for venous grafts.^112^ Combined treatment with aspirin and VKA is primarily considered an option for patients with difficulty accessing DOACs and should be monitored with high frequency, because of the increased risk of bleeding (Chart 10).

**Chart 10:** Recommendation on the use of SAT or combined after lower limb revascularization procedure.

**SBACV Recommendations:**	
23 Long-term SAT is recommended after revascularization.	1C
24 Long-term SAT is recommended after infrainguinal bypass surgery.	1A
25 Vitamin K antagonists can be considered after infrainguinal bypass using autologous vein.	2B
26 DAT with aspirin and clopidogrel can be considered in below-the-knee bypasses with prosthetic grafts.	2B

##### Use after endovascular procedures

Local vascular inflammation after balloon angioplasty or placement of a stent is the main factor responsible for medium and long-term restenosis processes,^113^ while early thrombosis and occlusion generally develop as a result of dissection or local arterial platelet activation. Restenosis or reocclusion are not only dependent on vascular morphology and type of endovascular technique used or stent fitted, but also on the vascular region involved. For example, stents in femoropopliteal regions are associated with an accentuatedly higher risk of restenosis than those in the iliac vessels, possibly caused by higher local inflammation levels in the muscular arteries of the femoral vascular bed, compared to the less severe inflammatory reactions in the elastic arteries of the pelvic vascular bed.^114^ Chronic inflammation of the vascular walls can also affect restenosis and risk of occlusion after successful interventions, as occurs more frequently among patients with advanced chronic renal failure and in diabetics because of metabolic disorders.^115^ Platelet activation is elevated in patients with PAD, indicating an elevated intra-arterial thrombotic tendency^116^ and antiplatelet treatment is recommended for all patients with symptomatic PAD, irrespective of secondary prophylactic interventions, in order to reduce associated cardiovascular and cerebrovascular morbidity and mortality.

Evidence-based recommendations are not clear about the most beneficial choices of dose and duration of antithrombotic drugs for antithrombotic treatments related to endovascular procedures. There is also little clarity on how to determine whether an attack dosage of clopidogrel (300 mg vs. 600 mg) should be administered before planned peripheral interventions with stenting, because no studies have analyzed this subject. A Cochrane meta-analysis including 3,529 patients assessed antithrombotic drugs for prevention of restenosis or reocclusion,^117^ finding no reduction with aspirin plus dipyridamole compared to aspirin plus placebo (OR 0.69; 95% and 95%CI, 0.44–1.10), with DAT frequently used, generally for 1 to 3 months, after endovascular procedures, with great variability in terms of duration.^118^ When it is necessary to stent infrapopliteal arteries, duration of DAT tends to be long, but evidence is not available. Duration of DAT is primarily based on extrapolation from coronary stenting, which may not be appropriate because of the probable greater residual platelet reactivity in response to adenosine diphosphate and arachidonic acid, found in patients with LLPAD.^119^ It is thus possible that patients who undergo peripheral angioplasty of the LLs have a weaker response to aspirin and clopidogrel compared with percutaneous coronary intervention patients. In the Management of Peripheral Arterial Interventions with Mono or Dual Antiplatelet Therapy (MIRROR) study, DAT using aspirin and clopidogrel was compared with aspirin alone in 80 patients after endovascular revascularization of lower limbs, finding that DAT improved rates of target lesion revascularization at 6 months (5% versus 8%), but not at 1 year.^120^ A retrospective analysis of 693 patients who underwent endovascular revascularization showed that DAT >6 months was an independent indicator of lower risk of MACE (RR 0.61; 95%CI 0.40–0.93) and MALE (RR 0.55; 95%CI 0.38-0.77), without a significant increase in bleeding.^121^ Randomized controlled studies investigated to what extent VKAs constitute an alternative to antiaggregant treatment after femoropopliteal and distal peripheral angioplasty. A total of 438 patients were randomized, and all groups treated with VKA exhibited lower rates of arterial permeability, with significantly higher bleeding rates (RR 1.79; 95%CI 1.3-4.6).^99,119,122^ Antiplatelet drugs should therefore be the first line for interventional treatments, as long as there are no other indications making VKA obligatory, primarily liked to cardiological comorbidities, such as AF. The duration of double treatment and the issue of how to determine whether clopidogrel attack doses are effective or necessary for peripheral cases both remain unclear.

Studies of treatment with cilostazol in patients with INC and femoropopliteal disease after endovascular interventions are also small, although they are prospective. In a large-scale retrospective populational analysis in the United States^123^ that analyzed data from more than 23,000 individuals with LLPAD to investigate cilostazol after open surgical and endovascular revascularizations, aspirin was administered to 20,335 patients as secondary prophylaxis, and 1,999 were additionally given cilostazol, finding significantly lower rates of restenosis and major amputation in the group given cilostazol before and/or after treatment. Other studies of additional cilostazol after femoropopliteal interventions with and without stenting found effects in the same direction. Five retrospective case series and two small prospective studies showed a reduction in restenosis rates after treatment with cilostazol.^124^ In cases with no contraindications and presence of adequate tolerability, combined treatment with aspirin and cilostazol can be considered to improve permeability and reduce amputation rates after infrainguinal endovascular treatment. A meta-analysis with 3,136 patients and mean follow-up time of 2 years for all studies showed that treatment with cilostazol improved amputation-free survival (RR 0.79; 95%CI, 0.69-0.91) and limb salvage rate (RR, 0.42; 95%CI, 0.27-0.66), reduced the need for further revascularization (RR 0.44; 95%CI, 0.37-0.52), and reduced restenosis (RR, 0.68; 95%CI, 0.61-0.76). Treatment with cilostazol also increased the patency of target lesion revascularization (RR, 1.35; 95%CI, 1.21-1.53), with no difference in mortality from all causes. Effective wound healing was found to be an inconsistent outcome measure in patients receiving cilostazol therapy (Chart 11).^125^

**Chart 11:** Recommendation on the use of antithrombotic therapy after lower limb angioplasty.

**SBACV Recommendations:**	
27 Use of DAT for at least 1 month is recommended after drug-coated balloon angioplasty or for at least 3 months after implanting a drug-eluting stent or covered stent.	1C
28 Use of DAT with aspirin and clopidogrel for at least 1 month should be considered after infrainguinal stenting.	2C
29 Antivitamin K (VKA) drugs are not recommended after angioplasty in femoropopliteal or distal territories if the only objective is to prevent restenosis or reocclusion.	2A
30 In patients who need continuous VKA for other reasons (for example, for AF) after endovascular revascularization, use of aspirin or clopidogrel, in addition to VKA, for at least 1 month should be considered if the risk of bleeding is low compared with the risk of stent/graft occlusion.	2B
31 Combined treatment with aspirin and cilostazol can be considered to improve permeability and reduce rates of amputation after infrainguinal endovascular treatment.	2A

The VOYAGER PAD study randomized patients after endovascular or open revascularization to receive rivaroxaban 2.5 mg twice a day combined with aspirin or aspirin alone, with the option to use clopidogrel additionally up to a maximum of 6 months, at the treating physician’s discretion.^111^ The combination of rivaroxaban and aspirin reduced composite MACE and MALE events (RR, 0.85; 95%CI, 0.76-0.96), primarily driven by a significant reduction in acute limb ischemia (RR, 0.67; 95%CI, 0.55-0.82), where the majority of revascularization procedures were performed because of worsening claudication (76.6%) and up to 1/3 of patients had critical limb ischemia. There was no significant difference in the primary safety outcome of major bleeding (TIMI), but the secondary safety outcome of ISTH major bleeding increased (RR, 1.42; 95%CI INC, 1.10-1.84), although there was no significant increase in intracranial or fatal bleeding. Approximately 50% of study participants were given clopidogrel, for an average of 30 days, without changing the efficacy of rivaroxaban. However, among those on clopidogrel for longer (over 30 days) there was a trend for increased major bleeding, with an absolute risk of 2.71% (RR, 3.20; 95%CI INC, 1.44-7.13), whereas < 30 days of clopidogrel was associated with a lower absolute risk of major bleeding, at 0.46%.^112^ Two thirds of this patient population underwent endovascular intervention, making this the largest study conducted to date to assess this treatment (Chart 12).

**Chart 12:** Recommendation on the use of oral anticoagulants after lower limb revascularization.

**SBACV Recommendations:**	
32 Treatment with rivaroxaban 2.5 mg twice a day in combination with aspirin (80-100 mg a day) can be recommended for patients with LLPAD after open elective revascularization, to reduce composite outcomes in patients with low bleeding risk. Use to increase patency is not recommended.	2A
33 Rivaroxaban 2.5 mg twice a day in combination with aspirin (80-100 mg a day) and with clopidogrel up to 30 days can be recommended. Without the addition of clopidogrel it can be used for longer in patients with LLPAD after elective open or endovascular revascularization.	2A

Comment on Recommendations 32 and 33:

This recommendation places greater value on a single, well-designed, randomized and controlled study with many patients rather than on several other smaller and lower quality studies. This recommendation also places great value on patients with ischemic risk in the context of acceptable increases in overall bleeding risk. Therefore, additional use of clopidogrel (75 mg a day) with rivaroxaban 2.5 mg twice a day and aspirin (80-100 mg a day) can be considered in patients undergoing complex endovascular stenting, for a maximum of 30 days, starting 10 days after revascularization. Treatment (rivaroxaban + aspirin) should preferably be continued long term in the absence of bleeding or ischemic manifestations, since the prior revascularization involves a high risk of recurrence. Therefore, while use of low dose rivaroxaban for prevention of ischemic limb events after revascularization appears promising, we believe it would be prudent to wait for further studies to reproduce these results.

### Physical activity for PAD

There is high quality evidence showing that exercise programs offer important benefits, improving maximum pain-free walking distance in people with INC. Exercise did not improve ABI and there is no evidence of effect on amputation or mortality rates. Exercise can improve quality of life when compared with placebo or routine care.^126^ Alternative exercise modalities can be useful when supervised walking exercise is not an option.^127^ Patients who underwent unsupervised exercise at home, in addition to a group cognitive-behavioral intervention, exhibited improved walking performance and physical activity, making this an option for patients without the opportunity to receive supervised exercise therapy.^128^ Structured and supervised walking training proved superior to unsupervised walking, walking training programs followed for at least 3 months under supervision demonstrated increased walking capacity and reduced claudication severity^129,130^ and were effective when performed at least three times per week for 30 to 60 minutes.^131,132^ Controlled studies in patients with claudication demonstrated increases of up to 200% in walking distance after 12 weeks of training.^133,134^ In other studies, the functional results of long term walking training also proved equivalent to vascular interventions alone.^135,136^ However, the effects of endovascular revascularization and walking training are additive^137^ and an integrated approach can be taken using both treatment modalities.

It was also demonstrated that upper limb training can benefit endothelial function in patients with LLPAD and should be attempted if walking training is not possible. Favorable prognostic factors in walking training include the following criteria: less than 1 year after diagnosis of PAD, femoral artery occlusion, and good cardiopulmonary condition. One study^138^ demonstrated that resistance training with an arm ergometer can produce improvements in pain-free walking distance and is comparable in terms of calorie consumption to treadmill training, making it an option that can complement walking training. Regular walking training also produced additional beneficial changes to glucose and lipid metabolism.^139,140^

Until vascular lesions have been successfully recanalized by endovascular interventions or conventional surgery, walking training is not effective for stenosis of deep femoral artery and occlusion of the ipsilateral SFA. Therefore, vascular recanalization should be performed for pelvic vascular lesions, lesions of the femoral bifurcation, and stenosis or occlusions of the deep femoral artery before basic treatment with walking training is introduced.^134,141^ In patients with high degree stenosis or occlusion of the popliteal artery, exercise may have a limited effect on claudication because of the limited options for collateralization. Therefore, revascularization can be recommended before physical training in this group, but the surgical result may be associated with lower patency rates over the long term. It is necessary to point out that approximately 50% of patients with LLPAD have concomitant orthopedic and/or neurological disorders and/or functional cardiopulmonary deficits, which may prevent walking training or make it impossible for them to participate in structured vascular sports groups. These comorbidities should be identified before initiating walking training and modifications should be introduced to enable the largest possible number of patients to participate in walking training (Chart 13).

**Chart 13:** Recommendation on physical activity for patients with LLPAD.

**SBACV Recommendations:**	
34 Training with supervised exercise is recommended for patients with LLPAD.	1A
35 It is recommended that patients with intermittent claudication undergo supervised training a minimum of 3 times per week, for a minimum of 30 minutes, over a minimum of 3 months.	1B
36 Unsupervised physical training is recommended when supervised physical training is unfeasible or unavailable.	1C

## TREATMENT OF LLPAD

### Indications for revascularization

Surgical treatments are associated with higher morbidity and mortality and higher costs, and, particularly in the presence of comorbidities, there are increased perioperative risks for patients. Endovascular treatments involve lower invasivity and lower rates of complications, but often require additional treatments, primarily reinterventions because of restenosis or reocclusion. Based on these advantages and disadvantages, individual risks and benefits should be assessed in order to prescribe the best treatment. The primary long-term objective of treatment for patients with chronic limb-threatening ischemia is amputation-free survival, in addition to improve healing of ulcers, improve ischemic pain, and reduce mortality rates in these patients with high cardiovascular risk. For claudicant patients, the objective of treatment is a little different, since patients seek improved quality of life because of the discomfort of pain when walking.

#### Treatment of claudication

The natural history after the first year of diagnosis of intermittent claudication generally involves an annual risk of 2 to 3% of progression to chronic ischemia, with possibility of limb loss,^142,143^ with a 1% annual risk of amputation in these patients.^143,144^ Intermittent claudication is predominantly managed by modification of risk factors for atherosclerosis associated with regular and supervised exercises,^145,146^ which results in increased walking distance and can be equally effective after endovascular or surgical revascularizations.^137^

The phosphodiesterase 3 inhibitor cilostazol is a vasodilator that inhibits proliferation of vascular smooth muscle cells and prevents platelet aggregation and is being used as a treatment to improve walking symptoms in patients with intermittent claudication and PAD. It is indicated when symptoms are persistent and impair quality of life. The mechanism by which it improves the symptoms of claudication is unclear and is probably multifactorial. The recommended dose is 100 mg twice a day. This dose should be taken at least 30 minutes before or 2 hours after breakfast or dinner.^147^

However, there is little evidence of benefit from randomized studies. In the most recent Cochrane review,^148^ it was observed that participants who took cilostazol for 3 to 6 months were able to walk longer distances before pain started than those who were given placebo. However, there is no robust evidence proving improved quality of life in these patients and the increase in pain-free distance walked is specific to each patient (Chart 14).

**Chart 14:** Recommendation on the use of cilostazol for claudication patients with PAD.

**SBACV Recommendations:**	
37 It is recommended that that cilostazol should only be considered for claudicant patients with PAD if quality of life is substantially limited and walking training is restricted, unfeasible, or ineffective.	1B
38 It is recommended that treatment with this agent is stopped if symptoms do not improve after 3 months.	1C

However, revascularization can be considered as treatment for claudication if patients continue to exhibit symptoms limiting their lifestyle or profession despite optimized clinical treatment, which is very frequent in cases with aortoiliac and popliteal artery occlusions.^135,149^ The symptomology of claudication tends to cause greater incapacity in more proximal areas, when compared to more distal occlusions.^150^ Many studies have shown similar medium-term cumulative patency for surgical and endovascular revascularization of aortoiliac and femoropopliteal segments. Endovascular intervention is associated with fewer perioperative complications, but a higher restenosis rate, which is generally managed with reintervention, also with endovascular techniques.^151-153^ The same benefit is not seen in the infrapopliteal territory, demanding a more detailed individualized assessment.^154^ When conservative treatment is unsuccessful, interventional treatment can yield quality of life improvements in the short and medium term, combined with improved ability to walk without pain.^155^ However, the criteria for surgical revascularization and/or angioplasty treatment of claudicant patients should be more rigorous, because long term mortality and limb salvage rates are not superior to conservative treatment.^156^ Patients should therefore be informed that failed interventions can have serious consequences for their limbs (Chart 15).

**Chart 15:** Recommendation for indicating surgical treatment for claudication patients with PAD.

**SBACV Recommendations:**	
39 It is recommended that initial treatment of claudicant patients should be with clinical, not surgical, methods.	1A
40 It is recommended that claudicant patients undergo revascularization when clinical treatment fails, with maintenance of severe symptoms and significant impact on quality of life, primarily associated with occlusive lesions in aortoiliac, iliofemoral, and femoropopliteal territories, including the proximal popliteal artery.	1B
41 For patients with short-distance claudication, for whom walking training is impossible or unsuccessful, and in presence of appropriate arterial lesions, interventional treatment is recommended to improve quality of life.	1C
42 Femoral-tibial bypasses are not recommended for treatment of intermittent claudication.	1B

Studies comparing supervised walking and endovascular intervention for claudication recommend a non-interventionist initial approach, because of the good response to clinical treatment in patients with stable claudication. However, the combination with endovascular intervention can yield additional benefit earlier for treadmill walking distance and quality of life, when compared with clinical treatment alone, at the cost of smaller long-term benefit.^138,157-160^ Endovascular treatment may be indicated as a treatment option in cases of assisted patency, when there has been significant stenosis of previous revascularizations.^161^ For this reason, we consider endovascular intervention to be a treatment option in these patients (Chart 16).

**Chart 16:** Recommendation on the endovascular approach for claudication patients with PAD.

**SBACV Recommendations:**	
43 Endovascular intervention in claudicant patients with lesions with significant hemodynamic repercussions is not usually recommended as a prophylactic approach, but can be considered as a treatment option in patients who have been revascularized previously.	2B


#### Chronic limb-threatening ischemia

Chronic limb-threatening ischemia is the most advanced form of LLPAD, in which patients generally have signs of arteriopathy, such as ischemic pain at rest, tissue loss or gangrene, and, in comparison with claudication, has worse natural history, with faster progression to loss of tissue and of the limb.^162^ Despite advances in pharmacological treatment and better understanding of reduction of risk factors for LLPAD,^163^ patients with CLTI continue to suffer high mortality and major amputation rates of 22% in 1 year when not treated with revascularization.^164^ In this patient profile, immediate revascularization has greater importance for the results of treatment, when compared with patients with claudication (Chart 17).^165^

**Chart 17:** Recommendation in the initial assessment for patients with chronic limb-threatening ischemia.

**SBACV Recommendations:**	
44 It is recommended that all patients with chronic limb-threatening ischemia should be urgently referred to vascular specialists, for evaluation of the need for revascularization.	1C
45 It is recommended that patients with chronic limb-threatening ischemia undergo revascularization, whether endovascular, open, or hybrid. The anatomic features of the disease, degree of ischemia, expected durability of the procedure, perioperative risk, and life expectancy of the patient should all be considered.	1C

##### Assessment and planning for patients with CLTI

Diagnostic assessment and staging with imaging methods are integral to successful treatment of patients with suspected CLTI. Nowadays, technological advances in imaging have made diagnosis of CLTI more precise, enabling better selection of patients for revascularization and planning. However, access to sophisticated diagnostic vascular imaging methods varies considerably both worldwide and in Brazil, in the different care systems, whether private or in the Unified Health System (SUS - Sistema Único de Saúde). Different routines are employed, very often without standardization, because of the limited resources available for health care.^166^ These guidelines therefore aim to establish comprehensive principles and considerations that can be used to guide and standardize assessment and treatment of patients in the most effective manner possible.

In addition to history taking and physical examination focused on correction of risk factors and local care of wounds, there is also a tendency to employ systems for classification of limbs and wounds to support decision-making and thus achieve better results for patients. A classification system developed by the SVS is being recommended and used in guidelines to stratify the results of treatment, based on the characteristics of the wound (W), ischemia (I), and presence and severity of foot infections (fI). The Wound, Ischemia and foot Infection (WIfI) classification correlates the probability of limb salvage and wound healing after revascularization (Table 3), helping with making decisions on revascularizing patients who are candidates. This classification was developed on the basis of consensus between specialists, but still needs validation. Another system to aid with parametrization of conduct is the Global Limb Anatomic Staging System (GLASS) (Tables 4 and 5),^167^ used to aggregate information and provide the most effective support for choosing the best revascularization strategy, considering the patient’s risk, limb staging, and the anatomic features of the disease.^168,169^

**Table 3:** Society For Vascular Surgery (SVS) risk of amputation classification system, WIfI.

**Component**	**Score**	**Description**		
W	0	No ulcer ischemic rest pain		
Wound	1	Small or shallow ulcer on distal leg or foot; no gangrene		
	2	Deeper ulcer with exposed bone, joint or tendon ± gangrene limited to digits		
	3	Extensive, deep ulcer, calcaneal ulcer ± calcaneal ulcer ± extensive gangrene		
I		ABI (ankle-brachial index)	Ankle pressure (mmHg)	Hallux pressure, TcPO_2_
Ischemia				
	0	≥ 0.80	> 100	≥ 60
	1	0.60-0.79	70-100	40-59
	2	0.40-0.59	50-69	30-39
	3	≤ 0.39	< 50	< 30
FI	0	No symptoms / Uninfected		
Foot Infection				
	1	Mild local infection, involving only the skin and subcutaneous tissue		
	2	Moderate local infection, involving more tissues in addition to skin or subcutaneous tissue		
	3	Severe local infection with signs of Systemic Inflammatory Response Syndrome		

**Table 4:** Original Glass classification for femoropopliteal disease.

**Femoropopliteal Classification**	
0	Mild or no significant disease (< 0%).
1	Disease involving < 1/3 (< 10 cm) of the SFA, may include single focal total occlusion (< 5 cm), not involving the SFA origin; popliteal artery with mild or no significant disease.
2	Total length of SFA disease 1/3-2/3 (10-20 cm); may include SFA CTO totaling < 1/3 (10 cm), not involving the SFA origin; focal popliteal artery stenosis < 2 cm, not involving trifurcation.
3	Total length of SFA disease >2/3 of length (>20 cm of the vessel); may include any occlusion < 20 cm that does involve vessel origin or CTO 10-20 cm long that does not involve vessel origin; short popliteal artery stenosis, 2 to 5 cm, not involving trifurcation.
4	Total length of SFA occlusion >20 cm; popliteal disease >5 cm or extending into trifurcation; any popliteal artery CTO.

SFA = superficial femoral artery; CTO = chronic total occlusion.

**Table 5:** Original Glass classification for infrapopliteal disease.

**Infrapopliteal Classification**	
0	Mild or no significant disease (< 50%).
1	Focal stenosis < 3 cm without involving the TP trunk.
2	Stenosis involving <1/3 total target artery length; may include single focal occlusion (< 3 cm), not including TP trunk or target artery origin.
3	Disease involving 2/3 total target artery length; CTO† greater than 1/3 of vessel length (may include origin of target artery, but not the TP trunk).
4	Diffuse arterial stenosis > 2/3 of vessel length; CTO > 1/3 vessel length (may include target artery origin); any CTO of TP trunk if anterior tibial artery is not the target artery.

TP = tibioperoneal; CTO = chronic total occlusion.

When deciding on the best method of revascularization to be employed for each patient, many different aspects should be assessed, not only anatomy.^170^ The complexity of revascularization strategies demands consideration of anatomic aspects of the wound, the likelihood of patient rehabilitation, surgical risk, local health service conditions, and patient preferences. Recent guidelines have used the GLASS and WIfI classifications to support choice of the most effective method of revascularization,^171,172^ which should not be used as independent guides for deciding on conduct, but to aid the complex analysis that must be done on a case-by-case basis. For example, in the case of patients with WIfI 1: in principle, they should not be revascularized, since they have sufficient blood flow for tissue healing. However, there are patients who do not achieve adequate wound healing even when classified as at very low risk of amputation or revascularization. Some biomarkers have been studied as predictors of limb loss, especially among patients with low WIfI scores.^173,174^ Longitudinal clinical assessment, i.e., patient follow-up, is essential to define indications for revascularization, since patients whose wounds are not healing even with adequate local and systemic measures should be considered for revascularization.^175^Table6^176^ demonstrates that open revascularization surgery and endovascular treatment play complementary roles, with a notable lack of consensus on the intermediate levels of clinical and anatomic complexity (in green). Studies are needed examining classifications and staging of wounds, to improve the quality of evidence on interventions in specific clinical scenarios. Patients without adequate autologous conduits should be considered separately, since this is a critical factor in determination of the probability of success and durability of revascularization bypass surgery. Even when an adequate saphenous vein is not available, bypasses using veins from the arm and spliced veins perform better than non-autologous grafts for distal treatments, which require more frequent surveillance and more reinterventions to maintain assisted primary patency.^177^ In patients with chronic limb-threatening ischemia and good surgical risk, the severity of the threat to the limb (WIfI classification), the anatomic features of the vascular lesions (GLASS classification), and the availability of autologous vein for bypass construction should be assessed in order to decide between endovascular revascularization and conventional surgery. The endovascular approach should initially be preferred for short lesions in the femoropopliteal sector.^171,172,178^

**Table 6:** Example of infrainguinal revascularization strategy in a moderate risk patient with a good vein available for bypass.

GLASS	WIfI			
1	2	3	4
III				
II				
I				
	
	Conventional surgery			
	Undetermined			
	Endovascular			
	No need for revascularization			

There is a series of benefits to using an up-to-date integrated system for classification of lower limb ischemia:

It enables precise communication between specialists;
It disseminates information among physicians who are not specialists;
It supports analysis of cases for medical auditing;
It enables classification for scientific research.

The WIfI classification is the most up-to-date, supporting clinical revascularization decision-making, classifying wounds and patients. The prognostic value of high WIfI scores is associated with amputation and death outcomes, as shown in non-randomized prospective studies (Chart 18).^179,180^

**Chart 18:** Recommendation in the treatment decision for patients with CLTI.

**SBACV Recommendations:**	
46 It is recommended that an integrated system for classification of threatened limbs (such as the WIfI) should be used to classify all CLTI patients who are candidates for limb salvage.	1C
47 Revascularization is not recommended for limbs at very low risk (for example, WIfI stage 1), unless the wound progresses or does not recede in size ≥ 50% within 4 weeks, despite appropriate control of the infection and wound.	2B
48 Initially, an endovascular approach should be adopted for patients with chronic limb-threatening ischemia and short lesions in the femoropopliteal sector (GLASS I and II).	1B
49 Revascularization should be considered for patients with an intermediate risk of amputation (for example, WIfI stages 2 and 3).	2C
50 The decision to correct inflow obstructions, during the same intervention or conduct staged limb revascularization should be based on the severity of the threat to the limb (WIfI stage) and the patient’s clinical status.	1C

Comment on Recommendation 45:

Indication of revascularization should take account of the degree of ischemia, the degree of infection, the extent of tissue damage, the anatomy of arterial obstructions, and the patient’s clinical status (WIfI classification). Arterial obstructions, particularly those below the knee, are defined with a high-quality imaging exam, of which arteriography is still the gold standard. Clinical assessment must be individualized, and patients with acceptable surgical risk and independence to perform their activities should be considered for revascularization.^181^

Comment on Recommendation 46:

In patients with disease involving multiple levels and low-level ischemia (WIfI grade 1) or limited tissue loss (WIfI grade 1), correction of the obstruction to inflow alone may be sufficient to enable healing. However, when there are proximal obstructions and moderate or high risk of amputation, the inflow correction procedure should be combined with infrainguinal revascularization. Correction of inflow can be concomitant with revascularization or staged, depending on the conditions available to the surgical team and the urgency of limb revascularization.

Pre-procedure treatment planning for patients with CLTI should include a surgical risk assessment and an assessment of saphenous vein availability, since in patients with a good quality great saphenous vein, the surgical strategy as initial intervention was associated with a 32% lower risk of major adverse limb events or death, compared to the endovascular strategy.^172^ The availability and quality of the autologous venous conduit, especially the great saphenous vein, are important criteria for bypass surgery and should be defined before revascularization decision are taken for patients with risk of limb loss.^182-184^ Other venous segments can be used, such as the small saphenous vein or upper limb veins, but the results are inferior. Ultrasound mapping helps with planning, considering that these machines are now more available in hospitals. Ultrasonographic assessment of veins can be performed by the vascular surgeon, helping with skin marking and identification of the saphenous vein or other healthy veins and optimizing planning of incisions for access (Chart 19).

**Chart 19:** Recommendation on venous mapping for patients with CLTI.

**SBACV Recommendation:**	
51 Venous mapping, when available, should be performed for all patients with CLTI who are candidates for arterial bypass surgery.	1C

#### Endovascular treatment

##### Femoropopliteal territory

The results of endovascular revascularization are heterogeneous and, in many aspects, difficult to quantify. Separating them by region, we can state that endovascular treatment of the aortoiliac segment has become the preferred option when anatomically adequate because of the low incidence of morbidity and mortality compared with open surgical options.^152^ The common femoral artery should preferably be treated with open surgery. The anatomic challenge of a flexible location below the inguinal ligament and the potential for coverage of the deep femoral artery during stenting should be weighed against the excellent results of a direct and open procedure. There is still consensus in favor of surgical intervention, but in cases with unfavorable anatomy and also in reinterventions or high surgical risk patients, endovascular techniques can be chosen.^185^ The SFA segment presents multiple challenges, including lengthening or shortening, compression, and torsion of the vessel during regular daily activity. These dynamic challenges can cause stent fractures, which can result in early occlusion and restenosis.^186^ For short lesions (< 25cm) in this territory, balloon angioplasty is comparable to conventional stenting, but for longer segments use of a stent offers better primary patency rates and lower rates of reintervention;^187^ although long-term primary patency is relatively reduced, because of intra-stent restenosis.^188^ Patients with estimated perioperative mortality of more than 5% or life expectancy less than 50% at 2 years are considered at elevated surgical risk.

For these patients, an endovascular approach can be offered initially for treatment of long lesions in the femoropopliteal sector.^169^ Covered stents tend to make neo-intimal proliferation less likely, which can offer some advantage. Non-randomized studies sponsored by the industry have shown 1-year primary patency and secondary patency of 73 and 92%.^85^ Popliteal artery lesions are frequently combined with lesions of the SFA and lesions that reach the infragenicular trifurcation. Conventional stents do not perform well in this region and if needed (for example for complex dissections), mimetic stents (with interwoven nitinol wires) are recommended in the below-the-knee segment of the popliteal artery (segment P3), since these devices offer greater resistance to extrinsic compression. This property makes fractures of this type of stent uncommon, increasing long term primary patency for occlusive lesions in the popliteal territory. However, this type of stent demands adequate vessel preparation, because inadequate preparation is associated with higher restenosis and occlusion rates.^189-192^ In cases with restenosis, many studies with stents and drug-coated balloons have demonstrated excellent results, with patency rates for 1 and 5 years of 86 and 66%, respectively, with symptoms improving in 92 and 80%.^193-195^ Randomized studies and meta-analyses have been demonstrating that drug-coated balloons also offer more benefits, compared with conventional balloons, for primary angioplasty,^194^ with even better results for reduction of restenosis rates when higher concentrations of paclitaxel are used.^196^ One additional advantage when balloon angioplasty alone is used is the fact that no stimulation remains in the arteries, in contrast with what self-expandable stents can cause, since the metal imparts a constant stress (stimulus) on the artery wall (chronic outward force), which imparts a higher risk of myointimal hyperplasia.^197-200^

There is concern with higher mortality rates reported by studies in association with drug-elution technologies. However, more recent meta-analysis data demonstrate that the risk is not greater than with use of conventional balloons, irrespective of the paclitaxel concentration.^85^

With the advent of drug-coated balloons, use of spot stenting has become restricted to areas with dissection and/or immediate elastic retraction (recoil). The concept of spot stenting is derived from indirect evidence and is still being assessed in unfinished studies (Chart 20).^198^

**Chart 20:** Recommendation for indicating endovascular treatment of the femoropopliteal territory.

**SBACV Recommendations:**	
52 Consider endovascular treatment of common femoral artery disease only in selected patients considered at high surgical risk or who have a hostile pelvis.	2C
53 Balloon angioplasty (conventional or drug-coated) should be prioritized for the popliteal artery. Optional stenting is preferably recommended for treatment of lesions in which there has been complex dissection and/or elastic recoil exceeding 30%. In such cases, and especially in segment P3, a mimetic stent is the best option.	1C
54 It is recommended that placement of self-expandable stents is restricted to spot stenting, i.e. for regions with elastic recoil exceeding 30%, complex dissection, or excentric calcification with stenosis exceeding 30%. Stenting should only be considered after balloon angioplasty (conventional and/or drug-coated).	1B
55 Drug-coated balloons can be used for treatment of intra-stent restenosis in femoropopliteal lesions.	2A
56 In patients with elevated surgical risk and long lesions (>25cm) in the femoropopliteal sector, endovascular revascularization can be considered.	2C

A meta-analysis compared 14 different treatment modalities: atherectomy, brachytherapy, cryoplasty, scoring balloon, drug-coated balloon, nitinol stent, covered stent, and combinations of these. It was demonstrated that the drug-eluting stent and covered stent are the best treatment methods at 12 and 24 months for restenosis and target lesion revascularization outcomes.^201^ Restenosis is the problem of greatest concern associated with endovascular procedures in the femoropopliteal region. Many different approaches have been developed to reduce its occurrence, such as use of stenting, with reductions in restenosis rates compared with angioplasty using balloons only. The primary patency rates for use of stents, particularly after treatment of longer segments, were just 60 to 70% after 1 year and just 30 to 60% after 2 years.^202^ The strategy of angioplasty with drug-eluting stenting can be considered for treatment of short lesions in the femoropopliteal sector, with the objective of reducing the rate of restenosis of the treated segment. This led to the development of self-expandable drug-eluting stents that improved primary patency rates to 74.8% at 2 years^203,204^ and a greater than 40% reduction in the relative risk of restenosis.^205^ It is recommended that the vessel to be treated should be prepared adequately with pre-dilation to improve drug distribution in the case of drug-coated balloons and drug-eluting stents or to improve deployment of self-expandable stents with complete expansion. This precaution can improve short and long-term patency of the treated segment. For treatment of highly calcified regions, it may be necessary to use devices to prepare the vessel, by removing plaque (with an atherotome) or modifying the plaque with a scoring balloon.^109,190,206^

##### Infrapopliteal territory

Below-the-knee endovascular treatment has been widely used over recent years, but the quality of clinical results is still unsatisfactory for infrapopliteal lesions compared with above-the-knee lesions, because of the anatomic challenges and limited specific device options.^207^ Infrapopliteal endovascular treatment has been associated with a high incidence of restenosis of the treated vessel, with primary patency rates varying from 22 to 92% at 1 year.^208^ However, despite the high rates of restenosis or occlusion, limb salvage can be achieved and maintained even with relatively low vessel patency rates.

Systematic literature reviews show that both endovascular treatment and open surgery achieve limb salvage rates of approximately 80% at 3 years.^209^ Recent technical developments have contributed to a high recanalization success rate, even for long infrapopliteal occlusions (> 10 cm) with chronic limb-threatening ischemia.^210^

Initially, use of drug-coated balloons for infrapopliteal lesions proved inferior to conventional balloons. A controlled prospective study comparing angioplasty of distal lower limb arteries using paclitaxel eluting balloons versus conventional balloons failed to confirm advantages for this type of device,^211^ demonstrating that results after 1 year did not reveal difference between the treatments. Moreover, there was a trend to higher amputation rates with the drug-coated balloon (8.8% vs. 3.6%). These balloons were withdrawn from the market because of other studies that demonstrated that the technical problem was apparently not derived from the drug, but from the design of the balloon and its production process. In the BIOLUX P-II randomized study, conducted with patients with claudication and chronic limb-threatening ischemia, a new paclitaxel-coated balloon was assessed against conventional balloons, for treatment of restenosis lesions or even native infrapopliteal arteries in patients with claudication and critical limb ischemia. Rates of the primary safety outcome, a composite of mortality from all causes, major limb amputation, thrombosis of the target lesion, and revascularization of the target vessel in 30 days, were 0% in the drug-coated balloon group vs. 8.3% in the conventional balloon group (p = 0.239). The primary performance outcome of loss of patency at 6 months and major limb amputations was more significant in the conventional balloon group at 12 month follow-up, proving that the drug-coated balloon was safe and effective in infrapopliteal lesions.^212,213^ In another study, 208 patients with severe claudication (38.6%) or chronic limb-threatening ischemia (61.4%) were analyzed retrospectively. Two-thirds of the sample had totally occluded target lesions and 17.8% of the entire patient sample had occlusion of all three infrapopliteal arteries. A total of 39 amputations were needed in 31 limbs, although 17 were minor amputations below the ankle, with just 9 (4.1%) major amputations. For the entire cohort, there was improvement by at least one Rutherford category in 130 (59.1%) limbs at 1 year or at the last assessment and 104 (80.0%) of these limbs improved by two categories, demonstrating therapeutic promise at a stage of the disease for which new treatment options are needed.^214^

Randomized controlled studies tested whether balloon angioplasty or angioplasty with conventional stents are indicated for lesions in infrapopliteal vessels, but failed to find evidence of superiority of primary stenting.^215^ A meta-analysis of 16 non-randomized clinical trials did not demonstrate advantages from primary placement of metal stents compared to balloon angioplasty alone, but did show a trend to better results with use of drug-eluting stents.^216^ However, these results were only achieved with focal lesions (< 3 cm) in segments appropriate for stenting.

Comparative studies of conventional stents and drug-eluting stents showed 1-year primary patency rates in mixed groups of patients with intermittent claudication and critical ischemia that varied from 48 to 66% in relation to balloon angioplasty and treatment without stenting. A meta-analysis of randomized studies that investigated the results of primary percutaneous revascularization with drug-eluting stents compared with balloon angioplasty alone or conventional stents in a total of 611 patients with atherosclerotic disease of the infrapopliteal arteries in 5 trials found evidence during 12-month medical follow-up showing that use of drug-eluting stents reduced the risk of target lesion restenosis and amputation, with no significant difference in mortality (Chart 21).^217^

**Chart 21:** Recommendation on the indication of infrapopliteal endovascular treatment for patients with CLTI.

**SBACV Recommendations:**	
57 Endovascular treatment is recommended as first-line treatment for patients with chronic limb-threatening ischemia and infrapopliteal vascular lesions.	1B

A comparative analysis of revascularization of infrapopliteal restenosis in 161 patients using drug-eluting stents with sirolimus showed a trend to a lower minor amputation rate, of 2.6% at 3 year follow-up vs. 12.2% with conventional stenting.^218^ Two meta-analyses of randomized studies reported similar figures at 1 year after drug-eluting stenting.^219^ Use of sirolimus-eluting stents after ineffective balloon angioplasty resulted in lower restenosis and reintervention rates compared with use of conventional stents, but did not change the amputation rate.^220^

Therefore, in focal disease (short lesions) of the infrapopliteal arteries, treatment with drug-eluting stents reduces the risk of reintervention and amputation compared with balloon angioplasty alone or conventional stenting, with no impact on mortality or Rutherford grade at 1 year follow-up.^219,221^

The influence of using drug-eluting stents on the clinical outcomes limb salvages and amputation-free survival has not yet been sufficiently explored. Additionally, their applications are always limited because of their relatively short length and the risk of crushing in areas with greater movement and flexion. Clinical data are still limited for infrapopliteal lesions and long-term follow-up results from large randomized studies are needed to better understand the relevance of endovascular techniques for clinical results. Obtaining results in terms of maintenance of patency of infrapopliteal lesions after intervention is more difficult than with lesions above the knee because of the characteristics of these lesions, including smaller diameters, slower blood flow, presence of lesions with considerable calcium build-up in the tunica media, and longer lesions,^222^ making the quest for even better results a challenge (Chart 22).

**Chart 22:** Recommendation on the use of balloon and/or drug-eluting stent.

**SBACV Recommendations:**	
58 Adequate vessel preparation is recommended for successful deployment of a drug-coated balloon or self-expandable stent.	1B
59 Drug-eluting stenting can be considered for treatment of short lesions in the femoropopliteal sector.	2B
60 Treatment of (long and complex) lesions in the infrapopliteal territory with drug-coated balloons require pre-dilation as a standard treatment recommendation.	2B

#### Open revascularization surgery

When atherosclerotic disease of the lower limbs is being investigated as the source of a patient’s complaint, the proximal vascular segment (known in practice as inflow) and the most distal segment (outflow) of the territory involved should always be assessed.

Open revascularization surgery is associated with a low, but significant, incidence of operative complications involving wounds and grafts, which can be avoided when using endovascular approaches. For example, aortoiliac disease is usually first treated using endovascular techniques (angioplasty with or without stenting), but when this is not possible, or has failed, surgical revascularization or use of hybrid techniques can be considered in selected patients with acceptable risk profiles.^223^ Infrainguinal disease can be treated with bypasses, generally originating from the common femoral artery (inflow) and ending at the popliteal vessels above or below the knee (tibial or pedal). Open revascularization procedures can be considered for occlusions of long segments that cannot be treated with endovascular techniques or local repair only. Patency is determined by the quality of the graft and the material employed and autologous veins (saphenous or others) yield the best results, with patency rates ranging from 60 to 80% at 5 years.^172,224^ Patency reduces significantly with more distal revascularizations reaching the tibial or pedal arteries and, therefore, these should only be performed to treat chronic limb-threatening ischemia and prosthetic grafts should be avoided at this level because of their low patency rates.

**Figure 1:**
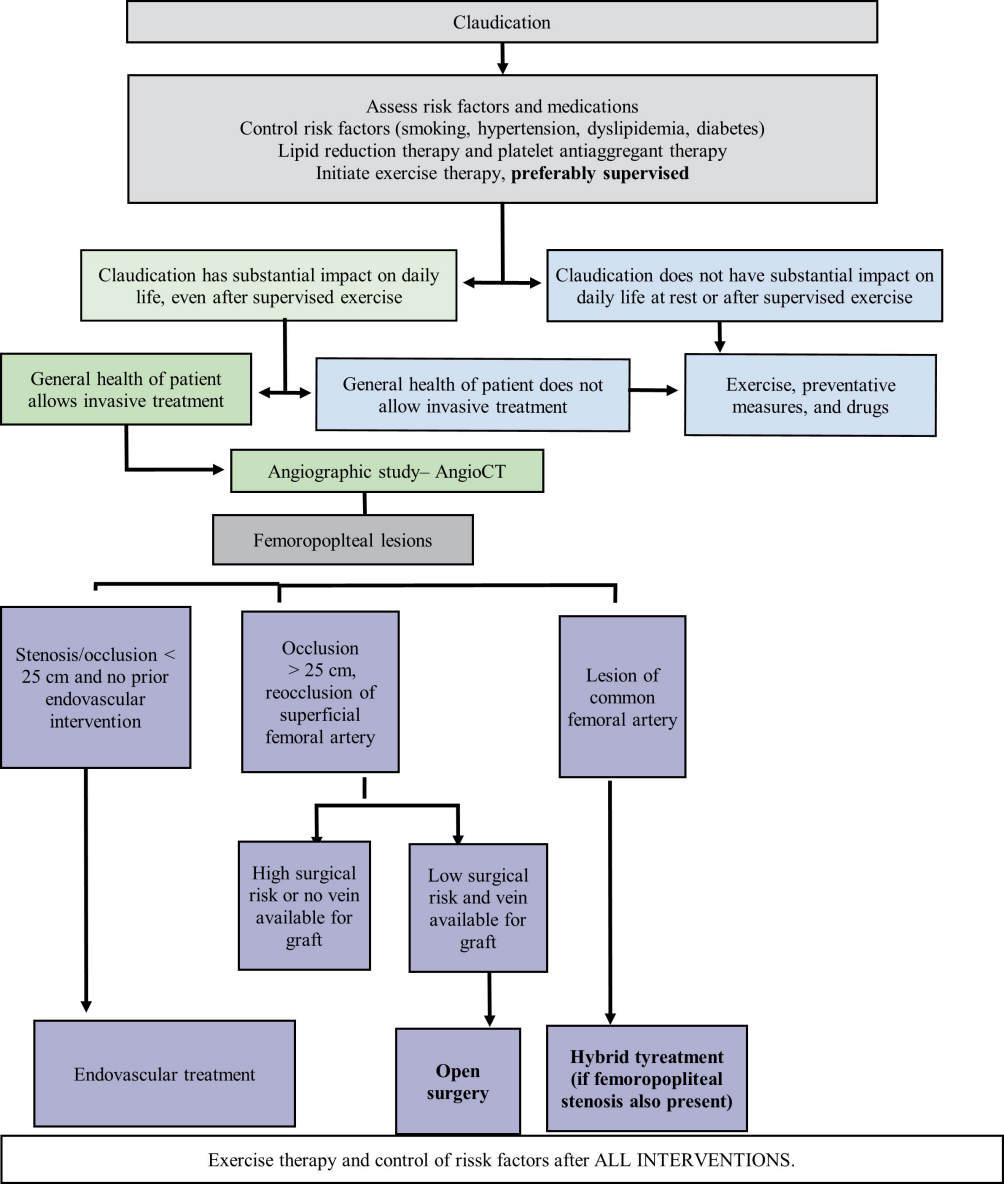
Algorithm for treatment of claudication.

Many different techniques exist for surgical, open, or endovascular management of patients with LLPAD. The decision to employ one technique rather than another must be individualized, not only in relation to the clinical parameters of the patient, but also in order to fit the infrastructure available and the expertise of the team that will perform the procedure (Figure 1).^172,179,183^ There are few RCTs of surgical versus endovascular treatment for chronic limb-threatening ischemia and differences in amputation-free survival have not been found at 1 or 5 years. However, a post-hoc analysis of the Bypass Versus Angioplasty in Severe Ischaemia of the Leg (BASIL) study suggested amputation-free survival was better with revascularization surgery.^225^ This finding confirms the superior durability of surgical revascularization compared to balloon angioplasty in patients with chronic limb-threatening ischemia. In femoropopliteal and infrapopliteal lesions for which open revascularization is under consideration, the great saphenous vein should be the first choice, since it achieves much higher rates of patency with many fewer reinterventions than artificial substitutes (PTFE and Dacron).^172,225-227^ Use of prosthetic grafts is also associated with increased incidence of infections, but in selected cases there is a possibility of using prosthetic material as a substitute for open surgery.^225,226^ It is recommended that distal fistulas and vein cuffs be used to increase patency and some studies have recommended using dual antiaggregation therapy in these patients to improve patency of prosthetic grafts (Chart 23).^104,228^

**Chart 23:** Recommendation for planning open revascularization for patients with LLPAD.

**SBACV Recommendations:**	
61 It is recommended that in planning of revascularization for LLPAD, the choice of strategy between endovascular, open, or hybrid revascularization should be made on the basis of the anatomic features of the disease, degree of ischemia, clinical condition of the patient, durability of the procedure, availability of resources, and experience of the surgeon.	1C
62 It is recommended that prosthetic synthetic material should be used in patients with infrainguinal PAD and chronic limb-threatening ischemia when endovascular treatment is not possible and no autologous veins are available as substitutes.	1C
63 It is recommended that prosthetic synthetic material should be used in combination with an interposition vein cuff in patients with below-the-knee PAD and chronic limb-threatening ischemia when endovascular treatment is not possible and no autologous veins are available as substitutes.	2C

In order to improve results when it is necessary to use a prosthetic synthetic substitute, it is recommended to combine the below-the-knee distal anastomosis with an interposition vein cuff, since its larger cross-sectional area helps with local flow dynamics, both because of formation of a coherent cortex in the conduit^229^ and because of maintenance of more constant flow through the anastomosis,^230^ with lower resistance to flow at the distal target.^231^ There is thus a redistribution of the forces of formation of intimal hyperplasia^232^ changing shear stress on the wall and reducing intimal hyperplasia stimulus.^233-235^ Another advantage is that it enables easier anastomosis, particularly between small vessels,^236^ in addition to helping to maintain flow in the event of graft thrombosis.^237^ Despite these benefits, primarily among dialysis patients, those needing more distal anastomoses (below-the-knee), and in patients with chronic limb-threatening ischemia, results of analyses of the performance of venous adjuncts have not been uniform and are even contradictory with relation to limb salvage, with many studies only confirming increased graft patency.^238-240^

**Figure 2:**
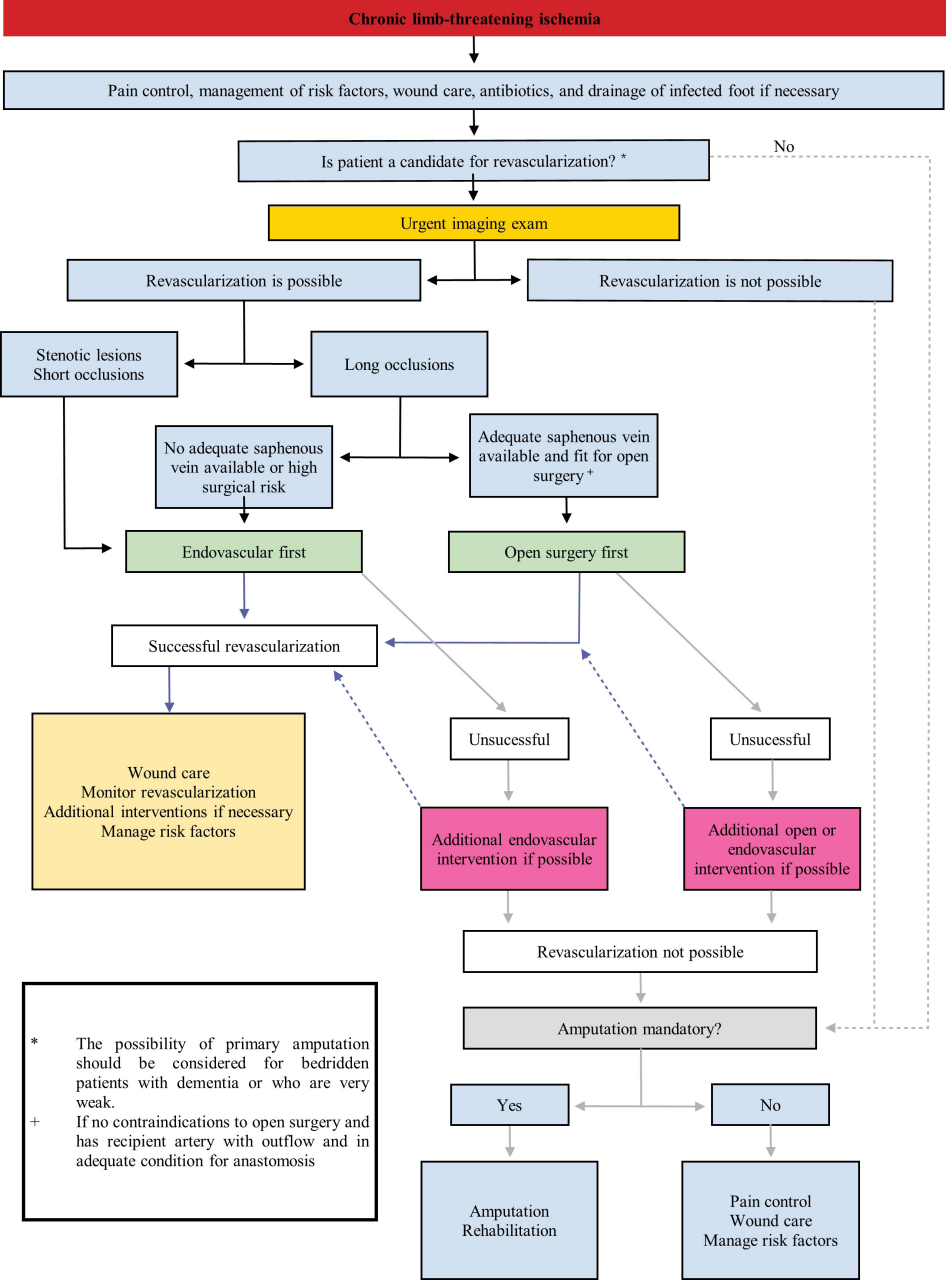
Algorithm for treatment of chronic limb-threatening ischemia.

While use of endovascular interventions has increased, open surgery remains an important treatment option in selected LLPAD patients. Endarterectomy is a technique in which the plaque is removed directly from the artery, to treat stenosis or occlusions of short segments, and the artery is then closed with a vein or angioplasty using a heterologous (bovine pericardium, for example) or synthetic patch to enlarge its diameter. This technique is often used to improve inflow or outflow, in conjunction with surgical bypass procedures. The common femoral artery is the lower limb artery most often treated with endarterectomy, which can also be performed as a stand-alone procedure. The main objective is to establish a straight arterial flow path to the deep femoral artery, since preservation of flow to this artery has a great impact on limb salvage over long term follow-up. The technique can also be used in combination at the time of revascularization to improve inflow to a bypass when necessary, contributing to improve revascularization patency rates (Figure 2).^169,225^

In general, the GLASS classification system is predictive of chronic limb-threatening ischemia and also of immediate technical failure and of patency after endovascular treatment. A meta-analysis suggests that patients at advanced GLASS stages will benefit more from bypass surgery than from endovascular treatment.^170^ Availability of an adequate graft for use in open revascularization is a decisive factor of the success of revascularization. A great saphenous vein with adequate caliber remains the substitute of choice for open surgery in this territory, demonstrating superior durability when compared with all other types of substitute: prosthetic, small saphenous vein, arm veins, spliced veins, and the SFA itself after endarterectomy (Chart 24).^241^

**Chart 24:** Recommendation for planning the open revascularization technique for patients with CLTI.

**SBACV Recommendations:**	
64 It is recommended that open endarterectomy of the common femoral artery should be performed with patch arterioplasty, with or without an extension to the deep femoral artery, in patients with CLTI with hemodynamically significant disease (> 50% stenosis) of the common and deep femoral arteries.	1B
65 It is recommended that that a bypass to the popliteal artery (when indicated) should preferably be constructed with autologous vein, rather than synthetic grafts, for treatment of intermittent claudication.	1A
66 It is recommended that that a surgical bypass to the popliteal or infrapopliteal artery should be constructed with autologous vein for chronic limb-threatening ischemia cases.	1A
67 When an above-the-knee bypass is indicated, use of prosthetic grafts should be considered, but only in the absence of any type of autologous vein.	2A
68 In patients who do not have high surgical risk, revascularization surgery is indicated for long femoropopliteal lesions (> 25 cm) when an autologous vein is available and life expectancy is > 2 years.	1A

Although endovascular treatment of CLTI has been available for more than 20 years and is an important method for treatment of patients, the clinical benefit and cost-effectiveness of using endovascular technologies are still unclear.^242,243^ To date, the BASIL and BEST-CLI studies remain the only randomized controlled studies that have compared the results of open and endovascular treatment and the idea remains that open treatment should still be considered first for patients with good life expectancy and a good autologous substitute.^98,172,183,226^

Lower limb artery reconstruction remains the cornerstone of limb salvage in chronic limb-threatening ischemia. Over the last 2 decades, progress in patient assessment and selection has resulted in a more aggressive approach with greater success, especially in extremely distal reconstructions. While patency of revascularizations and limb salvage are continuously improving, further studies are needed to assess the cost-effectiveness of infrainguinal revascularizations and the effect on patient quality of life. It is important to mention that it is necessary to recognize the limitations and the benefits of long-established open techniques and also of the minimally invasive endovascular techniques in order to clearly understand their complementarity and never to treat them as competitors.^172^

## LIST OF ABBREVIATIONS

SFA: superficial femoral artery

VKA: vitamin K antagonist

ECCN: electronic cigarette containing nicotine

INC: intermittent claudication

CV: cardiovascular

CAD: coronary artery disease

PAD: peripheral artery disease

LLPAD: lower limb peripheral artery disease

CVD: cardiovascular disease

DM: diabetes mellitus

DOAC: direct oral anticoagulants

DPP-4: selective incretin based dipeptidyl peptidase 4 inhibitors

CKD: chronic kidney disease

RCT: randomized controlled trials

AF: atrial fibrillation

RF: risk factor

GLASS: Global Limb Anatomic Staging System

GRADE: Grading of Recommendations Assessment, Development and Evaluation

SAH: systemic arterial hypertension

AMI: acute myocardial infarction

CI: confidence interval

CLTI: chronic limb-threatening ischemia

TBI: toe-brachial index

INR: international normalized ratio

ISTH: International Society on Thrombosis and Hemostasis

ABI: ankle-brachial index

MACE: composite of myocardial infarction, stroke, or cardiovascular death

MALE: major adverse limb events

OD: odds ratio

BP: arterial blood pressure

SBP: systolic arterial blood pressure

RR: relative risk

SBACV: Brazilian Society of Angiology and Vascular Surgery

SGLT 2: sodium-glucose co-transporter-2

DAT: dual antiplatelet therapy

SAT: single antiplatelet therapy

TP: tibioperoneal

TLR: target lesion revascularization

NRT: nicotine replacement therapy

DVT: deep venous thrombosis

WIfI: wound, ischemia and foot infection
