# Supplementary material for: Brazilian Society of Angiology and Vascular Surgery guidelines on peripheral artery disease
Source: J Vasc Bras. 2024 Oct 28;23:e20230059. doi: 10.1590/1677-5449.202300592 (PMC11530000; doi:10.1590/1677-5449.202300592)
Supplement: Translation

# Translation: Diretrizes sobre doença arterial periférica da Sociedade Brasileira de Angiologia e Cirurgia Vascular

**DOI:** 10.1590/1677-5449.202300591

**Authors:** Fabiano Luiz Erzinger, Afonso César Polimanti, Daniel Mendes Pinto, Gustavo Murta, Marcus Vinicius Cury, Ricardo Bernardo da Silva, Rodrigo Bruno Biagioni, Sergio Quilici Belckzac, Edwaldo Edner Joviliano, Walter Junior Boin de Araujo, Julio Cesar Peclat de Oliveira

**Affiliations:** 1 Hospital Erasto Gaertner, Serviço de Cirurgia Vascular, Curitiba, PR, Brasil.; 2 Sociedade Brasileira de Angiologia e de Cirurgia Vascular – SBACV-PR, Curitiba, PR, Brasil.; 3 Instituto da Circulação, Curitiba, PR, Brasil.; 4 Sociedade Brasileira de Angiologia e de Cirurgia Vascular – SBACV-SP, São Paulo, SP, Brasil.; 5 Sociedade Brasileira de Angiologia e de Cirurgia Vascular – SBACV-MG, Belo Horizonte, MG, Brasil.; 6 Hospital Felicio Rocho Ringgold, Cirurgia Vascular, Belo Horizonte, MG, Brasil.; 7 Rede Mater Dei de Saúde, Cirurgia Vascular, Belo Horizonte, MG, Brasil.; 8 Instituto de Assistência ao Servidor Público Estadual de São Paulo – IAMPSE, Serviço de Cirurgia Vascular e Endovascular, São Paulo, SP, Brasil.; 9 Pontifícia Universidade Católica do Paraná – PUCPR, Cirurgia Vascular, Curitiba, PR, Brasil.; 10 Santa Casa de Londrina, Cirurgia Vascular, Londrina, PR, Brasil.; 11 Sociedade Brasileira de Radiologia Intervencionista e Cirurgia Endovascular – SOBRICE, São Paulo, SP, Brasil.; 12 Instituto de Aprimoramento e Pesquisa em Angiorradiologia e Cirurgia Endovascular – IAPACE, São Paulo, SP, Brasil.; 13 Universidade de São Paulo – USP, Faculdade de Medicina de Ribeirão Preto – FMRP, Ribeirão Preto, SP, Brasil.; 14 Universidade Federal do Paraná – UFPR, Hospital das Clínicas – HC, Curitiba, PR, Brasil.; 15 Universidade Federal do Estado do Rio de Janeiro – UNIRIO, Departamento de Cirurgia Vascular, Rio de Janeiro, RJ, Brasil.

**Keywords:** doença arterial periférica, diagnóstico, tratamento, metanálise

## Abstract

Pacientes com doença arterial periférica e aterosclerose generalizada apresentam alto risco de complicações cardiovasculares e nos membros, o que afeta sua qualidade de vida e longevidade. A doença aterosclerótica das extremidades inferiores está associada à alta morbimortalidade cardiovascular, sendo necessário para sua adequada terapia realizar o tratamento dos fatores dependentes do paciente, como a modificação no estilo de vida, e dos fatores dependentes do médico, como o tratamento clínico, tratamento endovascular ou cirurgia convencional. A abordagem médica para a doença arterial periférica é multifacetada, e inclui como principais medidas a redução do nível do colesterol, a terapia antitrombótica, o controle da pressão arterial e do diabetes e a cessação do tabagismo. A adesão a esse regime pode reduzir as complicações relacionadas aos membros, como a isquemia crônica que ameaça o membro e pode levar à sua amputação, e as complicações sistêmicas da aterosclerose, como o acidente vascular cerebral e infarto do miocárdio.

## PADRONIZAÇÃO DA NOMENCLATURA

O objetivo é estabelecer uma padronização quanto à nomenclatura utilizada nesta diretriz, bem como consolidar os conceitos utilizados.

***1.* *Sucesso técnico*:** definido como o emprego com sucesso de um dispositivo, seja balão, stent ou aterótomo, de modo a restaurar a perviedade do vaso com uma estenose residual inferior a 30%. O critério angiográfico, apesar de subjetivo, é válido como determinante do sucesso técnico; contudo, principalmente em estudos envolvendo novos dispositivos, é importante ter uma análise independente realizada por um *core lab*. Entre as medidas objetivas para alcançar sucesso técnico inclui-se ultrassonografia intra-vascular e medida de gradiente translesão inferior a 10 mmHg, sendo esta última ocasionalmente empregada nas intervenções aorto-ilíacas^1,2^ .

***2.* *Sucesso hemodinâmico*:** entendido como a presença de um gradiente translesão inferior a 10 mmHg, não sendo frequente seu uso nas intervenções infrainguinais. Do ponto de vista clínico, uma medida objetiva é a realização do índice tornozelo-braço (ITB) pré e pós-operatório^3^ . Um incremento de pelo menos 0,15 no índice obtido das artérias podálicas (dorsal do pé, tibial posterior / plantar ou maleolar externa) é um indicativo de sucesso hemodinâmico. Da mesma forma, a redução desse índice superior a 0,15 é um indicativo indireto de estenose > 50% no segmento tratado^3^ . Quando disponível e aplicado, um incremento de 0,10 no índice dedo-braço também é indicativo de sucesso hemodinâmico^4^ . Quanto ao critério ultrassonográfico, o sucesso hemodinâmico é a comprovação de aumento de pelo menos 50% na velocidade de pico sistólico no segmento tratado^3^ .

***3.* *Salvamento de membro*:** definido como a preservação do membro, independentemente do *status* de óbito. Dessa forma, nas populações com alta mortalidade pós-operatória e alto sucesso técnico, eventualmente é possível deparar-se com a presença de curvas Kaplan-Meier, onde o salvamento de membro é superior à sobrevida global. Tal resultado tem feito com que esse termo venha sendo progressivamente substituído por desfecho de sobrevida livre de amputação.

***4.* *Amputação menor e maior*:** a amputação menor não deve ser entendida como um desfecho adverso após a revascularização de um membro com perda tecidual. Sua realização tem por objetivo permitir a cicatrização adequada das lesões, com intuito de preservação do membro. Nesse sentido, amputação menor é entendida como aquela em que o ato cirúrgico leva à preservação do pé, suficientemente para o indivíduo manter a deambulação sem o uso de uma prótese. De modo geral, as amputações menores são aquelas realizadas nos níveis transfalangeano ou transmetatársico. Portanto, amputações altas do pé, incluindo Syme e Chopart, são classificadas como amputações maiores. Procedimentos de revascularização com intuito de realizar amputação infrapatelar ao invés de suprapatelar também são classificados como amputação maior, e consequentemente, não são designados como “salvamento de membro”.

***5.* *Sobrevida livre de amputação*:** definida como um desfecho composto, já que para o paciente ser enquadrado nesse conceito faz-se necessário ele estar vivo e com o membro preservado. Diferentemente do salvamento do membro, se ocorre óbito no seguimento pós-operatório, o desfecho adverso é marcado como o dia do óbito, independentemente da preservação do membro submetido à intervenção. Da mesma maneira, se no seguimento uma amputação maior é necessária e o indivíduo se mantém vivo, o desfecho adverso é assinalado como o dia em que ocorreu a amputação maior.

***6.* *Perviedade*:** nas intervenções, o segmento é considerado pérvio se um dos seguintes critérios é obedecido:

- Perviedade do segmento submetido a tratamento, acessado por exame de imagem seja angiotomografia, angioressonância, angiografia digital ou ultrassonografia Doppler;
- Presença de pulso palpável a jusante ao local submetido a tratamento, comparado ao pulso ausente no pré-operatório;
- Aumento de 0,15 no ITB pós-operatório ou incremento de pelo menos 50% na velocidade de pico sistólico a jusante ao local submetido a tratamento.

Nas publicações cientificas, os critérios B e C são aceitos, mas são entendidos como critérios fracos e um tanto subjetivos. Sem dúvida, o critério acessado por exame de imagem é o melhor critério comprobatório da perviedade do segmento tratado. Apesar de objetivo, o ITB pode apresentar aumento superior a 0,15 e não refletir necessariamente perviedade do segmento. Tal situação é particularmente identificada nas intervenções endovasculares de múltiplos segmentos, onde é possível a manutenção da elevação do índice apesar da oclusão de um dos segmentos tratados.

***7.* *Perviedades primária, primária assistida e secundária*** : perviedade primária refere-se à manutenção ininterrupta do fluxo através de um segmento submetido à intervenção, ou o intervalo de tempo entre a intervenção inicial e um procedimento adjuvante que vise a manter a perviedade. Nesse sentido, pacientes que apresentam estenose superior a 50% no segmento tratado, mas que não são submetidos à reintervenção, são enquadrados no conceito de perviedade primária. Em algumas publicações, o pesquisador define como perviedade primária a perviedade ininterrupta, além da presença de estenose < 50% no segmento tratado. Esse conceito também é aceito, porém, de modo geral, envolve a menção desse critério na metodologia do trabalho.

Quando uma intervenção é realizada com intuito de corrigir a reestenose pós-intervenção, assegurando a manutenção da perviedade primária, ou seja, sem que tenha ocorrido oclusão, o conceito aplicável é perviedade primária assistida. Quanto à perviedade secundária, trata-se da perviedade obtida através de procedimentos secundários após a oclusão do segmento originalmente tratado. Sendo assim, destacam-se como procedimentos de perviedade secundária trombectomias farmacológicas, mecânicas ou fármaco-mecânicas das oclusões pós-intervenção.

A depender das definições dos desfechos estabelecidos pelo pesquisador, cabe ressaltar que a realização de recanalizações nos locais submetidos à angioplastia e ocluídos, sem trombectomias adjuvantes, é melhor entendida como nova intervenção ou *redo*, ao invés de procedimentos de perviedade secundária.

***8. Lesão alvo*:** diz respeito a qualquer tipo de lesão tratada ou submetida à tentativa de tratamento. De modo geral, a análise dos segmentos tratados é realizada de forma individualizada, ou seja, se um paciente recebe um stent na artéria femoral superficial (AFS) e realiza concomitantemente uma angioplastia de artéria tibial anterior, os dados de sucesso técnico e perviedade devem ser idealmente reportados para cada um dos vasos tratados, e não de forma global. Por definição, a lesão alvo inclui o segmento de 10 mm proximal e 10 mm distal ao local tratado.

***9*. *Revascularização da artéria alvo* (TLR, do inglês, *target lesion revascularization)*:** o conceito foi originalmente idealizado para as intervenções endovasculares coronarianas, especificamente como método de reportar as reestenoses. Conceitualmente, diz respeito a qualquer tipo de reintervenção realizada no segmento originalmente tratado, incluindo os 10 mm proximais e distais à intervenção inicial. Se o local originalmente tratado se encontra ocluído, e, por exemplo, uma derivação arterial é realizada com anastomose abaixo desse local, tal situação é entendida como revascularização de artéria alvo. De modo geral, os desfechos relacionados a esse conceito são reportados como sobrevida livre de revascularização da artéria alvo, através de curvas Kaplan-Meier.

Considerando o exposto, algumas diretrizes, como a da Society of Vascular Surgery (SVS) não recomendam a utilização do TLR como marcador primário de sucesso das intervenções de revascularização dos membros inferiores (MMII)^2^ . Muitas vezes, a avaliação pelo TLR relaciona-se com a realização de reintervenções não direcionadas pela clínica do paciente, mas sim pela presença de reestenose > 50%. Frequentemente pacientes com reestenose com lesões periféricas já cicatrizadas não são submetidos à reintervenção. Dessa forma, quando as lesões periféricas se encontram não cicatrizadas e uma reintervenção é realizada em um local de reestenose > 50%, foi concebido o conceito do *clinical driven* TLR, ou seja, revascularização da artéria alvo direcionada pela clínica do paciente^1^ .

***10.* *Desfechos adversos maiores*:** muitos trabalhos utilizam o recurso da adição de desfechos compostos com intuito de demonstrar a superioridade de uma técnica em relação à outra, ou mesmo com o objetivo de assegurar a eficácia / segurança de um determinado procedimento. É frequente o pesquisador reportar desfechos compostos, os quais incluem: sobrevida livre de evento maior, sobrevida livre de evento maior no membro etc. Não há uma regra em relação à definição desses eventos adversos maiores, os quais frequentemente são definidos pelo próprio pesquisador na metodologia do trabalho. Dessa forma, eventos adversos maiores podem ser: necessidade de reintervenção e/ou amputação maior e/ou óbito, por exemplo.

## OBJETIVO

O objetivo da presente diretriz é fornecer recomendações de cuidados abrangentes, otimizadas e baseadas em evidências, para pacientes com DAP dos MMII, e oferecer recomendações de prática clínica confiáveis por meio da transparência e divulgação por aqueles que participam do processo de desenvolvimento, onde a indústria não tenha influência direta sobre o conteúdo clínico e suas recomendações, - o que é fundamental para um documento confiável e independente. A diretriz refere-se a adultos com distúrbios da circulação arterial periférica assintomáticos ou sintomáticos devido à aterosclerose, e não se aplica, portanto, para crianças. As estratégias de tratamento para causas não ateromatosas de processos de oclusão arterial periférica (vasculite, dissecção, arterite de células gigantes, displasia fibromuscular, estenoses radiogênicas e síndromes de aprisionamento) devem ser diferenciadas de estenoses/oclusões ateroscleróticas e não são foco de atenção desta diretriz, assim como não o são as situações de emergências, como as oclusões arteriais aguda e lesões traumáticas.

A diretriz destina-se a auxiliar a equipe médica e os pacientes na tomada de decisões sobre as melhores medidas diagnósticas e terapêuticas para pacientes com DAP, e auxiliar nas rotas de ação e decisão, podendo ser servir como fonte de informações atualizadas para instituições de saúde pública e políticas governamentais As diretrizes estabelecidas pelas sociedades médicas científicas não são juridicamente vinculativas para os médicos e, portanto, não podem determinar responsabilidade ou isentar os médicos de responsabilidade. O que legalmente constitui um padrão médico no tratamento de um determinado paciente só pode ser determinado na avalição individual do mesmo; portanto, esta diretriz não isenta os médicos de sua obrigação de cuidar individualmente de seus pacientes, avaliando a situação geral de cada um deles.

## INTRODUÇÃO

A DAP compreende um grupo diverso de distúrbios que levam à estenose progressiva, oclusão ou dilatação aneurismática da aorta e seus ramos não coronários, incluindo os ramos carotídeos das extremidades superiores e ramos viscerais e arteriais das extremidades inferiores^5,6^ . A prevalência da DAP é de aproximadamente 12% na população adulta, afetando ligeiramente mais homens do que mulheres, acometendo mais de 200 milhões de pessoas em todo o mundo, dependendo da idade^7-9^ . É frequentemente assintomática, subdiagnosticada e subtratada, sendo uma das principais causas de morbidade e mortalidade cardiovascular (CV) e cerebrovascular. A ativação da coagulação e a estimulação endotelial estão significativamente aumentadas nesses pacientes, com ativação plaquetária elevada, níveis alterados de fibrinogênio, geração de trombina e fibrina^10^ , sendo que tais situações explicam a relação da gravidade representada pela doença arterial e as dificuldades do seu tratamento e acompanhamento^11^ . Principalmente aqueles pacientes com isquemia crônica que ameaça o membro (ICAM) apresentam as vias pró-coagulantes, anticoagulantes e fibrinolíticas desreguladas, com diminuição dos anticoagulantes naturais (proteínas C e S) e dos fatores de coagulação FIX, FXI e FXII^12^ , justificando a presença frequente da trombose arterial^13^ . No estudo de Framingham, 75% dos pacientes com DAP morreram de eventos cardiovasculares, observando-se que em pacientes com claudicação intermitente (CI) a mortalidade foi duas a três vezes maior^14^ . A apresentação mais frequente da DAP é a doença arterial periférica dos membros inferiores (DAPMI), com sintomas de cãibras, cansaço, dormência ou fraqueza nas pernas, quadris, coxas ou músculos da panturrilha durante certas atividades. Aproximadamente 50% desses pacientes apresentam sintomas como CI ou outros, que podem evoluir para isquemia aguda do membro^15^ . No entanto, a doença é muitas vezes subdiagnosticada pela ausência de sintomas isquêmicos relacionados às pernas, ou mesmo sintomas atípicos, ou então pela característica lentamente progressiva da doença, sendo muitas vezes confundida pelo paciente com falta de condicionamento físico. O risco de DAPMI aumenta quando há fatores de risco cardiovascular associados, como hipertensão, dislipidemia, tabagismo e diabetes^16-18^ , justificando a frequência maior nos pacientes com DAPMI também das doenças cardíacas, incluindo doença arterial coronariana (DAC), insuficiência cardíaca e fibrilação atrial (FA). Isso exige uma abordagem multidisciplinar, com importantes implicações terapêuticas e prognósticas para o adequado tratamento. Pacientes com DAPMI, portanto, têm alto risco de eventos cardiovasculares adversos graves, como acidente vascular cerebral (AVC) não fatal, infarto do miocárdio não fatal e morte por razões cardiovasculares (MACE) a longo prazo, e alto risco de sofrerem eventos adversos graves nos membros (MALE), como isquemia grave do membro, gangrena, comprometimento funcional e amputação com mortalidade de aproximadamente 50% em 1 ano^19^ . O tratamento e controle da DAPMI inclui redução do colesterol, controle da pressão arterial (PA) e do nível de glicose, terapia com exercícios físicos e cessação do tabagismo. A terapia apropriada pode ser inicialmente farmacológica, usando-se antiplaquetários e anticoagulantes orais, para posteriormente serem indicadas terapias cirúrgicas^20^ .

## METODOLOGIA

O tema e escopo foram escolhidos pela Diretoria da Sociedade Brasileira de Angiologia e Cirurgia Vascular (SBACV), dando total autonomia aos coordenadores responsáveis em organizar a diretriz. Os coordenadores foram designados com base nas suas pesquisas e experiência clínica, revisando e atualizando as principais diretrizes já publicadas em DAP por meio de uma varredura direcionada principalmente para revisões sistemáticas e metanálises, no período de 2015 a 2022. A pesquisa foi realizada na PubMed, através das palavras-chave “doença arterial periférica”, “diagnóstico”, “tratamento” e “metanálise”. O processo de desenvolvimento do consenso ocorreu por meio de comunicações eletrônicas confidenciais, individuais ou em duplas, dos membros do grupo de estudo, para evitar vieses de experiências pessoais. Depois de cada seção concluída, os organizadores revisaram as recomendações, e seus comentários individuais foram submetidos à aprovação dos demais. Quando mais de 2/3 dos membros concordaram, foi confirmada sua correção e adequação, e quando não houve concordância, a recomendação foi novamente revisada. Foram excluídas as doenças da aorta e das artérias ilíacas, as doenças não ateroscleróticas dos membros inferiores e as emergências (trauma e oclusão arterial aguda), tendo somente a população adulta como referência. Foi utilizado o sistema GRADE (Grading of Recommendations Assessment, Development and Evaluation), por se tratar de uma ferramenta desenvolvida por um grupo colaborativo de pesquisadores, visando à criação de um sistema universal, transparente e sensível para determinar de forma prática a qualidade da evidência e a força das recomendações. Esse sistema é utilizado atualmente pela Organização Mundial da Saúde, o National Institute for Health and Clinical Excellence e o Centers for Disease Control and Prevention^21^ . Uma recomendação forte (Grau 1) significa que os desenvolvedores da diretriz estão confiantes quanto ao equilíbrio entre análise de benefícios e malefícios, e que essa recomendação deve ser aplicada na maioria dos pacientes. Uma recomendação condicional (Grau 2) implica que há menos certeza entre vantagens e desvantagens sobre uma conduta. O nível de evidência para cada recomendação é considerado de alta qualidade (A), moderada qualidade (B) ou baixa qualidade (C), tendo sido também enquadrada como de muito baixa qualidade (C), a exemplo utilizado pelo UpToDate. Após a revisão final das recomendações e aprovação pelo grupo que organizou a diretriz, essas foram compiladas em um documento e enviadas à Diretoria Científica da SBACV nacional para a revisão final do documento e sua aprovação antes do envio para publicação e divulgação.

## DIAGNÓSTICO DA DAPMI

A DAPMI é prevalente na população com 50 anos ou mais, que apresenta sintomas nos membros inferiores sugestivos de claudicação. No entanto, tal sintoma nem sempre está presente de forma clara, sendo importante a realização do diagnóstico diferencial de outras causas de dores nas pernas (Tabela 1), começando com a história clínica completa e exame físico focado nos sinais e sintomas mais relevantes. O Questionário de Claudicação de Edimburgo (Tabela 2) é um questionário (validado para DAPMI) utilizado nos estudos epidemiológicos, que pode auxiliar na investigação da claudicação dos membros inferiores (MMII). Foi testado em 300 indivíduos com mais de 55 anos, apresentando sensibilidade de 91,3% (intervalo de confiança [IC] de 95%, 88,1-94,5%) e especificidade de 99,3% (IC de 95% 98,9-100%)^23^ , também utilizado em estudo no Brasil com similar resultado de sensibilidade e especificidade^22^ . Apenas 5 a 10% dos pacientes com DAPMI apresentaram sintomas clássicos de CI^24^; outros pacientes apresentaram desconforto inespecífico nas costas, nádegas ou pernas; e alguns pacientes poderiam ser assintomáticos. Tipicamente, pacientes com claudicação de origem vascular apresentaram cãibras, dor muscular (panturrilha ou nádega) ao caminhar uma distância definida, tendo que parar para conseguir continuar sua caminhada. Isso difere da isquemia crônica que ameaça o membro (ICAM), antes chamada de isquemia crítica, que provoca dor mesmo durante o repouso, ou com gangrena ou ainda ulceração na extremidade inferior, identificando o paciente com um quadro mais grave da mesma doença.

**Tabela 1:** Diferentes causas de dor nas pernas.

Origem Vascular	Doença arterial periférica, isquemia crônica que ameaça o membro, insuficiência venosa crônica, trombose venosa profunda, doença arterial não aterosclerótica (exemplo: síndrome de aprisionamento da artéria poplítea).
Origem Neurogênica	Estenose do canal medular, neuropatia periférica, radiculopatias, espondilolistese.
Origem Musculoesquelética	Artrite do quadril ou joelho, cisto de Baker sintomático, síndrome compartimental de esforço crônico, fratura por estresse, espasmos musculares ou cãibras.
Outras	Síndrome das pernas inquietas, vasculites, doenças oncológicas e seus tratamentos.

**Tabela 2:** Questionário de Edinburgh para claudicação (versão em português)^22^ .

1. Você sente dor ou desconforto na(s) perna(s) quando caminha?
Sim
Não
2. Essa dor começa quando você está parado ou sentado?
Sim
Não
3. Você tem essa dor ao subir uma ladeira ou andar rápido ou correr?
Sim
Não
4. Você tem essa dor quando anda no seu ritmo normal, no plano?
Sim
Não
5. O que acontece se você para?
Geralmente continua por mais de 10 minutos?
Geralmente desaparece em 10 minutos ou menos?
6. Onde você sente essa dor ou desconforto?
(Há uma figura representando os MMII para o paciente anotar o local)

Características clássicas da claudicação incluem:

1. Dor muscular, tipicamente envolvendo os músculos da panturrilha ou um grupo muscular, distal a uma estenose ou oclusão arterial e frequentemente descrita como cãibra;
2. Dor que se desenvolve apenas quando o músculo é exercitado, com caminhadas ou outras atividades físicas;
3. Dor que desaparece geralmente dentro de 10 minutos após a interrupção do exercício ou repouso.

Após verificar a história clínica completa, os médicos devem realizar um exame físico vascular periférico focado, sendo necessário para confirmação do diagnóstico de DAPMI a realização de testes específicos complementares, considerando as diferentes causas para dor nas pernas (Tabela 1)^24^ .

Entre os diversos testes complementares, o mais utilizado é o índice tornozelo-braço (ITB), sendo um teste barato e não invasivo que envolve a medição da pressão arterial sistólica (PAS) no braço (sobre a artéria braquial) e tornozelo (sobre a artéria pediosa dorsal ou artéria tibial posterior) enquanto o paciente está em decúbito dorsal. Através de um dispositivo Doppler de onda contínua obtém-se o ITB, aferindo o maior valor da pressão sistólica no tornozelo bilateral e dividindo o valor obtido pela maior pressão aferida no braço direito, e depois no esquerdo, respectivamente – quando o ITB for menor que 0,9, sugere DAPMI^25,26^ . A incidência de DAPMI varia de acordo com a prevalência de fatores de risco para a doença, como tabagismo, hipertensão, hipercolesterolemia e diabetes melito^22^ . Muitos desses pacientes também possuem calcinose arterial média, uma situação importante, mais prevalente em pacientes com diabetes, doença renal crônica (DRC) e de idade avançada. Tais situações tornam as artérias pouco compressíveis^27^ , podendo falsamente normalizar ou elevar artificialmente o ITB para um valor superior a 1,4, o que torna o teste menos confiável. Pela frequência menor de acometimento das artérias distais da doença aterosclerótica, o índice hálux-braço (IHB) pode ser calculado com aferição da PA do braço e do hálux, dividindo-se essas pressões para estabelecer uma medida de IHB para cada perna. ITB e IHB são os testes mais estudados para o diagnóstico de DAPMI, porém nos estudos existe uma falta de precisão quanto às características das populações estudadas, com grande variabilidade dos sintomas e fatores de risco dificultando a sua acurácia (por exemplo, sensibilidade de 45 a 100% e especificidade de 16 a 100% para IHB)^26^ . Há também falta de consistência em relação ao valor e na forma de realizar o IHB para estabelecer o diagnóstico para DAPMI, sendo o valor abaixo de 0,60 o mais comumente aceito entre os estudos.

### Teste de esteira

Testes de exercício em esteira são recomendados para fornecer evidência objetiva da magnitude da limitação funcional da claudicação e para medir a resposta à terapia que está sendo realizada^28^ . Recomenda-se utilizar um protocolo de exercício padronizado, com carga fixa ou progressiva. Um estudo de metanálise apontou como protocolo mais confiável o de aumento graduado e a distância absoluta de claudicação. O protocolo de carga fixa pode ser recomendado na falta de esteira ajustável, com uma inclinação positiva de 12°, a 3,2 km/h^29^ . Quando o teste na esteira é interrompido pelo fato de o paciente não conseguir mais andar, o resultado é definido como a distância máxima de caminhada, tornando-se útil para auxiliar na diferenciação se a dor do membro é de origem isquêmica ou não. Uma diminuição da PAS aferida no tornozelo após exercício > 30 mmHg ou uma diminuição do ITB após exercício > 20% são diagnósticos de doença arterial dos MMII^25^ (Quadro 1).

**Quadro 1:** Recomendação no uso de ITB na avaliação da DAP.

**Recomendações da SBACV**	
1 Sugere-se usar o ITB para triagem de adultos assintomáticos, com mais de 50 anos, que tenham fatores de risco para DAP (como tabagismo ou diabetes).	2C
2 Recomenda-se o uso de estudo ITB e ou (IHB) para confirmar o diagnóstico de DAP em pacientes com sintomas de DAP.	1B
3 Sugerimos o uso de um índice hálux-braço como adjuvante para pacientes com sintomas de DAP com artérias calcificadas para confirmar o diagnóstico de DAP.	1C

## MANEJO DA DAPMI

### Tabagismo

O valor da cessação do tabagismo é considerado alto não apenas devido ao seu efeito sobre a doença vascular, mas também pelo profundo efeito na prevenção de muitos cânceres e na doença pulmonar obstrutiva crônica. O tabagismo é considerado um dos principais fatores de risco cardiovascular. Está associado ao desenvolvimento e à progressão da DAP e de seus eventos adversos maiores nos membros inferiores, além de complicações como AVC, infarto do miocárdio e morte CV. Portanto, a cessação do tabagismo é fundamental para auxiliar na prevenção e redução de tais danos^30,31^ (Quadro 2).

**Quadro 2:** Recomendação na importância da cessação do tabagismo na DAPMI.

**Recomendações da SBACV:**	
4 Recomenda-se a cessação do tabagismo para prevenir DAPMI e para prevenir MACE e MALE em pacientes com DAPMI.	1B

Na busca para cessar o tabagismo, além do aconselhamento comportamental, a terapia farmacológica deve ser considerada, desde a terapia de reposição de nicotina (TRN), como chicletes e adesivos, até bupropiona e vareniclina. Os cigarros eletrônicos contendo nicotina (CEN) também devem ser considerados, levando-se em conta que não há evidência robusta que comprove seu resultado no que concerne à diminuição do risco cardiovascular e segurança pulmonar^32,33^ . A terapia comportamental com apoio da farmacoterapia aumenta a taxa de abandono em média de 20% nos primeiros 6 meses. Quando comparada aos pacientes que não recebem terapia, o abandono ocorre em 17%^34^ , com dados de eficácia muito variáveis na literatura, como mostra a revisão da Cochrane. As terapias medicamentosas são poucas e com limitados dados na literatura, principalmente quanto ao uso da bupropiona e vareniclina. Uma combinação de bupropiona com TRN ou com vareniclina aparentemente melhora a taxa de abandono, porém de maneira não significativa. Em uma metanálise com 267 estudos com 101.804 participantes, a vareniclina e a combinação de TRN (ou seja, combinando dois tipos de TRN, como adesivos, comprimidos, *sprays*, pastilhas e inaladores) *versus* placebo mostrou-se a mais eficaz de todas as intervenções medicamentosas, aceitando-se a náusea como efeito colateral frequente^35^ . Os CEN têm sido defendidos para reduzir o vício de fumar. Uma revisão recente da Cochrane, que comparou CEN com a TRN, mostrou efeito positivo no abandono, com um risco relativo (RR) de 1,53, com IC de 95%, 1,21-1,93, resultando em três desistentes adicionais por 100 após 6 meses^34^ .

As intervenções para parar de fumar, por meio de aconselhamento comportamental com ou sem TRN ou um programa de intervenção comunitária que promova a redução do tabagismo, sugeriram que essas devem durar no mínimo 6 meses^36^ para aumentar a chance de parar de fumar^37^ ; mas, para confirmar tais dados, ainda se necessita de mais estudos nesta área. Mesmo assim, o aconselhamento intensivo traz benefícios na cessação do tabagismo, sugerindo ser esse uma importante e eficaz estratégia de cessação do tabagismo para pacientes com DAP^38^ (Quadro 3).

**Quadro 3:** Recomendação nas intervenções para cessar o tabagismo.

**Recomendações da SBACV:**	
5 Recomenda-se intervenções para parar de fumar, que vão desde aconselhamento intensivo, TRN, bupropiona até vareniclina.	1A

### Diabetes

Pacientes com diabetes e DAPMI concomitantes têm um aumento na mortalidade de três a quatro vezes e uma taxa de amputação cinco vezes maior do que nos pacientes sem diabetes^39^ . A escolha de agentes anti-hiperglicêmicos em pacientes com DAPMI deve ser individualizada, sendo uma das metas de tratamento a redução do risco CV e redução dos eventos adversos graves nos membros^40^ . Infelizmente, muito poucos medicamentos hipoglicemiantes foram estudados em pacientes com DAPMI, embora alguns tenham se mostrado mais promissores do que outros. A metformina é o antidiabético oral de primeira linha em diabéticos com DAPMI, e, embora os dados sejam escassos a esse respeito, estudos demonstram efeito positivo na sobrevida CV, mas não na prevenção de amputação^41,42^ . No estudo de avaliação cardiovascular da canagliflozina (Canvas), esta foi associada a um risco aumentado de amputação entre pacientes com diabetes, o que não foi observado com outros inibidores de SGLT-2, como empagliflozina e dapagliflozina. Duas grandes metanálises não relataram risco geral aumentado significativo de amputação com inibidores de SGLT-2 como classe, mas, sim, um problema especifico no uso da canagliflozina, não sendo recomendada para pacientes diabéticos com DAPMI associado^43,44^ . Da mesma forma, estudos utilizando os inibidores de SGLT-2, com desfechos para DAPMI, observaram que as medicações mais associadas a desfecho positivo foram a empaglifozina e a liraglutida, as quais demonstraram reduzir as amputações; portanto, essas substâncias devem ser consideradas em adição à metformina em pacientes com diabetes com DAPMI previamente conhecida^45,46^ . Outro exemplo são os medicamentos inibidores seletivos baseados em incretina de dipeptidil peptidase 4 (DPP-4) usados no diabetes, que não demonstraram redução de MACE ou MALE em pacientes com DAPMI. No entanto, quando combinados à metformina, ocorreu uma redução de 16% no desenvolvimento de DAP em diabéticos tipo 2, com redução de 35% nas amputações^47,48^ . O objetivo para a maioria dos adultos com DM é manter uma glicose-nível de hemoglobina A1c tardia < 7%^49-52^ . No entanto, metas menos rigorosas (por exemplo, nível de hemoglobina A1c < 8%) podem ser apropriadas para indivíduos com complicações vasculares ou expectativa de vida limitada^53^ . Pacientes com DM tipo 2 com função renal anormal tratados com metformina podem ter maior risco de nefropatia induzida por contraste e acidose láctica. Considerando que o assunto é objeto de debate contínuo, é razoável suspender a metformina por 24 a 48 horas antes e após a administração de um agente de contraste iodado^54,55^ (Quadro 4).

**Quadro 4:** Recomendação nos cuidados para os pacientes diabéticos relacionados a DAP.

**Recomendações da SBACV:**	
6 Recomenda-se que pacientes com diabetes sejam rastreados para DAP.	1B
7 Recomenda-se o controle diabético adequado para pacientes com DAP.	1B
8 Recomenda-se que todos os pacientes com DAP sejam tratados efetivamente no caso de um diagnóstico comprovado de diabetes. Em diabéticos tipo 2, além da metformina, deve-se considerar o uso de empaglifozina e/ou liraglutida.	1B
9 Sugere-se que pacientes com DAP e diabetes possam se beneficiar do uso de um inibidor de DPP-4.	2C
10 O controle do DM tipo 2 em pacientes com isquemia crônica com risco ao membro deverá ter como meta uma hemoglobina A1c < 7%.	2B
11 Recomenda-se que pacientes com DAP e diabetes tipo 2 recebam um inibidor de SGLT-2 *versus* o controle diabético usual, devido à redução de MACE, sem qualquer risco no aumento da taxa de amputação.	1C

### Dislipidemia

A redução agressiva de lipídios com estatinas é necessária para reduzir os principais eventos adversos cardiovasculares e cerebrovasculares, e, consequentemente, diminuir a mortalidade geral e cardiovascular. Há fortes evidências para apoiar essa intervenção com o objetivo de evitar o MALE, mas na comprovação de melhores resultados no aumento do tempo de caminhada sem dor as evidências não são tão evidentes^56-59^ . Concentrações elevadas de colesterol total, aumento dos níveis de colesterol de lipoproteína de baixa densidade (LDL), triglicerídeos e lipoproteína e a diminuição dos níveis de lipoproteína de alta densidade (HDL) são fatores de risco independentes para o desenvolvimento de DAP^60^ . O benefício no uso de estatinas no controle do colesterol torna-se tão importante para a prevenção de eventos vasculares e na mortalidade geral, que as análises Cochrane endossaram os benefícios, custo-benefício e melhoria da qualidade de vida associados às estatinas, mesmo em pacientes de baixo risco, apesar dos efeitos colaterais indesejáveis^61^ . Os benefícios da redução de lipídios com estatinas e outros agentes redutores de lipídios na DAP são inquestionáveis. Há evidências menos robustas que apoiam a ideia de que a redução dos lipídios também pode melhorar a distância para caminhar sem dor (claudicação)^62^ , assim como as chances de amputação e aumento da perviedade de pontes^63,64^ . No registro REACH, pacientes com DAP conhecida, nos quais foi aplicada a terapia com estatina, mostraram redução da necessidade de revascularização periférica, de 21,7 para 18,2%, e na taxa de amputação de 5,6 para 3,8%, no período de 4 anos^65^ . Em pacientes com claudicação, estudos com diferentes doses de atorvastatina ou sinvastatina mostraram melhora significativa da distância percorrida livre de dor ou caminhada absoluta em comparação com placebo, em 3, 6 ou 12 meses. Embora alguns dos estudos tenham examinado apenas pequenas coortes de pacientes, eles mostram um efeito homogêneo e reprodutível independentemente do período de observação para ambas as substâncias^56,66^ . Em pacientes com isquemia crítica de extremidade após cirurgia de ponte venosa, as estatinas melhoraram significativamente a sobrevida após um ano no estudo PREVENT III (Projeto de engenharia de enxerto de veia Ex-Vivo via transfecção III)^67^ . Pacientes com DAP devem ter seu colesterol de lipoproteína de baixa densidade sérica (LDL-C) reduzido para < 70 mg/dL ou diminuído em >50% se o nível inicial de LDL-C estiver entre 70 e 135 mg/dL, para redução na mortalidade e eventos CV^57,68,69^ . Considerações adicionais sobre terapia adicional, como ezetimiba e evolocumab^70^ , podem ser necessárias em pacientes com dificuldade de redução dos níveis de lipídios e que tenham um risco de novos eventos CV. Portanto, pacientes com DAP são considerados pacientes de alto risco e devem receber tratamento “intensivo” com estatina, com o objetivo de reduzir o risco cardiovascular de forma eficaz e tolerável. A assistência médica deve focar no acompanhamento desses pacientes, uma vez que a não adesão tem se mostrado um problema considerável na prática diária (Quadro 5).

**Quadro 5:** Recomendação no uso de estatinas para os pacientes com DAP.

**Recomendações da SBACV:**	
12 As estatinas são recomendadas para prevenção secundária em todos os pacientes com DAP que toleram tal medicação.	1A
13 Além da prevenção geral, as estatinas são indicadas para melhorar a distância de caminhada.	1B
14 Recomenda-se que em pacientes com DAP, o LDL seja reduzido para 70 mg/dL ou reduzido em 50% se os valores pré-tratamento forem de 70-135 mg/dL.	1A

### Hipertensão arterial sistêmica

A hipertensão arterial sistêmica (HAS), de maneira geral, está associada a um aumento da prevalência de DAP^71^ , sendo que com o aumento da idade torna-se ainda maior a importância da contribuição da PA elevada para incidência de DAPMI. Um grande estudo populacional, com 4,2 milhões de adultos, demonstrou que homens de 40 a 79 anos, com HAS, quando apresentavam um acréscimo de 20 mmHg na PAS, tiveram risco aumentado de 63% para desenvolver DAPMI^72^ . Um dos objetivos específicos do tratamento do paciente hipertenso é obter o controle pressórico alcançando a meta de PA previamente estabelecida, a qual deve ser definida individualmente, sempre considerando a idade e presença de doença cardiovascular (DCV) ou de seus fatores de risco. Na diretriz brasileira de hipertensão arterial^73^ , o hipertenso com DAC deve ter como meta terapêutica uma PA <130/80 mmHg, enquanto a PA diastólica deve ser mantida com valores acima de 70 mmHg. Para hipertensos com insuficiência cardíaca ou episódio prévio de acidente vascular encefálico e DRC, e para diabéticos, o tratamento anti-hipertensivo deve ser titulado até alcançar a meta PA <130/80 mmHg. Devido à concomitância de doença DAC e idade avançada, comum em tais situações, e também nos pacientes diabéticos, evita-se a redução da PA abaixo de 120/70 mmHg. Hipertensos com DRC sempre devem ser monitorados quanto a eventos adversos, especialmente quando ocorre redução na função renal e alterações eletrolíticas^74^ . A terapia anti-hipertensiva reduz inequivocamente os eventos CV e a mortalidade. Uma PAS abaixo de 120 mmHg não é desejável, uma vez que pode aumentar o risco de eventos coronarianos agudos^75,76^ (Quadro 6).

**Quadro 6:** Recomendação nos cuidados da hipertensão arterial para os pacientes com DAPMI.

**Recomendações da SBACV:**
15 Em pacientes com DAPMI e hipertensão arterial, a pressão arterial deve ser tratada para reduzir eventos cardiovasculares.	1A
16 Nos hipertensos de risco CV baixo ou moderado, a meta de tratamento é alcançar valores inferiores de 140/90 mmHg, e com risco CV alto, PA < 130/80 mmHg.	1B

### Drogas antitrombóticas

#### No paciente com DAPMI assintomática

Pacientes com ITB baixo, mas sem sintomas clínicos nos membros ou sem intervenção vascular prévia, são considerados portadores de DAPMI assintomática, e há dificuldade de encontrar benefício comprovado para apoiar o uso de aspirina (AAS) nesses pacientes assintomáticos ou sem DAP em outros territórios. Já em pacientes com outra doença aterosclerótica clínica (por exemplo, DAC), há risco aumentado de eventos cardiovasculares, necessitando uma abordagem antitrombótica intensificada^77^ . No entanto, pacientes com DAP assintomático nos MMII, com índice tornozelo-braquial abaixo de 0,9, têm risco aumentado para MACE e MALE^78,79^ , e os estudos até o momento não conseguiram mostrar benefício do uso da aspirina em longo prazo nesse perfil de paciente^80^ . Por exemplo, um estudo populacional randomizado, duplo-cego, abrangendo um total de 28.980 residentes escoceses, não encontrou distúrbios cardiovasculares clinicamente evidentes entre 3.500 indivíduos com ITB abaixo de 0,95. Após um período de tratamento e acompanhamento de 8,2 anos, a administração de AAS 100 mg/dia por via oral não produziu nenhuma diferença na taxa de eventos cardiovasculares em comparação com o placebo^81^ . Os pacientes com diabetes e com DAP assintomática, recebendo 100 mg diários de AAS, não mostraram taxas reduzidas de eventos cardiovasculares (IM letal e não letal, AVC, mortalidade cardiovascular) nem taxas reduzidas de amputações maiores em comparação com pacientes tratados com placebo^82,83^ . No entanto, deve-se tomar cuidado na interpretação do que é um paciente assintomático, pois sintomas atípicos são comuns para DAPMI. Para a adequada classificação destes pacientes, é essencial verificar a história do paciente, realizar exame físicos direcionados e avaliar por meio de exames de imagem não invasivos, quando apropriado. Pacientes com DAP assintomática nos membros inferiores frequentemente são acometidos por doença aterosclerótica em coronárias ou doença cerebrovascular e podem merecer terapia antitrombótica para essas indicações^84^ (Quadro 7).

**Quadro 7:** Recomendação no uso de rotina da terapia antitrombótica.

**Recomendações da SBACV:**	
17 Terapia antitrombótica de rotina (antiplaquetário ou anticoagulante) para pacientes com DAP assintomática isolada de membros inferiores assintomáticos não é recomendada.	1A

#### No paciente DAPMI sintomático estável

DAP dos membros inferiores é frequentemente considerada como apenas uma manifestação da aterosclerose sistêmica. Assim, a eficácia dos antitrombóticos na DAPMI é avaliada de acordo com os resultados MACE e MALE, com o benefício do uso de antitrombóticos conferir proteção vascular global; mas deve-se sempre ponderar sobre o risco de sangramento maior e/ou fatal. Pacientes com CI, que nos últimos 6 meses não foram submetidos à revascularização arterial periférica endovascular ou cirúrgica (recente) e sem sintomas agudos de dor em repouso ou perda tecidual, são considerados portadores de DAPMI estável. Embora a terapia antiplaquetária única tenha sido a base da terapia antitrombótica para pacientes com DAPMI sintomática^85,86^ , estudos randomizados recentes, que testaram anticoagulantes de ação direta (DOAC) de baixa dosagem associado com AAS, fornecem dados para novas e importantes evidências para esses pacientes^87,88^ . O benefício do tratamento antiplaquetário vitalício em pacientes com DAP para prevenir DAC ou lesões cerebrovasculares parece convincente^85,86,89^ . Uma metanálise com agentes antiplaquetários, realizada antes de 1997^90^ , incluiu 135.000 pacientes com doença cerebrovascular, doença coronariana ou DAPMI tratados com agentes antiplaquetários, e 77.000 pacientes controle. O grupo de terapia antiplaquetária apresentou redução de 22% nos MACEs, com uso de 75 a 150 mg de aspirina por dia com a mesma eficácia do que doses mais altas, mas com menor risco de sangramento. Outra metanálise^85^ estudou o benefício específico da aspirina em 16 estudos de prevenção secundária com 17.000 pacientes, confirmando o benefício dos agentes antiplaquetários, com uma redução de 18,2% nos MACEs em homens e mulheres. O grupo Critical Leg Isquemia Prevention Study comparou o benefício de 100 mg de aspirina por dia em 185 pacientes com sintomas de DAPMI e ITB < 0,85 ou IHB < 0,6 com placebo, relatando uma redução de 64% no risco de eventos vasculares em comparação com uma redução de 24% no grupo placebo^89,91^ . Historicamente, a ticlopidina foi investigada em vários estudos em doentes com DAP e demonstrou reduzir o risco de IAM, AVC e morte por razões cardiovasculares^91^ . No entanto, seus benefícios são limitados por efeitos colaterais gastrointestinais frequentes, além de neutropenia e trombocitopenia. O clopidogrel, outro derivado tienopiridínico, substituiu a ticlopidina em estudos, comparado com AAS em *A randomised, blinded, trial of clopidogrel versus aspirin in patients at risk of ischaemic events*, demonstrou benefício geral no subgrupo DAPMI, comprovando sua eficácia na redução das taxas de IAM, AVC e mortalidade cardiovascular, com redução do risco relativo de 24%^85,86^ . Nas diretrizes atuais da Sociedade Europeia de Cardiologia, o clopidogrel foi recomendado ao invés da aspirina em pacientes com DAPMI^5^ . A combinação de AAS e clopidogrel em doentes de alto risco, com múltiplos fatores e manifestações aterotrombóticas (incluindo DAPMI), resultou em risco acrescido de hemorragia e nenhum benefício^92^ . O tratamento combinado, em geral, não apresenta reduções de risco estatisticamente significativas em termos de IAM, AVC ou morte cardiovascular, assim, o tratamento combinado não pode ser recomendado para todos os pacientes com DAP. No entanto, dados do estudo PEGASUS-TIMI^93,94^ provam que, em pacientes com DAP com infarto do miocárdio prévio, um subgrupo selecionado de alto risco isquêmico, a terapia antiplaquetária dupla (TAD) com aspirina e ticagrelor de baixa dose (60 mg duas vezes ao dia) está associada à redução de MACE e MALE, com índice de sangramentos aceitáveis. Dessa maneira, pode-se considerar seu uso em pacientes com DAP com infarto do miocárdio prévio até 3 anos após o evento cardíaco; porém, ainda faltam evidências para a indicação de tratamento prolongado com essa associação^5^ (Quadro 8).

**Quadro 8:** Recomendação no uso da antiagregação plaquetária para os pacientes com DAP.

**Recomendações da SBACV:**	
18 Inibidores da agregação plaquetária para a prevenção secundária de eventos cardiovasculares são recomendados em pacientes com DAP sintomática.	1A
19 O clopidogrel pode ser preferível à aspirina.	2B

O papel dos anticoagulantes orais diretos é atualmente objeto de intensa investigação. O estudo *Cardiovascular Outcomes for People Using Anticoagulation Strategies* (COMPASS), um estudo randomizado multicêntrico de 7.470 indivíduos com DAPMI estável constatou que rivaroxabana em dose baixa (um inibidor oral do fator Xa) em combinação com aspirina ocasiona a redução de MACE e MALE em comparação com a aspirina isolada^88^ . Aqueles pacientes designados para uso de rivaroxabana (2,5 mg duas vezes ao dia) associado ao AAS (100 mg uma vez ao dia), tiveram sobrevida total e desfecho cardiovascular 24% melhor (RR 0,76; IC de 95%, 0,66-0,86), porém com mais eventos hemorrágicos graves do que aqueles designados apenas para aspirina (RR 1,70; IC de 95%, 1,40-2,05). A rivaroxabana (5 mg duas vezes ao dia) isoladamente não resultou em melhores resultados cardiovasculares do que a aspirina isolada, e resultou em mais eventos hemorrágicos graves. Claramente, é necessária uma decisão que pondere os riscos de evento CV *versus* sangramento. O benefício líquido foi de 22% de redução geral do risco da população coronariopata e com DAPMI estável^95^ . A análise adicional do subgrupo de DAPMI (DAPMI estável, DAC com DAPMI assintomática e pacientes com estenose carotídea estável) na população do estudo^88,96^ revelou redução significativa adicional de 46% no MALE e taxa de amputação do membro acometido (RR 0,54; IC de 95% 0,35-0,82) para AAS 100 mg/dia combinado com rivaroxabana 2,5 mg duas vezes ao dia, em comparação com AAS 100 mg/dia e placebo. O benefício líquido foi de 28% de redução do risco para o subgrupo DAPMI do COMPASS em comparação com redução de 24% do MACE no subgrupo CAPRIE^85^ . Portanto, para os pacientes onde há preocupação maior do risco isquêmico, como infarto do miocárdio, AVC, isquemia aguda de membro ou amputação maior, pode-se optar por rivaroxabana 2,5 mg duas vezes ao dia em combinação com aspirina. Nos pacientes em que a preocupação é maior com a prevenção de sangramento e minimização da quantidade de comprimidos, pode-se optar pela terapia antiplaquetária única.

Importante também lembrar que o uso de rivaroxabana 2,5 mg duas vezes ao dia em combinação com aspirina deve ser evitado em pacientes com forte influência nas interações medicamentosas com citocromo P450 (CY3A4, CYP2J2) ou ainda com interações da glicoproteína-p nos quadros de insuficiência hepática, diátese hemorrágica ou coagulopatia, além daqueles pacientes com AVC recente (< 1 mês), qualquer AVC hemorrágico prévio e com taxa de filtração glomerular estimada < 15 mL/min^77,84^ (Quadro 9).

**Quadro 9:** Recomendação no uso dos anticoagulantes orais para os pacientes com DAP.

**Recomendações da SBACV:**	
20 Pode-se recomendar o tratamento com rivaroxabana 2,5 mg duas vezes ao dia em combinação com aspirina (80-100 mg ao dia) para o tratamento de pacientes com DAP sintomática dos membros inferiores que apresentam alto risco de eventos isquêmicos e baixo risco de sangramento.	2A
21 O uso adicional de anticoagulantes em dose plena com terapia antiplaquetária, com o objetivo de diminuir eventos MACE e MALE em pacientes com DAP estável em membros inferiores, não é recomendado.	1A
22 A terapia antiplaquetária dupla (TAD), aspirina e clopidogrel ou aspirina e ticagrelor, pode ser considerada para pacientes com DAPMI sintomática com alto risco de eventos vasculares, com baixo risco de sangramento e na contraindicação da rivaroxabana.	2B

Comentário Recomendação 20:

Essa recomendação valoriza muito um único estudo randomizado, controlado, bem desenhado, em oposição a vários estudos menores de baixa qualidade. No entanto, apesar de ser um estudo com uma metodologia robusta e randomizado, a inclusão do desfecho específico de isquemia aguda de membros em cirurgia vascular periférica foi avaliada em um braço pequeno de indivíduos (389 *versus* 516) para eventos secundários. Dessa forma, apesar de parecer promissor a utilização de rivaroxabana em dose reduzida para prevenção de isquemia aguda de membros, acreditamos ser prudente aguardar mais pesquisas que repliquem esses resultados.

#### No uso pós-operatório

##### Antiadesivo plaquetário simples ou em combinação

Para os pacientes com DAPMI que necessitam de revascularização, muitas técnicas endovasculares e cirúrgicas abertas estão disponíveis, incluindo tromboendarterectomia, trombectomia, angioplastia da bifurcação femoral, profundaplastia e procedimentos de ponte utilizando enxertos venosos e sintéticos. Na presença de distúrbios multiníveis, procedimentos híbridos complexos podem ser empregados, envolvendo uma combinação de dilatação por balão e, se necessário, implante de stent para lesões proximais e pontes simultâneas para lesões distais^97^ . As taxas de permeabilidade de todos os procedimentos com pontes nos membros inferiores requerem tratamento adjuvante de inibição plaquetária, independentemente da técnica utilizada^98^ . Cerca de 1/3 dos enxertos venosos dos membros inferiores desenvolvem problemas na própria veia e/ou lesões na região da anastomose, ameaçando sua patência, e observa-se maior risco quando utilizados condutos com menor calibre, veias não safenas, e quando a anastomose é infrapoplítea^99,100^ . As oclusões precoces após pontes venosas e sintéticas são causadas principalmente por problemas técnicos associados a distúrbios do fluxo sanguíneo. Oclusões de médio prazo e tardias podem ser causadas por hiperplasia neointimal no próprio enxerto ou anastomose ou ainda pela progressão da arteriosclerose no leito vascular nativo^101^ . A alta trombogenicidade da superfície interna dos condutos sintéticos faz a principal diferença na ocorrência das oclusões trombóticas, enquanto os venosos são revestidos com endotélio, sendo menos trombogênicos, e as derivações sintéticas raramente mostram camadas de endotélio completamente desenvolvidas^98^ .

O tratamento antiplaquetário é recomendado para melhorar as taxas de perviedade após cirurgia com pontes infrageniculares, enquanto é mais eficaz em enxertos sintéticos do que em condutos autólogos^97,102-104^ . Uma metanálise, abrangendo onze estudos clínicos randomizados realizados antes de 1990, mostrou que a administração de agentes antiplaquetários reduz significativamente o risco de oclusão de pontes, em 32%^105^ . Corrobora o mesmo resultado outra metanálise de cinco estudos, que explorou o AAS (sozinho ou em combinação com outros medicamentos antiplaquetários) *versus* placebo em pacientes com pontes infrainguinais. As oclusões de pontes foram observadas em 28,4% dos 423 indivíduos que receberam tratamento antiplaquetário, e em 36,6% dos 393 indivíduos que receberam placebo, com risco relativo na redução de oclusão do enxerto infra inguinal (RR: 0,78) nos pacientes usando AAS^106^ . Em pacientes que já recebem tratamento antiplaquetário duplo, os riscos intra e perioperatório de trombose arterial devem ser balanceados com os riscos individuais de sangramento. A experiência clínica demonstrou que o clopidogrel deve ser descontinuado idealmente em 8 a 10 dias antes da cirurgia vascular devido ao risco aumentado de sangramento, e que o tratamento com AAS deve ser continuado. No entanto, os riscos cardiovasculares, por exemplo implantes recentes de stents coronários farmacológicos, devem ser considerados^107^ . O estudo *Bypass Surgery for Peripheral arterial disease* (CASPAR)^104^ randomizou 851 indivíduos para receber somente AAS (75 a 100 mg) ou clopidogrel combinado (75 mg) ao AAS, e não observou diferença significativa nas taxas de perviedade. Uma análise de subgrupo, no entanto, encontrou benefício significativo do tratamento combinado na ponte utilizando conduto sintético sobre o uso de veia, podendo assim ser considerada a inibição plaquetária dupla com AAS e clopidogrel no pós-operatório de ponte utilizando prótese infragenicular. Dois importantes estudos em revascularização aberta incluem o Dutch BOA^108^ e o estudo VOYAGER PAD^109^ . No estudo Dutch BOA, o tratamento usando monoterapia com antagonista de vitamina K (AVK) (INR= 3,0-4,5), em comparação com monoterapia usando aspirina, não diminuiu significativamente a oclusão do enxerto ou MACE e aumentou significativamente a taxa de sangramento maior (RR, 1,96; IC de 95%, 1,42-2,71). A análise de subgrupo mostrou, no entanto, permeabilidade melhorada com AVK em pontes de conduto venoso (RR, 0,69; IC de 95%, 0,54-0,88) e com ácido acetilsalicílico em enxertos sintéticos (RR, 1,26; IC de 95%, 1,03-1,55). O estudo *VOYAGER-PAD* avaliou a segurança e a eficácia do TAD (rivaroxabana 2,5 mg/2vezes/dia + aspirina) *versus* monoterapia com AAS, iniciado 10 dias após a revascularização, em 6.564 pacientes submetidos a cirurgia ou revascularização endovascular dos membros inferiores. Durante acompanhamento médio de 28 meses, com taxa de eficácia primária, isquemia aguda do membro, amputação maior, infarto do miocárdio (IM), AVC isquêmico ou morte cardiovascular foram significativamente reduzidas usando-se terapia antiagregante dupla (TAD) *versus* aspirina (15,5%, vs. 17,8%, p = 0,009). Em relação à segurança, sangramento maior TIMI ocorreu em 2,65% no grupo TAD e em 1,87% no grupo aspirina (p = 0,07). Aproximadamente 50% dos pacientes também receberam clopidogrel em ambos braços do estudo, por no máximo 6 meses, principalmente após a terapia endovascular. O benefício do efeito do TAD foi independente do clopidogrel, mas o risco de sangramento maior (critérios ISTH) foi maior quando clopidogrel foi administrado por mais de 1 mês, e aumentou **c**om o tempo^110^ .

##### Uso de heparina

A heparina não fracionada é tradicionalmente administrada por via intravenosa antes do clampeamento arterial, na tentativa de prevenir a trombose causada por estase nos segmentos vasculares proximal e distal bem como no segmento anastomótico. Nenhum estudo randomizado abordou essa indicação, que provavelmente perdeu sua legitimidade devido à experiência de longo prazo. Recomendações gerais para monitoramento intraoperatório de anticoagulação, por exemplo, com base no tempo de coagulação ativada, não são possíveis devido aos poucos dados disponíveis^108^ .

##### Anticoagulante via oral – AVK

O estudo randomizado com 2.690 participantes, submetidos a pontes venosas ou sintéticas, comparando o uso de AVK com INR3 a 4,5 ou ASA 80 mg/d^109^ , não demonstrou benefício nos dois grupos quanto ao uso antitrombótico e quanto à perviedade da ponte femoro-poplíteo *versus* femoro femoral (RR 0,95; IC de 95% 0,82-1,11). No entanto, uma análise post-hoc mostrou um risco de oclusão significativamente menor entre pacientes com ponte venoso utilizando AVK, enquanto os riscos de oclusões dos substitutos sintético, utilizando AAS, foram significativamente menores. Esses resultados são frequentemente usados como argumento a favor do AVK após ponte venoso. Pacientes em tratamento com AVK apresentaram episódios hemorrágicos de larga escala significativamente mais frequentes em comparação com pacientes em tratamento com AAS (RR 1,96; IC de 95% 1,42-2,71). Mesmo na presença de valores adequados de INR, os participantes do estudo com idade superior a 72 anos, com diabetes e/ou hipertensão arterial, apresentaram riscos aumentados de sangramento. De acordo com os critérios de evidência, as análises post-hoc não fornecem validade suficiente para facilitar recomendações gerais, além da faixa alvo utilizada no estudo não ser a utilizada de usualmente, mas somente para situações com alto risco de tromboembolismo, dificultando a tradução desses resultados para o cenário clínico. Os AVKs não melhoraram a perviedade do enxerto protético, mas são ligeiramente benéficos em condutos venosos^111^ . O tratamento combinado com AAS e AVK é considerado uma opção principalmente para os pacientes com dificuldade de acesso ao uso de DOAC, e deve ser monitorado com frequência, devido ao maior risco de sangramento (Quadro 10).

**Quadro 10:** Recomendação no uso de TAS ou combinada após procedimento de revascularização dos MMII.

**Recomendações da SBACV:**	
23 Recomenda-se TAS de longo prazo em pacientes após revascularização.	1C
24 Recomenda-se TAS de longo prazo após cirurgia com ponte infrainguinal.	1A
25 Antagonistas da vitamina K podem ser considerados após ponte infrainguinal utilizando veia autóloga.	2B
26 TAD com aspirina e clopidogrel pode ser considerada em pontes abaixo do joelho com enxerto protético.	2B

##### No uso pós-procedimentos endovasculares

A inflamação vascular local após angioplastia com balão ou implantação de stent é a principal responsável por processos de reestenose de médio e longo prazo, enquanto a^112^ trombose precoce e a oclusão geralmente se desenvolvem como resultado de dissecção ou ativação plaquetária arterial local. As taxas de reestenose ou reoclusões dependem não só da morfologia vascular e do tipo de técnica endovascular ou stent implantado escolhido, mas também da região vascular. Por exemplo, implantes de stent nas regiões femoro-poplíteas estão associados a risco de reestenose acentuadamente maior do que nos vasos ilíacos, possivelmente causado por níveis mais elevados de inflamação local nas artérias musculares do leito vascular femoral, em oposição a reações inflamatórias menos graves nas artérias elásticas do leito vascular pélvico^113^ . A inflamação crônica das paredes vasculares também pode afetar a reestenose e o risco de oclusão após intervenções bem-sucedidas, como ocorre de maneira mais frequente nos pacientes com insuficiência renal crônica avançada e nos diabéticos devido aos distúrbios metabólicos^114^ . A ativação plaquetária é elevada em pacientes com DAP, indicando uma tendência trombótica intra-arterial aumentada^115^ , tornando o uso de terapia antiplaquetária recomendado para todos os pacientes com DAP sintomática, independentemente de intervenções profiláticas secundárias, a fim de reduzir a morbidade e mortalidade cardiovascular e cerebrovascular associadas.

Recomendações baseadas em evidências sobre os tratamentos antitrombóticos mais benéficos para escolha, dose e duração da terapia com drogas antitrombóticas em relação aos procedimentos endovasculares não são claras. Da mesma forma, a questão de saber se uma dose de ataque de clopidogrel (300 mg *versus* 600 mg) deve ser administrada antes de intervenções periféricas planejadas com implantes de stents monoterapia não é clara, pois nenhum estudo abordou esse tema. Uma metanálise da Cochrane, incluindo 3.529 pacientes, avaliou drogas antitrombóticas para prevenção de reestenose ou reoclusão^116^ , onde nenhuma redução foi encontrada com aspirina mais dipiridamol versus aspirina mais placebo (OR 0,69; 95% e IC de 95%, 0,44-1,10), sendo a TAD frequentemente usada, geralmente entre 1 e 3 meses, após procedimentos endovasculares com grande variabilidade quanto à sua duração^117^ . Quando necessária a colocação de stent nas artérias infrapoplíteas, a duração do uso de TAD costuma ser de longo tempo, mas sem evidências disponíveis. A duração do TAD baseia-se principalmente na extrapolação do uso de stent no território coronariano, o que pode não ser adequado devido a provável maior reatividade plaquetária residual em resposta ao difosfato de adenosina e ácido araquidônico, encontrados em pacientes com DAP dos MMII^118^ . É possível, portanto, que pacientes submetidos à angioplastia periférica dos MMII tenham uma resposta mais fraca à aspirina e ao clopidogrel em comparação a pacientes com intervenção coronária percutânea. No estudo *Management of Peripheral Arterial Interventions with Mono or Dual Antiplatelet Therapy* (MIRROR), TAD usando AAS e clopidogrel foi comparado com AAS isolada em 80 pacientes após revascularização endovascular de membros inferiores, onde a TAD melhorou as taxas de revascularização da lesão alvo em 6 meses (5% *versus* 8%), mas não em 1 ano^119^ . Uma análise retrospectiva de 693 pacientes submetidos à revascularização endovascular mostraram que TAD >6 meses foi um preditor independente de risco reduzido para MACE (RR 0,61; IC de 95% 0,40-0,93) e MALE (RR 0,55; IC de 95% 0,38-0,77), sem aumento significativo de sangramento^120^ . Estudos randomizados controlados investigaram até que ponto os AVKs constituiriam uma alternativa ao tratamento antiagregante após angioplastia periférica fêmoro-poplítea e distais. Um total de 438 pacientes foram randomizados, e todos os grupos tratados com AVK apresentaram taxas mais baixas de permeabilidade arterial, com taxas de sangramento significativamente mais altas (RR 1,79; IC de 95% 1,3-4,6)^98,118,121^ . As drogas antiplaquetárias devem, portanto, ser de primeira linha nos tratamentos intervencionistas, desde que não haja outras indicações imperativas para AVK, principalmente decorrentes de comorbidades cardiológicas, como a FA. A duração do tratamento duplo e a questão de saber se as doses de ataque de clopidogrel são eficazes ou necessárias na periferia permanecem obscuras.

Estudos que abordam o tratamento com cilostazol em pacientes com CI e doença fêmoro-poplítea após intervenções endovasculares também são pequenos, embora prospectivos. Em uma análise populacional retrospectiva em grande escala nos EUA^122^ , verificando dados com mais de 23.000 indivíduos com DAPMI, investigando o cilostazol após revascularizações cirúrgicas endovasculares e abertas, o AAS foi administrado a 20.335 pacientes como profilaxia secundária, e 1.999 receberam cilostazol adicional, sendo encontradas taxas significativamente reduzidas de reestenose e amputação maior no grupo que recebeu cilostazol antes e/ou após o tratamento. Outros estudos sobre cilostazol adicional após intervenções fêmoro-poplíteas com e sem stents foram na mesma direção. Cinco séries de casos retrospectivos e dois pequenos estudos prospectivos mostraram uma redução na taxa de reestenose após o tratamento com cilostazol^123^ . Na condição de não haver contraindicações e na presença de tolerabilidade adequada, o tratamento combinado com AAS e cilostazol pode ser considerado para melhorar a permeabilidade e reduzir as taxas de amputação após o tratamento endovascular infrainguinal. Uma metanálise com 3.136 pacientes com tempo médio de acompanhamento de 2 anos em todos os estudos revelou que o tratamento com cilostazol favoreceu a sobrevida livre de amputação (RR 0,79; IC de 95%, 0,69-0,91), taxa de salvamento de membros (RR, 0,42; IC de 95%, 0,27-0,66), diminuição da necessidade de nova revascularização (RR 0,44; IC de 95%, 0,37-0,52) e diminuição da reestenose (RR, 0,68; IC de 95%, 0,61-0,76). O tratamento com cilostazol também aumentou a patência da revascularização da lesão alvo (RR, 1,35; IC de 95%, 1,21-1,53), sem diferença na mortalidade por todas as causas. A cicatrização efetiva de feridas foi considerada uma medida de resultado inconsistente em pacientes recebendo terapia com cilostazol^124^ (Quadro 11).

**Quadro 11:** Recomendação no uso de terapia antitrombótica após angioplastia dos MMII.

**Recomendações da SBACV:**	
27 Recomenda-se o uso de TAD por pelo menos 1 mês após angioplastia com balão farmacológico ou pelo menos 3 meses após implante de stent farmacológico ou implante de stent recoberto.	1C
28 O uso de TAD com aspirina e clopidogrel por pelo menos 1 mês deve ser considerado após implante de stent infrainguinal.	2C
29 Medicamentos antivitamina K (AVK) não são recomendados após angioplastia do território fêmoro-poplíteo ou distais com único objetivo de prevenir reestenose ou reoclusões.	2A
30 Em pacientes que necessitam do uso de AVK contínua por outra razão (por exemplo, para FA) após a revascularização endovascular, deve-se considerar o uso de aspirina ou clopidogrel, além de AVK por pelo menos 1 mês se o risco de sangramento for baixo em comparação com o risco de oclusão do stent/enxerto.	2B
31 O tratamento combinado com AAS e cilostazol pode ser considerado para melhorar a permeabilidade e reduzir as taxas de amputação após o tratamento endovascular infrainguinal.	2A

O estudo VOYAGER PAD randomizou pacientes após revascularização endovascular ou aberta para terapia combinada de rivaroxabana 2,5 mg duas vezes ao dia com aspirina, ou apenas aspirina com a opção de usar clopidogrel adicionalmente até um máximo de 6 meses, a critério do médico assistente^110^ . A combinação de rivaroxabana e aspirina reduziu eventos MACE e MALE compostos (RR, 0,85; IC de 95%, 0,76-0,96), impulsionado principalmente por redução significativa na isquemia aguda do membro (RR, 0,67; IC de 95%, 0,55-0,82), onde a maioria dos procedimentos de revascularização foram realizados para a indicação de piora da claudicação (76,6%), e até 1/3 dos pacientes apresentavam isquemia crítica do membro. Não houve diferença significativa no desfecho de segurança primário de sangramento maior (TIMI), mas no desfecho de segurança secundário da ISTH o sangramento maior foi aumentado (RR, 1,42; IC de 95% CI, 1,10-1,84), embora sem aumentos significativos no sangramento intracraniano ou fatal. Aproximadamente 50% dos participantes do estudo receberam clopidogrel, em média por 30 dias, sem alterar a eficácia de rivaroxabana. No entanto, naqueles com cursos mais longos de clopidogrel (superior há 30 dias) houve uma tendência de aumento de sangramento maior, com risco absoluto de 2,71% (RR, 3,20; IC de 95% CI, 1,44-7,13), enquanto < 30 dias de clopidogrel conferiu menor risco absoluto de sangramento maior, de 0,46%^111^ . Dois terços da população de pacientes foram submetidos à intervenção endovascular, tornando esse o maior estudo conduzido até o momento para avaliação dessa terapêutica (Quadro 12).

**Quadro 12:** Recomendação no uso dos anticoagulantes orais após revascularização dos MMII.

**Recomendações da SBACV:**	
32 Pode-se recomendar o tratamento com rivaroxabana 2,5 mg duas vezes ao dia em combinação com aspirina (80-100 mg ao dia) para pacientes com DAPMI após revascularização aberta eletiva, para diminuição de desfechos compostos em pacientes com baixo risco de sangramento. Não se recomenda o uso para aumento de perviedade.	2A
33 Pode-se recomendar rivaroxabana 2,5 mg duas vezes ao dia em combinação com aspirina (80-100 mg ao dia), e com clopidogrel até 30 dias. Sem associação do clopidogrel, pode-se usar por mais tempo em pacientes com DAPMI após revascularização aberta ou endovascular eletiva.	2A

Comentário Recomendações 32 e 33:

Essa recomendação valoriza muito um único estudo randomizado, controlado, bem desenhado, com muitos pacientes, em oposição a vários estudos menores de baixa qualidade. Essa recomendação também valoriza pacientes com risco isquêmico no contexto de aumentos aceitáveis do risco geral de sangramento. Portanto, o uso adicional de clopidogrel (75 mg ao dia) com rivaroxabana 2,5 mg duas vezes ao dia e aspirina (80-100 mg ao dia) pode ser considerado em pacientes submetidos a stent endovascular complexo, por um período de no máximo 30 dias, iniciando após 10 dias da revascularização. Deve-se preferencialmente continuar a terapia (rivaroxabana + AAS) por longo prazo na ausência de sangramento ou manifestações isquêmicas, uma vez que a revascularização prévia representa alto risco para recorrência. Dessa forma, apesar de parecer promissora a utilização de rivaroxabana em dose reduzida para prevenção eventos isquêmicos dos membros, após a revascularização, acreditamos também ser prudente aguardar mais estudos que repliquem esses resultados.

### Atividade fisica para DAP

Evidências de alta qualidade mostram que programas de exercícios forneceram benefícios importantes na melhora da distância máxima sem dor em pessoas com CI. O exercício não melhorou o ITB e não há evidências de efeito na taxa de amputação ou mortalidade. O exercício pode melhorar a qualidade de vida quando comparado com placebo ou cuidados habituais^125^ . Modos alternativos de exercício podem ser úteis quando o exercício de caminhada supervisionada não é uma opção^126^ . Pacientes submetidos a exercícios domiciliares não supervisionados, porém adicionados de uma intervenção comportamental cognitiva em grupo, apresentaram melhora no desempenho de caminhada e de atividade física, sendo uma alternativa para pacientes sem oportunidade de receber a terapia de exercício supervisionado^127^ . Treinamento de caminhada estruturada e supervisionada demonstrou ser superior à caminhada não supervisionada, programas de treinamento de caminhada realizados por pelo menos 3 meses sob supervisão mostraram aumento na capacidade de caminhar e uma diminuição na gravidade da claudicação^128,129^ , sendo eficaz quando realizado pelo menos três vezes por semana, durante 30 a 60 minutos^130,131^ . Estudos controlados em pacientes com claudicação demonstraram aumentos de até 200% nas distâncias de caminhada após 12 semanas de treinamento^132,133^ . Em outros estudos, os resultados funcionais a longo prazo do treinamento de caminhada também se mostraram equivalentes às intervenções vasculares isoladas^134,135^ . No entanto, os efeitos da revascularização endovascular e do treinamento de caminhada são aditivos^136^_,_ sendo que em ambas as modalidades de tratamento pode-se fazer parte de uma abordagem integrativa.

Também foi demonstrado que o treinamento de membros superiores pode beneficiar a função endotelial em pacientes com DAPMI, devendo ser tentado se o treinamento de caminhada não for possível. Os fatores prognósticos favoráveis no treinamento de caminhada incluem os seguintes critérios: menos de 1 ano após o diagnóstico de DAP, oclusão da artéria femoral e boa condição cardiopulmonar. Um estudo^137^ demonstrou que o treinamento de resistência com ergômetro de braço pode produzir melhorias nas distâncias de caminhada sem dor, são comparáveis a consumo calórico semelhante ao treinamento em esteira, podendo ser uma alternativa complementar ao treino de caminhada. O treinamento regular de caminhada também mostra modificações benéficas adicionais do metabolismo da glicose e lipídios^138,139^ .

Enquanto as lesões vasculares não forem recanalizadas com sucesso por intervenções endovasculares ou cirurgia convencional, o treinamento de caminhada não é eficaz em estenoses da artéria femoral profunda e oclusão da AFS ipsilateral. Assim, a recanalização vascular deve ser realizada em lesões vasculares pélvicas, lesões da bifurcação femoral e estenoses ou oclusões da artéria femoral profunda antes que o tratamento básico com treinamento de caminhada seja introduzido^133,140^ . Em pacientes com estenose de alto grau ou oclusão da artéria poplítea, o exercício pode ter efeito limitado na claudicação devido às opções reduzidas de colateralização. Portanto, a revascularização pode ser recomendada nesse grupo antes do treinamento físico, porém, o resultado cirúrgico pode estar associado a menores índices de patência a longo prazo. É preciso ressaltar que aproximadamente 50% dos pacientes com DAPMI apresentam distúrbios ortopédicos e/ou neurológicos concomitantes e/ou déficits funcionais cardiopulmonares, que podem impedir o treinamento de caminhada ou impossibilitar a participação em grupos de esportes vasculares estruturados. Tais comorbidades devem ser registradas antes do treinamento de caminhada, e modificações devem ser introduzidas para facilitar a participação no treinamento de caminhada para o maior número possível de pacientes (Quadro 13).

**Quadro 13:** Recomendação na atividade física para os pacientes com DAPMI.

**Recomendações da SBACV:**	
34 Treinamento com exercício supervisionado é recomendado para os pacientes com DAPMI.	1A
35 Recomenda-se treino supervisionado em pacientes com claudicação intermitente de no mínimo 3 vezes por semana, no mínimo de 30 minutos, durante no mínimo 3 meses.	1B
36 O treinamento físico não supervisionado é recomendado quando o treinamento físico supervisionado não é viável ou disponível.	1C

## TRATAMENTO DA DAPMI

### Indicação para revascularização

Os tratamentos cirúrgicos estão associados a uma maior morbidade e mortalidade bem como a custos mais elevados, e particularmente na presença de comorbidades há um aumento dos riscos perioperatório para os doentes. Os tratamentos endovasculares são caracterizados por níveis mais baixos de invasibilidade e menores taxas de complicações, mas requerem frequentemente tratamentos adicionais, principalmente reintervenções devido à reestenose ou reoclusão. Com base nessas vantagens e desvantagens, riscos e benefícios individuais devem ser avaliados para a indicação do melhor tratamento. O principal objetivo a longo prazo do tratamento em pacientes com isquemia crônica que ameaça o membro é a sobrevida livre de amputação, além da melhora na cicatrização das úlceras, melhora na dor isquêmica e diminuição das taxas de mortalidade nestes pacientes com alto risco cardiovascular. Nos pacientes claudicantes, o objetivo do tratamento é um pouco diferente, pois o paciente busca uma melhora na qualidade de vida devido ao incômodo da dor ao caminhar.

#### Tratamento da claudicação

A história natural, após o primeiro ano de diagnóstico da claudicação intermitente, geralmente inclui risco anual de 2 a 3% de progressão para isquemia crônica, com possibilidade de perda do membro^141,142^ , sendo o risco anual de amputação de 1% nesses pacientes^142,143^ . O manejo da claudicação intermitente é realizado predominantemente pela modificação dos fatores de risco para aterosclerose associado a exercícios regulares e supervisionados^144,145^ , o que resulta em aumento das distâncias de caminhada e pode ser igualmente eficiente para revascularizações por via endovascular ou cirúrgica^136^ .

O cilostazol, um inibidor da fosfodiesterase 3 é um vasodilatador que inibe a proliferação de células musculares lisas vasculares e previne a agregação plaquetária, usado como tratamento para melhorar os sintomas de caminhada em pacientes com claudicação intermitente com DAP, sendo indicado quando os sintomas persistem e afetam a qualidade de vida. Seu mecanismo de melhora dos sintomas de claudicação não está claro e é provavelmente multifatorial, sendo a dose recomendada de 100 mg duas vezes ao dia e deve ser tomada pelo menos 30 minutos antes ou 2 horas depois do café da manhã e do jantar^146^ .

Porém há pouca evidência em estudos randomizados, de tal beneficio, na ultima revisão Cochrane^147^ pode-se observar que participantes que tomaram cilostazol durante três a seis meses, conseguiram caminhar uma distancia maior para inicio da dor, do que aqueles que utilizaram placebo, porem não houve evidencia robusta para comprovar a melhora na qualidade de vida destes pacientes, sendo o aumento desta distancia percorrida sem dor, específico para cada paciente (Quadro 14).

**Quadro 14:** Recomendação no uso do cilostazol para os pacientes claudicantes com DAP.

**Recomendações da SBACV:**	
37 Recomenda-se que seja considerado o cilostazol, nos pacientes claudicantes com DAP, apenas se a qualidade de vida destes pacientes for substancialmente limitada e o treino de caminhada for restrito, inviável ou ineficaz.	1B
38 Recomenda-se que o tratamento com estes agentes seja interrompido se os sintomas não melhorarem após 3 meses.	1C

No entanto, a revascularização como tratamento da claudicação pode ser considerada em pacientes que continuam a apresentar sintomas limitantes no estilo de vida ou profissão apesar do tratamento clínico otimizado, ocorrendo com muita frequência nos quadros de oclusões aorto-ilíacas e da artéria poplítea^134,148^ . A sintomatologia da claudicação tende a causar maior incapacidade em topografias mais proximais, quando comparadas às oclusões mais distais^149^ . Diversos estudos mostram uma taxa de perviedade cumulativa similar no médio prazo em revascularizações cirúrgicas e endovasculares, nos segmentos aorto-ilíacos e fêmoro-poplíteos. A intervenção endovascular apresenta menores complicações perioperatórias, porém uma taxa maior de reestenose, que geralmente é manejada com reintervenção também por via endovascular^150-152^ . O mesmo benefício não é observado no território infrapoplíteo, demandando uma avaliação individualizada mais criteriosa^153^ . No insucesso do tratamento conservador, o tratamento intervencionista pode produzir melhorias na qualidade de vida a curto e médio prazo, associado à melhora na capacidade de caminhar sem dor^154^ . Porém, no paciente claudicante, os critérios para tratamento cirúrgico de revascularização e/ou angioplastia devem ser mais rigorosos, pois a longo prazo as taxas de mortalidade e preservação do membro, quando comparadas ao tratamento conservador, não são melhores^155^ . Portanto, deve-se informar ao paciente que a falha na intervenção pode acarretar consequências graves para os membros (Quadro 15).

**Quadro 15:** Recomendação na indicação do tratamento cirúrgico para os pacientes claudicantes com DAP.

**Recomendações da SBACV:**	
39 Recomenda-se que o tratamento inicial do paciente claudicante seja realizado através de medidas clínicas e não cirúrgicas.	1A
40 Recomenda-se a revascularização do paciente claudicante quando ocorrer falha do tratamento clínico, com manutenção dos sintomas severos e impacto significativo na qualidade de vida, relacionados principalmente a lesões oclusivas em território aorto-ilíaco, ilíaco-femoral e fêmoro-poplíteo, incluindo a artéria poplítea proximal.	1B
41 Em pacientes com claudicação de curta distância, em que o treinamento de caminhada se mostrou impossível ou malsucedido, e na presença de lesões arteriais adequadas, o tratamento intervencionista é recomendado para melhora da qualidade de vida.	1C
42 Não se recomenda a realização de pontes fêmoro-tibiais para o tratamento da claudicação intermitente.	1B

Estudos comparativos entre caminhada supervisionada e abordagem endovascular na claudicação recomendam uma abordagem inicial não intervencionista, pela boa resposta do tratamento clínico nos pacientes com claudicação estável. Apesar disso, a associação da intervenção endovascular pode trazer benefício adicional mais precoce na distância de caminhada na esteira e na qualidade de vida, quando comparada ao tratamento clínico isolado, às custas de menor benefício a longo prazo^137,156-159^ . A abordagem endovascular pode ser indicada como opção de tratamento nos casos de perviedade assistida, quando ocorrem estenoses significativas, em revascularizações prévias^160^ . Por essa razão, consideramos a intervenção endovascular como uma opção no tratamento destes pacientes (Quadro 16).

**Quadro 16:** Recomendação na abordagem endovascular para os pacientes claudicantes com DAP.

**Recomendações da SBACV:**	
43 A intervenção endovascular em pacientes claudicantes com lesões de repercussão hemodinâmica significativa não é recomendada usualmente como abordagem profilática, mas pode ser considerada como opção de tratamento naqueles pacientes que já foram previamente revascularizados.	2B

#### Isquemia crônica que ameaça o membro

A isquemia crônica que ameaça o membro é a forma mais avançada de DAPMI, na qual os pacientes geralmente apresentam sinais de arteriopatia, como dor isquêmica em repouso, perda de tecido ou gangrena, e, ao contrário da claudicação, tem uma história natural pior, com progressão mais rápida para perda de tecido e do membro^161^ . Apesar dos avanços na terapia farmacológica e na compreensão melhor na redução dos fatores de risco para DAPMI^162^ , os pacientes com ICAM continuam a apresentar alta mortalidade e taxas de amputação maior de 22% em 1 ano quando não submetidos a revascularização^163^ . Nesse perfil de paciente, a revascularização imediata tem maior importância no resultado do tratamento, quando comparada ao paciente com claudicação^164^ (Quadro 17).

**Quadro 17:** Recomendação na avaliação inicial para o paciente com isquemia crônica que ameaça o membro.

**Recomendações da SBACV:**	
44 Recomenda-se que todos os pacientes com isquemia crônica que ameaça o membro sejam encaminhados com urgência a especialistas vasculares, para consideração de revascularização.	1C
45 Recomenda-se que em pacientes com isquemia crônica com ameaça ao membro, a revascularização seja endovascular, aberta ou híbrida. Deve-se considerar o padrão anatômico da doença, grau de isquemia, durabilidade esperada do procedimento, risco perioperatório e expectativa de vida do paciente.	1C

##### Avaliação e planejamento do paciente com ICAM

A avaliação diagnóstica e o estadiamento com métodos de imagem dos pacientes com suspeita de ICAM são parte integrantes do sucesso do tratamento. Atualmente, os avanços tecnológicos de imagem tornaram o diagnóstico do ICAM mais preciso, o que permite uma melhor seleção e planejamento dos pacientes para revascularização. No entanto, o acesso a modalidades diagnósticas sofisticadas de imagens vasculares varia consideravelmente em todo o mundo e também no nosso país, nas diferentes formas de atendimento, privado ou pelo sistema único de saúde. Diferentes condutas são empregadas, muitas vezes sem padronização, devido à limitação de recursos destinados à saúde^165^ . Portanto, esta diretriz tem como objetivo estabelecer princípios e considerações amplas que possam ser usadas para orientar e padronizar a avaliação e o tratamento, de forma mais efetiva possível para os pacientes.

Além da anamnese e do exame físico focados na correção dos fatores de risco e cuidados locais da ferida, existe a tendência de se usar sistemas de classificação de membros e feridas para qualificar a tomada de decisões e, assim, obter uma melhora nos resultados para os pacientes. Um sistema de classificação que vem sendo recomendado e utilizado nas diretrizes para estratificar o resultado do tratamento foi desenvolvido pela SVS, com base em características da ferida (*wound*, W), grau de isquemia (*ischemia*, I),e presença e gravidade da infecção (*foot infection*, fI): a classificação Wound, Ischemia and foot Infection (WIfI), que correlaciona a probabilidade de salvamento do membro e a cicatrização das feridas após a revascularização (Tabela 3), procurando auxiliar na tomada de decisão para revascularizar os pacientes que são candidatos. Essa classificação foi estabelecida a partir de um consenso de especialistas, porém ainda necessita de validação. Outro sistema para auxiliar na parametrização de conduta é o Sistema de Estadiamento Anatômico Global (Global Limb Anatomic Staging System – GLASS) (Tabela 4 e 5)^166^ , usado para somar informações e auxiliar de maneira mais efetiva na escolha da melhor estratégia de revascularização, considerando o risco do paciente, o estadiamento do membro e o padrão anatômico da doença^167,168^ .

**Tabela 3:** Classificação de risco de amputação da Society For Vascular Surgery (SVS), WIfI.

**Componente**	**Score**	**Descrição**		
W	0	Sem úlcera dor isquêmica ao repouso		
Wound/Ferida	1	Úlcera pequena ou superficial na perna distal ou pé sem gangrena		
	2	Úlcera profunda com exposição de osso, articulação ou tendão ± gangrena limitada aos dedos		
	3	Úlcera profunda e extensa, úlcera de calcâneo ± úlcera de calcâneo ± gangrena extensa		
I		ABI (índice Tornozelo-Braço)	Pressão do Tornozelo (mmHg)	Pressão do Hálux TcPO_2_
Isquemia				
	0	≥ 0,80	> 100	≥ 60
	1	0,60-0,79	70-100	40-59
	2	0,40-0,59	50-69	30-39
	3	≤ 0,39	< 50	< 30
FI	0	Sem sintomas / Não infectado		
Foot Infection/Infecção do pé				
	1	Infecção local leve, envolvendo apenas pele e tecido subcutâneo		
	2	Infecção local moderada, envolvendo mais tecidos além da pele ou tecido subcutâneo		
	3	Infecção local grave com os sinais da síndrome da resposta inflamatória sistêmica		

**Tabela 4:** Classificação GLASS, original para doença fêmoro-poplítea.

**Classificação Fêmoro-Poplítea**	
0	Doença leve ou não significativa (< 0%).
1	Doença envolvendo < 1/3 (< 10 cm) da AFS, podendo incluir oclusão total focal única (< 5 cm), mas que não envolva a origem da AFS; artéria poplítea com doença leve ou sem doença significativa.
2	Comprimento total da doença na AFS de 1/3-2/3 (10-20 cm); pode incluir OTC da AFS totalizando < 1/3 (10 cm), mas que não envolva a origem da AFS; estenose focal da artéria poplítea < 2 cm, não envolvendo trifurcação.
3	Doença na AFS com comprimento total de >2/3 de extensão (>20 cm do vaso); pode incluir qualquer oclusão < 20 cm que envolva a origem do vaso ou OTC que não envolva a origem do vaso com 10-20 cm de comprimento; estenose da artéria poplítea curta de 2 a 5 cm, não envolvendo trifurcação.
4	Comprimento total de oclusão da AFS >20 cm; doença poplítea >5 cm ou estendendo-se para trifurcação; qualquer OTC da artéria poplítea.

AFS = Artéria femoral superficial; OTC = oclusão total crônica.

**Tabela 5:** Classificação Glass original para doença infrapoplítea.

**Classificação infrapoplítea**	
0	Doença leve ou não significativa (< 50%).
1	Estenose focal < 3 cm sem incluir o tronco TF.
2	Estenose com comprimento total da doença arterial alvo <1/3; pode incluir oclusão focal única (< 3 cm), não incluindo o tronco TF ou a origem da artéria alvo.
3	Comprimento total da doença arterial alvo com 2/3 da extensão do vaso; OTC† acima de 1/3 da extensão do vaso (pode incluir a origem da artéria alvo, mas não o tronco TF).
4	Estenose arterial difusa sendo > 2/3 do seu comprimento; OTC > 1/3 do seu comprimento (pode incluir a origem da artéria alvo); qualquer OTC do tronco TF se a arterial tibial anterior não for a artéria alvo.

TF = tronco tíbiofibular; OTC = oclusão total crônica.

Para decidir qual o melhor método de revascularização a ser empregado para cada paciente, deve-se avaliar os diversos aspectos, e não somente a anatomia^169^ . A complexidade da estratégia de revascularização necessita considerar aspectos anatômicos da lesão, expectativa de reabilitação pelo paciente, risco operatório, condições locais do serviço e preferências do paciente. Diretrizes recentes têm utilizado a classificação GLASS e WIfI para auxiliar na escolha do método de revascularização mais efetivo^170,171^ , que não deverão ser usadas como guias únicos para orientar a conduta, mas auxiliar a complexa análise que deve ser feita caso a caso. Como nos casos de pacientes com WIfI 1: a princípio, não devem ser revascularizados, uma vez que possuem fluxo suficiente para a cicatrização tecidual. No entanto, há pacientes que, mesmos classificados como de risco muito baixo de amputação ou de revascularização, evoluem sem cicatrização adequada. Alguns biomarcadores têm sido estudados como preditores de perda do membro, em especial em pacientes com WIfI baixo^172,173^ . A avaliação clínica longitudinal, ou seja, o acompanhamento do paciente, é essencial para definir a indicação de revascularização, sendo que pacientes que não estão cicatrizando mesmo com as medidas locais e sistêmicas adequadas devem ser considerados para a revascularização^174^ . A Tabela 6^175^ demonstra que cirurgia de revascularização aberta e a terapia endovascular têm papéis complementares, com notável falta de consenso nas faixas intermediárias de complexidade clínica e anatômica (em verde). Estudos sobre classificações e estadiamento de lesões são necessários, para melhorar a qualidade da evidência de intervenções em cenários clínicos específicos. Pacientes sem conduto autólogo adequado devem ser considerados separadamente, uma vez que esse é um fator crítico na determinação do provável sucesso e durabilidade da cirurgia de revascularização com ponte. Mesmo na ausência de veia safena adequada, as derivações com veias de braço e veias emendadas têm melhor desempenho do que os enxertos não autólogos para tratamentos distais, necessitando vigilância mais frequente e mais reintervenções para manter a perviedade assistida primária^176^ . Em pacientes com isquemia crônica que ameaça o membro e bom risco cirúrgico, deve-se avaliar a gravidade da ameaça ao membro (classificação WIfI), o padrão anatômico das lesões vasculares (classificação GLASS) e a disponibilidade de veia autóloga para confecção da ponte, para decidir entre revascularizarão por via endovascular ou por cirurgia convencional. As lesões curtas no setor fêmoro-poplíteo devem, de início, ter preferencialmente uma abordagem endovascular^170,171,177^ .

**Tabela 6:** Exemplo de estratégia de revascularização infrainguinal em paciente de risco moderado e com veia boa para ponte.

GLASS	WIfI			
1	2	3	4
III				
II				
I				
	
	Cirurgia convencional			
	Indeterminado			
	Endovascular			
	Sem precisar revascularizar			

Há uma série de benefícios em utilizar um sistema integrado atualizado para classificação da isquemia dos membros inferiores:

Permite uma comunicação precisa entre os especialistas;
Dissemina a informação entre os médicos não especialistas;
Auxilia na análise dos casos em auditoria;
Permite classificação para pesquisas científicas.

A classificação WIfI é a mais atualizada, auxiliando na decisão clínica da revascularização, classificando a lesão e o paciente. O valor prognóstico dos níveis altos WIfI estão associados a desfechos de amputação e óbito, conforme estudos prospectivos não randomizados^178,179^ (Quadro 18).

**Quadro 18:** Recomendação na decisão do tratamento para o pacientes com ICAM.

**Recomendações da SBACV:**	
46 Recomenda-se utilizar sistema integrado de classificação de membros ameaçados (como WIfI) para classificar todos os pacientes ICAM que são candidatos ao salvamento de membro.	1C
47 Não se recomenda a realização de revascularização em membros de risco muito baixo (por exemplo, estágio WIfI 1), a menos que a ferida progrida ou não reduza em tamanho ≥ 50% dentro de 4 semanas, apesar do controle apropriado da infecção e da ferida.	2B
48 Em pacientes com isquemia crônica que ameaça o membro e lesões curtas no setor fêmoro-poplíteo (GLASS I e II) deve-se, inicialmente, adotar uma abordagem por via endovascular.	1B
49 Deve-se considerar a revascularização para pacientes com risco intermediário de amputação (por exemplo, estágios WIfI 2 e 3).	2C
50 A decisão para corrigir as obstruções de influxo, no mesmo momento ou de maneira estagiada à revascularização do membro, deve ter como base a gravidade da ameaça ao membro (estágio WIfI) e o estado clínico do paciente.	1C

Comentário Recomendação 45:

A indicação para revascularização deve levar em consideração o grau de isquemia, o grau de infecção, a extensão da lesão tecidual, a anatomia das obstruções arteriais e o estado clínico do paciente (classificação WIfI). As obstruções arteriais, principalmente as infrageniculares, são definidas por um exame de imagem de qualidade, sendo que a arteriografia ainda é o padrão-ouro. A avaliação clínica deve ser individualizada, sendo que pacientes com risco cirúrgico aceitável e com independência para suas atividades devem ser considerados para revascularização^180^ .

Comentário Recomendação 46:

Em pacientes com doença multinível e isquemia de baixo grau (WIfI grau 1) ou perda tecidual limitada (WIfI grau 1), a correção isolada da obstrução de aflúvio pode ser suficiente para a cicatrização. Entretanto, quando há obstruções proximais e riscos moderados ou altos de amputação, o procedimento de correção do aflúvio deve ser feito associado à revascularização infrainguinal. Tal correção do aflúvio pode ser feita de forma concomitante à revascularização ou estagiada, dependendo das condições disponíveis para a equipe cirúrgica e do grau de urgência em revascularizar o membro.

O planejamento pré-procedimento do tratamento em pacientes com ICAM deve incluir uma avaliação do risco cirúrgico e uma avaliação da disponibilidade da veia safena, sendo que em pacientes com uma veia safena magna de boa qualidade, a estratégia cirúrgica, como conduta inicial, foi associada a um risco 32% menor de eventos adversos graves nos membros ou morte, em relação à estratégia endovascular^171^ . A disponibilidade e a qualidade do conduto venoso autólogo, especialmente a veia safena magna, são critérios importantes para a cirurgia de derivação (ponte) e devem ser definidas antes que as decisões de revascularização sejam tomadas no caso de pacientes com risco de perda do membro^181-183^ . Outros segmentos venosos podem ser utilizados, como a safena parva ou veias dos membros superiores, porém, com resultados inferiores. O mapeamento por ultrassom auxilia no planejamento, considerando que os aparelhos atualmente estão mais disponíveis nos hospitais. A avaliação ultrassonográfica das veias pode ser feita pelo próprio cirurgião vascular, auxiliando na demarcação na pele, identificação da veia safena ou de outras veias saudáveis para o uso e otimizando o planejamento das incisões para vias de acesso (Quadro 19).

**Quadro 19:** Recomendação no mapeamento venoso para os pacientes com ICAM.

**Recomendação da SBACV:**	
51 O mapeamento venoso deve ser feito, quando disponível, em todos os pacientes com ICAM candidatos a cirurgias de derivação arterial (ponte).	1C

#### Endovascular

##### Território fêmoro-poplíteo

Os resultados da revascularização endovascular são heterogêneos e, em muitos aspectos, difíceis de quantificar. Ao separarmos por regiões, pode-se afirmar que o tratamento endovascular do segmento aorto-ilíaco tornou-se a opção preferencial quando anatomicamente adequado, devido à baixa incidência de morbidade e mortalidade em comparação com opções cirúrgicas abertas^151^ . A artéria femoral comum deve ser tratada preferencialmente pela cirurgia aberta. O desafio anatômico de uma localização flexível abaixo do ligamento inguinal e o potencial de cobrir a artéria femoral profunda durante a possível colocação do stent devem ser pesados contra os excelentes resultados de um procedimento direto e aberto. O consenso permanece para a intervenção cirúrgica, mas nos casos de anatomia desfavorável, como a necessidade de reintervenções ou pacientes de alto risco cirúrgico, pode-se optar pela técnica endovascular^184^ . O segmento da AFS tem múltiplos desafios, incluindo alongamento ou encurtamento, compressão e torção do vaso durante a atividade diária regular. Esses desafios dinâmicos podem ocasionar fraturas nos stents, o que pode levar à oclusão precoce e reestenose^185^ . Nas lesões curtas (< 25cm) desse território, a angioplastia com balão é comparável a stents convencionais, mas para segmentos mais longos, o uso de stent apresenta melhores taxas de perviedade primária e menores taxas de reintervenção^186^ ; contudo, a longo prazo, a perviedade primária é relativamente reduzida, devido à reestenose intra-stent^187^ . São considerados pacientes de risco cirúrgico elevado aqueles que possuem mortalidade perioperatória estimada em mais de 5%, ou uma expectativa de vida inferior a 50% em 2 anos.

A esses pacientes, pode ser oferecida inicialmente uma abordagem endovascular para o tratamento de lesões longas no setor fêmoro-poplíteo^168^ . Stents revestidos tendem a dificultar a proliferação neo-intimal, podendo oferecer alguma vantagem. Estudos não randomizados patrocinados pela indústria mostraram patência primária de 1 ano e perviedade secundária de 73 e 92% por cento^84^ . A lesão da artéria poplítea é frequentemente combinada com lesão da AFS e lesões que atingem a área da trifurcação infragenicular. Para essa região, os stents convencionais não apresentam boa performance e, sendo necessários (por exemplo dissecções complexas), os stents miméticos (com fios de nitinol entrelaçados) são recomendados no segmento da artéria poplítea abaixo do joelho (segmento P3), obtendo-se com esse dispositivo maior resistência à compressão extrínseca. Essa propriedade faz com que as fraturas desse tipo de stent sejam incomuns, incrementando a perviedade primária a longo prazo para lesões oclusivas no território poplíteo. Apesar disso, esse tipo de stent demanda uma preparação adequada do vaso, pois o preparo inadequado eleva taxas de reestenose e oclusão^188-191^ . Nos casos de reestenose, muitos estudos com stents e balões farmacológicos têm demonstrado excelentes resultados, com taxas de perviedade de 1 e 5 anos, de 86 e 66%, respectivamente, com melhora sintomática ocorrendo em 92 e 80%^192-194^ . Estudos randomizados e metanálises vêm demonstrando que balões farmacológicos também apresentaram mais benefícios, em comparação com balão convencional, na angioplastia primária^193^ , com resultados ainda melhores na redução da taxa de reestenose quando utilizadas concentrações mais altas de paclitaxel^195^ . Uma vantagem adicional na utilização somente da angioplastia com balão é o fato de não haver nenhum estímulo deixado nas artérias, diferentemente do que pode causar os stents autoexpansíveis, onde o metal impõe uma tensão (estímulo) constante na parede arterial (*chronic outward force*), que se traduz em maior risco de hiperplasia miointimal^196-199^ .

Existe uma preocupação com as taxas de mortalidade mais altas reportadas por estudos, associadas às tecnologias de eluição de drogas. No entanto, dados mais recentes de metanálises demonstram que o risco não é maior que o do uso de balões convencionais, independentemente da concentração do paclitaxel^84^ .

Com o advento dos balões farmacológicos, a utilização de stent focal fica mais restrita a áreas com dissecção e/ou retração elástica imediata (*recoil*). O conceito do stent focal é derivado de evidências indiretas e ainda está sendo avaliado em estudos que aguardam finalização^197^ (Quadro 20).

**Quadro 20:** Recomendação na indicação do tratamento endovascular do território fêmoro-poplíteo.

**Recomendações da SBACV:**	
52 Considerar o tratamento endovascular de doença artéria femoral comum somente em pacientes selecionados que são considerados de alto risco cirúrgico ou que tenham uma virilha hostil.	2C
53 A angioplastia com balão (convencional ou farmacológico) deve ser prioridade na artéria poplítea. O implante de stent opcional é recomendado preferencialmente para tratamento de lesões em que houve dissecção complexa e/ou recolhimento elástico maior que 30%. Nesses casos, especialmente no segmento P3, o stent mimético é a melhor opção.	1C
54 Recomenda-se que a colocação de stent autoexpansível seja restrita à colocação de stent focal (*spotting stent*), ou seja, em regiões de recolhimento elástico maior que 30%, dissecção complexa ou calcificação excêntrica com estenose maior que 30%. O implante de stent deve ser considerado somente após angioplastia com balão (convencional e/ou farmacológico).	1B
55 Os balões farmacológicos podem ser utilizados para o tratamento de reestenose intra-stent nas lesões fêmoro-poplíteo.	2A
56 Em pacientes com risco cirúrgico elevado e lesões longas (>25cm) no setor fêmoro-poplíteo, a revascularização por via endovascular pode ser considerada.	2C

Uma metanálise comparou 14 modalidades diferentes de tratamento: aterectomia, braquiterapia, crioplastia, *scoring balloon*, balões farmacológicos, stents de nitinol, stents recobertos e combinações desses. Foi demonstrado que o stent farmacológico e o stent revestido são as melhores modalidades de tratamento, em 12 e 24 meses, quanto aos desfechos de reestenose e revascularização da lesão alvo^200^ . A reestenose é o problema de maior preocupação associado aos procedimentos endovasculares na região fêmoro-poplítea. Muitas abordagens diferentes foram desenvolvidas para reduzir a sua ocorrência, como a utilização de stent, com redução nas taxas de reestenose em comparação com a angioplastia utilizando balão isoladamente. As taxas de perviedade primária, com uso de stent, particularmente após o tratamento de segmentos mais longos, foram de apenas 60 a 70% após 1 ano, e apenas 30 a 60% após 2 anos^201^ . A estratégia de angioplastia e implante de stent farmacológico pode ser considerada para tratamento de lesões curtas no setor fêmoro-poplíteo, com o objetivo de reduzir a taxa de reestenose no segmento tratado. Isso levou ao desenvolvimento de stents autoexpansíveis com eluição de drogas que melhoram as taxas de perviedade primária para 74,8% após 2 anos^202,203^ e redução maior que 40% do risco relativo para reestenose^204^ . Recomenda-se preparar adequadamente o vaso a ser tratado, com pré-dilatação visando a melhorar a distribuição da droga no caso dos balões e stents farmacológicos ou de melhorar a liberação dos stents autoexpansíveis com expansão completa destes. Esse cuidado pode aumentar a perviedade a curto e a longo prazo do segmento submetido a tratamento. Para o tratamento de regiões altamente calcificadas, pode ser necessário a utilização de dispositivos para preparação do vaso, com a retirada da placa (aterótomos) ou com a modificação da placa com uso de *scoring balloons* ^108,189,205^ .

##### Território infrapoplíteo

A terapia endovascular abaixo do joelho tem sido amplamente utilizada nos últimos anos, porém, a qualidade dos resultados clínicos para lesões infrapoplíteas ainda é insatisfatória em comparação com lesões acima do joelho, devido aos desafios anatômicos e opções limitadas de dispositivos específicos^206^ . O tratamento endovascular infrapoplíteo tem sido associado a uma alta incidência de reestenose do vaso tratado, onde as taxas de perviedade primária variam de 22 a 92% em 1 ano^207^ . No entanto, apesar das altas taxas de reestenose ou oclusão, o salvamento do membro pode ser obtido e mantido, mesmo com taxas relativamente baixas de perviedade dos vasos.

Análises sistemáticas da literatura revelam que tanto o tratamento endovascular quanto a cirurgia aberta produzem uma taxa de salvamento de membro de aproximadamente 80% em 3 anos^208^ . As melhorias técnicas recentes contribuíram para uma alta taxa de sucesso em recanalizações, mesmo em oclusões infrapoplíteas longas (> 10 cm) com isquemia crônica que ameaça o membro^209^ .

O uso de balão farmacológico para lesões infrapoplíteas, no seu início, mostrou-se inferior ao uso do balão convencional. Estudo prospectivo controlado, comparando angioplastias em artérias distais dos membros inferiores utilizando-se balões eluídos com paclitaxel *versus* balão convencional, falhou em confirmar vantagens para esse tipo de dispositivo^210^ , demonstrando que após 1 ano os resultados não apresentaram diferença entre os tratamentos. Além do mais, houve tendência a uma maior taxa de amputações com o uso do balão farmacológico (8,8% *versus* 3,6%). Esses balões foram retirados do mercado como consequência de outros estudos, os quais demonstravam que, aparentemente, o problema técnico não estava na droga, mas sim no *design* do balão e no processo de produção. No estudo randomizado BIOLUX P-II, realizado nos pacientes com claudicação e isquemia crônica que ameaça o membro, um novo balão farmacológico revestido com paclitaxel foi avaliado em relação ao balão convencional, para tratamento de lesões reestenóticas ou mesmo das artérias nativas infrapoplíteas em pacientes com claudicação e isquemia crítica do membro. O desfecho primário de segurança, composto de mortalidade por todas as causas, amputação maior da extremidade, trombose da lesão alvo e revascularização do vaso alvo em 30 dias, foi de 0% no grupo balão com droga *versus* 8,3% no grupo balão convencional (p = 0,239). O desfecho primário de desempenho, como perda de perviedade em 6 meses e amputações maiores da extremidade, foi mais significativo no grupo que utilizou balão sem droga durante 12 meses de acompanhamento, provando que o balão farmacológico era seguro e eficaz em lesões infrapoplíteas^211,212^ . Em outro estudo, 208 pacientes com claudicação grave (38,6%) ou com isquemia crônica que ameaça o membro (61,4%) foram analisados retrospectivamente. A amostra foi composta por 2/3 com lesões-alvo de oclusões totais, sendo que 17,8% de todos os pacientes, apresentavam oclusão das três artérias infrapopliteas. Nos resultados, 39 amputações em 31 membros foram necessárias; no entanto, 17 dessas foram amputações menores abaixo do tornozelo, ocorrendo apenas 9 (4,1%) amputações maiores. Na coorte completa, houve melhora de pelo menos uma categoria de Rutherford em 130 (59,1%) membros após 1 ano ou na última avaliação, com 104 (80,0%) desses membros apresentando melhora de duas categorias, demostrando uma promessa terapêutica em um estado de doença em que novas opções de tratamento são necessárias^213^ .

Estudos randomizados controlados verificaram se a angioplastia com balão ou a angioplastia com stent convencional são indicadas nas lesões dos vasos infrapoplíteos, mas falharam em evidenciar a superioridade do stent primário^214^ . Uma metanálise de 16 ensaios clínicos não randomizados não demonstrou vantagem do implante de stent primário de metal sobre a angioplastia com balão isolado, mas mostrou uma tendência de melhores resultados com o implante de stents farmacológicos^215^ . No entanto, esses resultados só foram obtidos em lesões focais (< 3 cm) em segmentos adequados para implante de stent.

Estudos comparativos entre stents convencionais e stents farmacológicos mostraram taxas de perviedade primária de 1 ano em grupos mistos de pacientes com claudicação intermitente e isquemia crítica, variando de 48 e 66% em relação à angioplastia por balão e sem implante de stent. Em uma metanálise de estudos randomizados, que investigou os resultados da revascularização percutânea com stent farmacológico primário em pacientes com doença aterosclerótica das artérias infrapoplíteas em comparação com angioplastia com balão simples ou stents convencionais, um total de 611 pacientes de 5 ensaios evidenciaram durante acompanhamento médio de 12 meses que o uso de stent farmacológico reduziu o risco de reestenose da lesão-alvo e amputação, sem diferença significativa em termos de óbito^216^ (Quadro 21).

**Quadro 21:** Recomendação na indicação do tratamento endovascular infrapoplíteo para os pacientes com ICAM.

**Recomendações da SBACV:**	
57 Em pacientes com isquemia crônica que ameaça o membro e lesões vasculares infrapoplíteas, o tratamento endovascular é recomendado como terapia de primeira linha.	1B

Análise comparativa na revascularização de reestenoses infrapoplíteas em 161 pacientes usando stents farmacológico com sirolimus mostrou uma tendência a menor taxa de amputação menor de 2,6% no seguimento de 3 anos *versus* 12,2% com o uso de stent convencional^217^ . Duas metanálises de estudos randomizados apresentaram números semelhantes em 1 ano após o implante de stents farmacológicos^218^ . O uso de stents com eluição de sirolimus após angioplastia com balão insuficiente resultou em taxas de reestenose e reintervenção reduzidas em comparação ao uso de stent sem droga, mas sem alteração quanto à taxa de amputações^219^ .

Assim, na doença focal (lesões curtas) das artérias infrapoplíteas, a terapêutica com stent farmacológico reduz o risco de reintervenção e amputação em comparação com a angioplastia com balão simples ou implante de stent convencional, sem qualquer impacto na mortalidade e na classe de Rutherford no seguimento de 1 ano^218,220^ .

A influência do uso de stent farmacológico nos desfechos clínicos de salvamento de membros e sobrevida livre de amputação ainda não foi suficientemente explorada. Além disso, sua aplicação é sempre limitada devido ao seu comprimento relativamente curto e ao risco de esmagamento em regiões de maior movimento e flexão. Dados clínicos para lesões infrapoplíteas ainda são limitados, e resultados de acompanhamento a longo prazo dos grandes estudos randomizados são necessários para entender melhor a relevância da técnica endovascular nos resultados clínicos. A dificuldade de resultados na manutenção da perviedade nas lesões infrapoplíteas após a intervenção é mais difícil em comparação com as lesões acima do joelho, devido à peculiaridade das características de lesão, como diâmetros menores, fluxo sanguíneo mais lento, presença de lesões com grande acúmulo de cálcio na camada média e nas lesões mais longas^221^ , tornando esse um desafio na busca de um resultado ainda melhor (Quadro 22).

**Quadro 22:** Recomendação na utilização de balão e/ou stent farmacológico.

**Recomendações da SBACV:**	
58 A preparação adequada do vaso é recomendada para a aplicação bem-sucedida de balão farmacológico ou stent autoexpansível.	1B
59 O implante de stent farmacológico pode ser considerado para tratamento de lesões curtas no setor fêmoro-poplíteo.	2B
60 O tratamento de lesões do território infrapoplíteo (longas e complexas) com balões farmacológicos exige pré-dilatação como recomendação padrão do tratamento.	2B

#### Cirurgia de revascularização aberta

Na doença aterosclerótica das extremidades inferiores, quando estudada como fonte da queixa do paciente, deve-se avaliar sempre o segmento vascular proximal, na prática chamado de aflúvio, e sua porção mais distal (deflúvio) do território acometido.

As cirurgias abertas de revascularização estão associadas à incidência baixa, mas significativa, de complicações operatórias das feridas e dos enxertos utilizados, que podem ser evitadas pela via endovascular. A doença aorto-ilíaca por exemplo, é usualmente tratada em um primeiro momento por meio de técnicas endovasculares (angioplastia com ou sem stent), mas não sendo isso possível, ou mesmo falhando, a revascularização cirúrgica ou até mesmo a utilização de técnicas híbrida pode ser considerada em pacientes selecionados com perfis de risco aceitáveis^222^ . A doença infrainguinal pode ser tratada com pontes geralmente originadas da artéria femoral comum (aflúvio) terminando nos vasos poplíteos acima ou abaixo do joelho (tibial ou pedioso). Os procedimentos de revascularização aberta podem ser considerados para oclusões de segmento longo, que não podem ser tratadas apenas com técnicas endovasculares ou reparos locais, sendo sua perviedade condicionada à qualidade do enxerto e material utilizado – as veias autógenas (safena ou outra) fornecem melhores resultados, com taxas de perviedade que variam de 60 a 80% em 5 anos^171,223^ . A perviedade diminui significativamente com revascularizações mais distais para artéria tibial ou pediosa, e, portanto, essas só devem ser realizadas para isquemia crônica que ameace o membro, devendo-se evitar o uso de prótese neste nível, pela baixa taxa de perviedade.

Há diversas técnicas possíveis para abordagem cirúrgica, aberta ou endovascular, no paciente portador de DAPMI. A decisão de adotar uma técnica em prol de outra deve ser individualizada, não só a partir dos parâmetros clínicos do paciente, mas também moldada na estrutura disponível e *expertise* da equipe para realizar o procedimento (Figura 1)^171,178,182^ . Existem poucos ERCs sobre tratamento cirúrgico *versus* endovascular nas isquemias crônicas que ameaçam o membro, não sendo encontradas diferenças na sobrevida livre de amputação em 1 ou 5 anos. No entanto, a análise post-hoc do estudo *Bypass versus angioplasty in severe ischaemia of the leg* (BASIL) sugeriu maior sobrevida livre de amputação com cirurgia de revascularização^224^ . Esse achado corrobora a durabilidade superior da revascularização cirúrgica em relação à angioplastia com balão em pacientes com isquemia crônica que ameaça o membro. Nas lesões fêmoro-poplítea e infrapoplítea, em que a revascularização aberta é considerada, a veia safena magna deve ser a primeira escolha, pois apresenta taxas de perviedade muito maiores e com reintervenções muito menores do que os substitutos artificiais (PTFE e Dacron)^171,224-226^ . Com o uso de próteses, aumenta-se também a incidência de infecções, mas em casos selecionados há a possibilidade do uso de material protético como substituto à cirurgia aberta^224,225^ . Recomenda-se usar fístulas distais e anéis venosos para ampliar essa perviedade, além de alguns estudos recomendarem o uso da dupla antiagregação plaquetária nesses pacientes para melhorar a perviedade do substituto protético^103,227^ (Quadro 23).

**Figura 1:** Algoritmo do tratamento da claudicação.

**Quadro 23:** Recomendação na programação da revascularização aberta para o paciente com ICAM.

**Recomendações da SBACV:**	
61 Recomenda-se que, ao programar uma revascularização na DAPMI, a decisão da estratégia de revascularização endovascular, aberta ou híbrida, seja definida com base no padrão anatômico da doença, grau de isquemia, condição clínica do paciente, durabilidade do procedimento, disponibilidade de recursos e experiência do cirurgião.	1C
62 Recomenda-se o uso de material protético sintético em pacientes com DAP infrainguinal com isquemia crônica que ameaça o membro, quando não há possibilidade do tratamento endovascular e nem veias autólogas disponíveis como substitutos.	1C
63 Recomenda-se o uso de material protético sintético associado a interposição de anel venoso, em pacientes com DAP infragenicular com isquemia crônica que ameaça o membro, quando não há possibilidade do tratamento endovascular e nem veias autólogas disponíveis como substitutos.	2 C

No intuito de melhorar os resultados quando se faz necessário o uso de substituto protético sintético, recomenda-se, associar na confecção da anastomose distal infragenicular, a interposição de anéis venosos pois devido a sua área transversal mais ampla, auxilia na melhora da dinâmica de fluxo local, tanto pela formação de um vórtice coeso no conduíte^228^ , assim como em manter um fluxo mais constante na anastomose^229^ com menor resistência ao fluxo no alvo distal^230^ , desta forma ocorre uma redistribuição das forças na formação da hiperplasia intimal^231^ alterando a tensão de cisalhamento da parede e reduzindo o estímulo para hiperplasia intimal^232-234^ . Outra vantagem do seu uso é permitir uma anastomose mais fácil, particularmente para pequenos vasos^235^ , além de auxiliar na preservação do fluxo em caso de trombose do enxerto^236^ . Porém apesar desses benefícios, principalmente nos pacientes dialíticos, naqueles com necessidade de anastomoses mais distais (infrageniculares) e com isquemias crônicas que ameaça o membro, os resultados que avaliam o desempenho dos adjuntos venosos não têm sido uniformes e até mesmo contraditórios, com relação ao salvamento do membro, com muitos estudos corroborando para um aumento somente na perviedade do enxerto^237-239^ .

Apesar do uso crescente de intervenções endovasculares, a cirurgia aberta continua sendo uma importante opção terapêutica em pacientes selecionados com DAPMI. A endarterectomia é uma técnica na qual a placa é removida diretamente da artéria, para tratar estenoses ou oclusões de segmentos curtos, sendo posteriormente fechada com uma veia ou angioplastia com remendo heterólogo (pericárdio bovino, por exemplo) ou sintético para aumentar o diâmetro da artéria. É muito utilizada no tratamento para melhorar o aflúvio ou deflúvio, em conjunto com procedimentos cirúrgicos de pontes. A artéria femoral comum é a artéria mais comum da extremidade inferior tratada com endarterectomia, que pode ser realizada como um único procedimento. Seu principal objetivo é estabelecer uma linha direta de fluxo arterial para a artéria femoral profunda, tendo em vista que a preservação do fluxo nessa artéria apresenta grande impacto na preservação do membro no seguimento de longo prazo. A técnica também pode ser utilizada de forma associada no momento da revascularização para aumentar o aflúvio da ponte quando necessário, contribuindo para melhorar as taxas de perviedade da revascularização (Figura 2)^168,224^ .

**Figura 2:** Algoritmo do tratamento da isquemia crônica que ameaça o membro.

De maneira geral, o sistema de classificação GLASS é preditivo de isquemia crônica que ameaça o membro, bem como falência técnica imediata e da perviedade após técnica endovascular. Uma metanálise sugere que os pacientes em estágios GLASS avançados terão maior benefício quando submetidos à cirurgia com ponte do que com o uso de terapia endovascular^169^ . A presença de um substituto adequado para uma revascularização aberta representa uma questão decisiva no sucesso da revascularização. A veia safena magna de calibre adequado permanece como substituto de escolha para a cirurgia aberta nesse território, demonstrando durabilidade superior quando comparada com todos os outros substitutos: prótese, veia safena parva, veia de braço, seguimentos venosos compostos e a própria AFS endarterectomizada^240^ (Quadro 24).

**Quadro 24:** Recomendação na programação da técnica de revascularização aberta para o paciente com ICAM.

**Recomendações da SBACV:**	
64 Recomenda-se realizar endarterectomia aberta da artéria femoral comum com arterioplastia utilizando remendo, com ou sem extensão para artéria femoral profunda, em pacientes com ICAM com doença hemodinamicamente significativa (> 50% de estenose) das artérias femoral comum e profunda.	1B
65 Recomenda-se que a ponte para a artéria poplítea (quando indicado) seja realizada com veia autógena, preferencialmente, ao invés de enxerto sintético para o tratamento de claudicação intermitente.	1A
66 Recomenda-se que a derivação cirúrgica para artéria poplítea ou infrapoplítea seja realizada com veia autógena para os quadros de isquemia crônica que ameaça o membro.	1A
67 Quando a ponte acima do joelho é indicada, o uso de enxerto protético deve ser considerado, mas somente na ausência de qualquer veia autóloga.	2A
68 Em pacientes que não são de alto risco cirúrgico, a cirurgia de revascularização é indicada para lesões fêmoro-poplítea longas (> 25 cm) quando uma veia autóloga está disponível e a expectativa de vida é > 2 anos.	1A

Embora o tratamento endovascular da ICAM esteja disponível há mais de 20 anos e seja um método importante no tratamento dos pacientes, até o presente momento o benefício clínico e custo efetivo do emprego de tecnologias endovasculares ainda não estão claros^241,242^ . Até o momento, o BASIL e o BEST-CLI permanecem como os únicos estudos randomizados controlados onde se comparam os resultados entre o tratamento aberto e endovascular, permanecendo a ideia de que para pacientes com boa expectativa de vida, com bom substituto autólogo, o tratamento aberto deva ser considerado primeiro^97,171,182,225^ .

A reconstrução arterial de membros inferiores continua sendo a pedra angular no salvamento dos membros por isquemia crônica que ameaça o membro. Nas últimas 2 décadas, os progressos na avaliação e seleção dos pacientes têm resultado em abordagem mais agressiva e com mais sucesso, especialmente nas reconstruções ultradistais. Embora a perviedade das revascularizações e taxas de salvamento de membros demonstrem contínuos avanços, são necessários mais estudos para avaliar o custo efetividade das revascularizações infrainguinais, bem como a qualidade de vida dos pacientes. Importante frisar que é necessário reconhecer as limitações e os benefícios das técnicas consagradas abertas e também as técnicas minimamente invasivas endovasculares, para entender claramente a sua complementaridade e jamais competitividade entre elas^171^ .

## LISTA DE ABREVIAÇÕES

AAS: ácido acetilsalicílico

AFS: artéria femoral superficial

AVC: acidente vascular cerebral

AVK: antagonista de vitamina K

CEN: cigarro eletrônico contendo nicotina

CI: claudicação intermitente

CV: cardiovascular

DAC: doença arterial coronariana

DAP: doença arterial periférica

DAPMI: doença arterial periférica dos membros inferiores

DCV: doença cardiovascular

DM: diabetes melito

DOAC: do inglês, *direct oral anticoagulants* (anticoagulantes de ação direta)

DPP-4: inibidores seletivos baseados em incretina de dipeptidil peptidase 4

DRC: doença renal crônica

ERC: estudos randomizados controlados

FA: fibrilação atrial

FR: fator de risco

GLASS: do inglês, Global Limb Anatomic Staging System (Sistema de Estadiamento Anatômico Global de Membros)

GRADE: do inglês, Grading of Recommendations Assessment, Development and Evaluation

HAS: hipertensão arterial sistêmica

IAM: infarto agudo do miocárdio

IC: intervalo de confiança

ICAM: isquemia crônica que ameaça o membro

IHB: índice hálux-braço

INR: do inglês, *international normalized ratio*

ISTH: International Society on Thrombosis and Haemostasis

ITB: índice tornozelo-braço

MACE: do inglês, *composite of myocardial infarction, stroke, or cardiovascular death* (associação de morte, infarto do miocárdio ou acidente vascular cerebral)

MALE: do inglês, *major adverse limb events* (associação de eventos adversos graves nos membros)

MMII: membros inferiores

OD: *odds ratio*

PA: pressão arterial

PAS: pressão arterial sistólica

RR: risco relativo

SBACV: Sociedade Brasileira de Angiologia e Cirurgia Vascular

SGLT 2: sodium-glucose co-transporter-2

TAD: terapia antiplaquetária dupla

TAS: terapia antiplaquetária simples

TF: tronco tibiofibular

TLR: do inglês, *target lesion revascularization*

TRN: terapia de reposição de nicotina

TVP: trombose venosa profunda

WIfI: do inglês, *wound, ischemia and foot infection*
